# Supplementary material for: Wildfire, deforestation and health in tropical rainforest areas: a scoping review protocol
Source: BMJ Open. 2024 May 8;14(5):e082381. doi: 10.1136/bmjopen-2023-082381 (PMC11086528; doi:10.1136/bmjopen-2023-082381)
Supplement: Supplementary data [file bmjopen-2023-082381supp001.pdf]

Appendices

Appendix I – Literature search

MEDLINE/PubMed

("wildfires"[MeSH Terms] OR "wildfires"[Text Word] OR "Wildfire"[All Fields] OR "Wildland Fires"[All Fields] OR "Brush Fires"[All Fields] OR "Brush Fire"[All Fields] OR "Forest Fires"[All Fields] OR "fire forest"[All Fields] OR "fires forest"[All Fields] OR "Forest Fire"[All Fields] OR "Wild Fires"[All Fields] OR "Wild Fire"[All Fields] OR "Fires"[MeSH Terms] OR "Fires"[Text Word] OR "Fire"[All Fields] OR "fire outbreaks"[All Fields] OR "Deforestation"[All Fields] OR "Grassfire"[All Fields] OR "prescribed burn"[All Fields] OR "prescribed fire"[All Fields]) AND ("Tropical Climate"[MeSH Terms] OR "Tropical Climate"[Text Word] OR "climate tropical"[All Fields] OR "climates tropical"[All Fields] OR "Tropical Climates"[All Fields] OR "Rainforest"[MeSH Terms] OR "Rainforest"[Text Word] OR "Rainforests"[All Fields] OR "Rain Forest"[All Fields] OR "forest rain"[All Fields] OR "Rain Forests"[All Fields] OR "Tropical Rainforest"[All Fields] OR "rainforest tropical"[All Fields] OR "rainforests tropical"[All Fields] OR "Tropical Rainforests"[All Fields] OR "Amazon"[All Fields] OR "Brazil"[All Fields] OR "Argentina"[All Fields] OR "Peru"[All Fields] OR "Ecuador"[All Fields] OR "Bolivia"[All Fields] OR "Colombia"[All Fields] OR "Venezuela"[All Fields] OR "Guyana"[All Fields] OR "Suriname"[All Fields] OR "French Guiana"[All Fields] OR "Paraguay"[All Fields] OR "Panama"[All Fields] OR "El Salvador"[All Fields] OR "Belize"[All Fields] OR "Costa Rica"[All Fields] OR "El Salvador"[All Fields] OR "Guatemala"[All Fields] OR "Honduras"[All Fields] OR "Nicaragua"[All Fields] OR "Panama"[All Fields] OR "Anguilla"[All Fields] OR "Antigua and Barbuda"[All Fields] OR "Aruba"[All Fields] OR "Bahamas"[All Fields] OR "Barbados"[All Fields] OR "British Virgin Islands"[All Fields] OR "Cayman Islands"[All Fields] OR "Cuba"[All Fields] OR "Dominica"[All Fields] OR "Dominican Republic"[All Fields] OR "Grenada"[All Fields] OR "Guadeloupe"[All Fields] OR "Haiti"[All Fields] OR "Jamaica"[All Fields] OR "Martinique"[All Fields] OR "Montserrat"[All Fields] OR "Netherlands Antilles"[All Fields] OR "Puerto Rico"[All Fields] OR "Saint Barthelemy"[All Fields] OR "Saint Kits and Nevis"[All Fields] OR "Saint Lucia"[All Fields] OR "Saint Martin"[All Fields] OR "Saint Vincent and the Grenadines"[All Fields] OR "Trinidad and Tobago"[All Fields] OR "United States Virgin Islands"[All Fields] OR "Mexico"[All Fields] OR "Hawaii"[All Fields] OR "Cape Verde"[All Fields] OR "Sao Tome and Principe"[All Fields] OR "Gambia"[All Fields] OR "Senegal"[All Fields] OR "Guinea-Bissau"[All Fields] OR "Guinea"[All Fields] OR "Sierra Leone"[All Fields] OR "Liberia"[All Fields] OR "Ivory Coast"[All Fields] OR "Ghana"[All Fields] OR "Togo"[All Fields] OR "Benin"[All Fields] OR "Nigeria"[All Fields] OR "Cameroon"[All Fields] OR "Central African Republic"[All Fields] OR "South Sudan"[All Fields] OR "Ethiopia"[All Fields] OR "Equatorial Guinea"[All Fields] OR "Gabon"[All Fields] OR "Congo"[All Fields] OR "Democratic Republic of the Congo"[All Fields] OR "Uganda"[All Fields] OR "Rwanda"[All Fields] OR "Burundi"[All Fields] OR "Kenya"[All Fields] OR "Somalia"[All Fields] OR "Tanzania"[All Fields] OR "Zambia"[All Fields] OR "Mozambique"[All Fields] OR "Madagascar"[All Fields] OR "Seychelles"[All Fields] OR "Mauritius"[All Fields] OR "Comoros"[All Fields] OR "Botswana"[All Fields] OR "Malawi"[All Fields] OR "Brunei"[All Fields] OR "Burma"[All Fields] OR "Myanmar"[All Fields] OR "Cambodia"[All Fields] OR "East Timor"[All Fields] OR

"Indonesia"[All Fields] OR "Laos"[All Fields] OR "Malaysia"[All Fields] OR "Philippines"[All Fields] OR "Singapore"[All Fields] OR "Thailand"[All Fields] OR "Vietnam"[All Fields] OR "India"[All Fields] OR "Papua New Guinea"[All Fields] OR "Australia"[All Fields] OR "Solomon Islands"[All Fields] OR "Vanuatu"[All Fields] OR "New Caledonia"[All Fields]) AND ("cardiovascular diseases"[MeSH Terms] OR "cardiovascular diseases"[Text Word] OR "Cardiovascular Disease"[All Fields] OR "disease cardiovascular"[All Fields] OR "Major Adverse Cardiac Events"[All Fields] OR "Cardiac Events"[All Fields] OR "Cardiac Event"[All Fields] OR "event cardiac"[All Fields] OR "Adverse Cardiac Event"[All Fields] OR "Adverse Cardiac Events"[All Fields] OR "cardiac events adverse"[All Fields] OR "chemically induced disorders"[MeSH Terms] OR "chemically induced disorders"[Text Word] OR "chemically induced disorders"[All Fields] OR "Chemically-Induced Disorder"[All Fields] OR "congenital, hereditary, and neonatal diseases and abnormalities"[MeSH Terms] OR "congenital hereditary and neonatal diseases and abnormalities"[Text Word] OR "Congenital Disorders"[All Fields] OR "disorder congenital"[All Fields] OR "disorders congenital"[All Fields] OR "Neonatal Diseases and Abnormalities"[All Fields] OR "Digestive System Diseases"[MeSH Terms] OR "Digestive System Diseases"[Text Word] OR "Digestive System Disease"[All Fields] OR "Digestive System Disorders"[All Fields] OR "Digestive System Disorder"[All Fields] OR "system disorders digestive"[All Fields] OR "Hepatobiliary Disorders"[All Fields] OR "Hepatobiliary Disorder"[All Fields] OR "Hepatobiliary Diseases"[All Fields] OR "Hepatobiliary Disease"[All Fields] OR "Disorders of Environmental Origin"[MeSH Terms] OR "Disorders of Environmental Origin"[Text Word] OR "Endocrine System Diseases"[MeSH Terms] OR "Endocrine System Diseases"[Text Word] OR "disease endocrine system"[All Fields] OR "diseases endocrine system"[All Fields] OR "Endocrine System Disease"[All Fields] OR "system disease endocrine"[All Fields] OR "system diseases endocrine"[All Fields] OR "Endocrine Diseases"[All Fields] OR "disease endocrine"[All Fields] OR "diseases endocrine"[All Fields] OR "Endocrine Disease"[All Fields] OR "Diseases of Endocrine System"[All Fields] OR "Eye Diseases"[MeSH Terms] OR "Eye Diseases"[Text Word] OR "Eye Disease"[All Fields] OR "Eye Disorders"[All Fields] OR "Eye Disorder"[All Fields] OR "Hemic and Lymphatic Diseases"[MeSH Terms] OR "Hemic and Lymphatic Diseases"[Text Word] OR "Blood and Lymphatic System Disorders"[All Fields] OR "Immune System Diseases"[MeSH Terms] OR "Immune System Diseases"[Text Word] OR "disease immune system"[All Fields] OR "Immune System Disease"[All Fields] OR "Immunologic Diseases"[All Fields] OR "disease immunologic"[All Fields] OR "Immunologic Disease"[All Fields] OR "Immunological Diseases"[All Fields] OR "disease immunological"[All Fields] OR "Immunological Disease"[All Fields] OR "Immune Diseases"[All Fields] OR "disease immune"[All Fields] OR "Immune Disease"[All Fields] OR "Diseases of Immune System"[All Fields] OR "Immune Disorders"[All Fields] OR "Immune Disorder"[All Fields] OR "Immune System Disorders"[All Fields] OR "disorder immune system"[All Fields] OR "Immune System Disorder"[All Fields] OR "Infections"[MeSH Terms] OR "Infections"[Text Word] OR "Infection and Infestation"[All Fields] OR "Infestation and Infection"[All Fields] OR "Infections and Infestations"[All Fields] OR "Infestations and Infections"[All Fields] OR "Infection"[All Fields] OR "Musculoskeletal Diseases"[MeSH Terms] OR "Musculoskeletal Diseases"[Text Word] OR "Musculoskeletal Disease"[All Fields] OR "Orthopedic Disorders"[All Fields] OR "Orthopedic Disorder"[All Fields] OR "Neoplasms"[MeSH Terms] OR "Neoplasms"[Text Word] OR "Tumor"[All Fields] OR "Neoplasm"[All Fields] OR "Tumors"[All Fields] OR "Neoplasia"[All Fields] OR "Neoplasias"[All Fields] OR "Cancer"[All Fields] OR "Cancers"[All Fields] OR "Malignant Neoplasm"[All Fields] OR "Malignancy"[All Fields] OR "Malignancies"[All Fields] OR "Malignant Neoplasms"[All Fields] OR "neoplasm malignant"[All Fields] OR "neoplasms malignant"[All Fields] OR "Benign

Neoplasms"[All Fields] OR "Benign Neoplasm"[All Fields] OR "neoplasms benign"[All Fields] OR "neoplasm benign"[All Fields] OR "Nervous System Diseases"[MeSH Terms] OR "Nervous System Diseases"[Text Word] OR "disease nervous system"[All Fields] OR "diseases nervous system"[All Fields] OR "Nervous System Disease"[All Fields] OR "Neurologic Disorders"[All Fields] OR "disorder neurologic"[All Fields] OR "disorders neurologic"[All Fields] OR "Neurologic Disorder"[All Fields] OR "Neurological Disorders"[All Fields] OR "disorder neurological"[All Fields] OR "disorders neurological"[All Fields] OR "Neurological Disorder"[All Fields] OR "Nervous System Disorders"[All Fields] OR "disorder nervous system"[All Fields] OR "disorders nervous system"[All Fields] OR "Nervous System Disorder"[All Fields] OR "Nutritional and Metabolic Diseases"[MeSH Terms] OR "Nutritional and Metabolic Diseases"[Text Word] OR "Occupational Diseases"[MeSH Terms] OR "Occupational Diseases"[Text Word] OR "disease occupational"[All Fields] OR "Occupational Disease"[All Fields] OR "Occupational Illnesses"[All Fields] OR "illnesses occupational"[All Fields] OR "diseases occupational"[All Fields] OR "Otorhinolaryngologic Diseases"[MeSH Terms] OR "Otorhinolaryngologic Diseases"[Text Word] OR "Otorhinolaryngological Disease"[All Fields] OR "Otorhinolaryngological Diseases"[All Fields] OR "Otolaryngological Diseases"[All Fields] OR "diseases otolaryngological"[All Fields] OR "Otolaryngological Disease"[All Fields] OR "Otorhinolaryngologic Disease"[All Fields] OR "ENT Diseases"[All Fields] OR "disease ent"[All Fields] OR "diseases ent"[All Fields] OR "ENT Disease"[All Fields] OR "Otolaryngologic Diseases"[All Fields] OR "disease otolaryngologic"[All Fields] OR "diseases otolaryngologic"[All Fields] OR "Otolaryngologic Disease"[All Fields] OR "pathological conditions, signs and symptoms"[MeSH Terms] OR "pathological conditions signs and symptoms"[Text Word] OR "Respiratory Tract Diseases"[MeSH Terms] OR "Respiratory Tract Diseases"[Text Word] OR "disease respiratory tract"[All Fields] OR "Respiratory Tract Disease"[All Fields] OR "Respiratory Diseases"[All Fields] OR "Respiratory System Diseases"[All Fields] OR "disease respiratory system"[All Fields] OR "Respiratory System Disease"[All Fields] OR "Skin and Connective Tissue Diseases"[MeSH Terms] OR "Skin and Connective Tissue Diseases"[Text Word] OR "Stomatognathic Diseases"[MeSH Terms] OR "Stomatognathic Diseases"[Text Word] OR "Stomatognathic Disease"[All Fields] OR "Mouth and Tooth Diseases"[All Fields] OR "Dental Diseases"[All Fields] OR "Dental Disease"[All Fields] OR "disease dental"[All Fields] OR "diseases dental"[All Fields] OR "Urogenital Diseases"[MeSH Terms] OR "Urogenital Diseases"[Text Word] OR "disease urogenital"[All Fields] OR "Urogenital Disease"[All Fields] OR "Genitourinary Diseases"[All Fields] OR "disease genitourinary"[All Fields] OR "Genitourinary Disease"[All Fields] OR "Wounds and Injuries"[MeSH Terms] OR "Wounds and Injuries"[Text Word] OR "Injuries and Wounds"[All Fields] OR "Wounds and Injury"[All Fields] OR "Injury and Wounds"[All Fields] OR "wounds injury"[All Fields] OR "Trauma"[All Fields] OR "Traumas"[All Fields] OR "injuries wounds"[All Fields] OR "research related injuries"[All Fields] OR "research related injuries"[All Fields] OR "Research-Related Injury"[All Fields] OR "Injuries"[All Fields] OR "Injury"[All Fields] OR "Wounds"[All Fields] OR "Wound"[All Fields] OR "Vital Statistics"[MeSH Terms] OR "Vital Statistics"[Text Word] OR "statistics vital"[All Fields] OR "Vital Statistics Registration"[All Fields] OR "Registration of Vital Statistics"[All Fields] OR "Vital Statistics Registrations"[All Fields] OR "registration vital statistics"[All Fields] OR "registrations vital statistics"[All Fields] OR "Patient Care"[MeSH Terms] OR "Patient Care"[Text Word] OR "care patient"[All Fields] OR "Informal care"[All Fields] OR "Informal cares"[All Fields] OR "care informal"[All Fields])

Date of search: August 18, 2023

Results: 2,296

Scopus

Search: ("wildfires" OR "wildfire" OR "wildland fires" OR "brush fires" OR "brush fire" OR "forest fires" OR "fire forest" OR "fires forest" OR "forest fire" OR "wild fires" OR "wild fire" OR "fires" OR "fire" OR "fire outbreaks" OR "deforestation" OR "grassfire" OR "prescribed burn" OR "prescribed fire")AND ("tropical climate" OR "climate tropical" OR "climates tropical" OR "tropical climates" OR "rainforest" OR "rainforests" OR "rain forest" OR "forest rain" OR "rain forests" OR "tropical rainforest" OR "rainforest tropical" OR "rainforests tropical" OR "tropical rainforests" OR "Amazon" OR "Brazil" OR "Argentina" OR "Peru" OR "Ecuador" OR "Bolivia" OR "Colombia" OR "Venezuela" OR "Guyana" OR "Suriname" OR "French Guiana" OR "Paraguay" OR "Panama" OR "El Salvador" OR "Belize" OR "Costa Rica" OR "El Salvador" OR "Guatemala" OR "Honduras" OR "Nicaragua" OR "Panama" OR "Anguilla" OR "Antigua and Barbuda" OR "Aruba" OR "Bahamas" OR "Barbados" OR "British Virgin Islands" OR "Cayman Islands" OR "Cuba" OR "Dominica" OR "Dominican Republic" OR "Grenada" OR "Guadeloupe" OR "Haiti" OR "Jamaica" OR "Martinique" OR "Montserrat" OR "Netherlands Antilles" OR "Puerto Rico" OR "Saint Barthelemy" OR "Saint Kits and Nevis" OR "Saint Lucia" OR "Saint Martin" OR "Saint Vincent and the Grenadines" OR "Trinidad and Tobago" OR "United States Virgin Islands" OR "Mexico" OR "Hawaii" OR "Cape Verde" OR "Sao Tome and Principe" OR "Gambia" OR "Senegal" OR "Guinea-Bissau" OR "Guinea" OR "Sierra Leone" OR "Liberia" OR "Ivory Coast" OR "Ghana" OR "Togo" OR "Benin" OR "Nigeria" OR "Cameroon" OR "Central African Republic" OR "South Sudan" OR "Ethiopia" OR "Equatorial Guinea" OR "Gabon" OR "Congo" OR "Democratic Republic of the Congo" OR "Uganda" OR "Rwanda" OR "Burundi" OR "Kenya" OR "Somalia" OR "Tanzania" OR "Zambia" OR "Mozambique" OR "Madagascar" OR "Seychelles" OR "Mauritius" OR "Comoros" OR "Botswana" OR "Malawi" OR "Brunei" OR "Burma" OR "Myanmar" OR "Cambodia" OR "East Timor" OR "Indonesia" OR "Laos" OR "Malaysia" OR "Philippines" OR "Singapore" OR "Thailand" OR "Vietnam" OR "India" OR "Papua New Guinea" OR "Australia" OR "Solomon Islands" OR "Vanuatu" OR "New Caledonia") AND ("cardiovascular diseases" OR "Cardiovascular Disease" OR "disease cardiovascular" OR "Major Adverse Cardiac Events" OR "Cardiac Events" OR "Cardiac Event" OR "event cardiac" OR "Adverse Cardiac Event" OR "Adverse Cardiac Events" OR "cardiac events adverse" OR "chemically induced disorders" OR "chemically induced disorders" OR "Chemically-Induced Disorder" OR "congenital, hereditary, and neonatal diseases and abnormalities" OR "Congenital Disorders" OR "disorder congenital" OR "disorders congenital" OR "Neonatal Diseases and Abnormalities" OR "Digestive System Diseases" OR "Digestive System Disease" OR "Digestive System Disorders" OR "Digestive System Disorder" OR "system disorders digestive" OR "Hepatobiliary Disorders" OR "Hepatobiliary Disorder" OR "Hepatobiliary Diseases" OR "Hepatobiliary Disease" OR "Disorders of Environmental Origin"OR "Endocrine System Diseases" OR "disease endocrine system" OR "diseases endocrine system" OR "Endocrine System Disease" OR "system disease endocrine" OR "system diseases endocrine" OR "Endocrine Diseases" OR "disease endocrine" OR "diseases endocrine" OR "Endocrine Disease" OR "Diseases of Endocrine System" OR "Eye Diseases" OR "Eye Disease" OR "Eye Disorders" OR "Eye Disorder" OR "Hemic and Lymphatic Diseases" OR "Blood and Lymphatic System Disorders" OR "Immune System Diseases" OR "disease immune system" OR "Immune System Disease" OR "Immunologic Diseases" OR "disease immunologic" OR "Immunologic Disease" OR "Immunological Diseases"

OR "disease immunological" OR "Immunological Disease" OR "Immune Diseases" OR "disease immune" OR "Immune Disease" OR "Diseases of Immune System" OR "Immune Disorders" OR "Immune Disorder" OR "Immune System Disorders" OR "disorder immune system" OR "Immune System Disorder" OR "Infections" OR "Infection and Infestation" OR "Infestation and Infection" OR "Infections and Infestations" OR "Infestations and Infections" OR "Infection" OR "Musculoskeletal Diseases" OR "Musculoskeletal Disease" OR "Orthopedic Disorders" OR "Orthopedic Disorder" OR "Neoplasms" OR "Tumor" OR "Neoplasm" OR "Tumors" OR "Neoplasia" OR "Neoplasias" OR "Cancer" OR "Cancers" OR "Malignant Neoplasm" OR "Malignancy" OR "Malignancies" OR "Malignant Neoplasms" OR "neoplasm malignant" OR "neoplasms malignant" OR "Benign Neoplasms" OR "Benign Neoplasm" OR "neoplasms benign" OR "neoplasm benign" OR "Nervous System Diseases" OR "disease nervous system" OR "diseases nervous system" OR "Nervous System Disease" OR "Neurologic Disorders" OR "disorder neurologic" OR "disorders neurologic" OR "Neurologic Disorder" OR "Neurological Disorders" OR "disorder neurological" OR "disorders neurological" OR "Neurological Disorder" OR "Nervous System Disorders" OR "disorder nervous system" OR "disorders nervous system" OR "Nervous System Disorder" OR "Nutritional and Metabolic Diseases" OR "Occupational Diseases" OR "disease occupational" OR "Occupational Disease" OR "Occupational Illnesses" OR "illnesses occupational" OR "diseases occupational" OR "Otorhinolaryngologic Diseases" OR "Otorhinolaryngological Disease" OR "Otorhinolaryngological Diseases" OR "Otolaryngological Diseases" OR "diseases otolaryngological" OR "Otolaryngological Disease" OR "Otorhinolaryngologic Disease" OR "ENT Diseases" OR "disease ent" OR "diseases ent" OR "ENT Disease" OR "Otolaryngologic Diseases" OR "disease otolaryngologic" OR "diseases otolaryngologic" OR "Otolaryngologic Disease" OR "pathological conditions, signs and symptoms" OR "Respiratory Tract Diseases" OR "disease respiratory tract" OR "Respiratory Tract Disease" OR "Respiratory Diseases" OR "Respiratory System Diseases" OR "disease respiratory system" OR "Respiratory System Disease" OR "Skin and Connective Tissue Diseases" OR "Stomatognathic Diseases" OR "Stomatognathic Disease" OR "Mouth and Tooth Diseases" OR "Dental Diseases" OR "Dental Disease" OR "disease dental" OR "diseases dental" OR "Urogenital Diseases" OR "disease urogenital" OR "Urogenital Disease" OR "Genitourinary Diseases" OR "disease genitourinary" OR "Genitourinary Disease" OR "Wounds and Injuries" OR "Injuries and Wounds" OR "Wounds and Injury" OR "Injury and Wounds" OR "wounds injury" OR "Trauma" OR "Traumas" OR "injuries wounds" OR "research related injuries" OR "research related injuries" OR "Research-Related Injury" OR "Injuries" OR "Injury" OR "Wounds" OR "Wound" OR "Vital Statistics" OR "statistics vital" OR "Vital Statistics Registration" OR "Registration of Vital Statistics" OR "Vital Statistics Registrations" OR "registration vital statistics" OR "registrations vital statistics" OR "Patient Care" OR "care patient" OR "Informal care" OR "Informal cares" OR "care informal")

Date of search: August 18, 2023

Results: 1,604

Biblioteca Virtual em Saúde

#1 "Incêndios Florestais" **OR** "Wildfires" OR "Incendios Forestales" OR "Incêndios" OR "Fires" OR "Incendios"

#2 "Clima Tropical" **OR** " Tropical Climate" **OR** "Floresta Úmida" OR "Rainforest" OR "Bosque Lluvioso" **OR** "Amazon" OR "Brazil" OR "Argentina" OR "Peru" OR "Ecuador" OR "Bolivia" OR "Colombia" OR "Venezuela" OR "Guyana" OR "Suriname" OR "French Guiana" OR "Paraguay" OR "Panama" OR "El Salvador" OR "Belize" OR "Costa Rica" OR "El Salvador" OR "Guatemala" OR "Honduras" OR "Nicaragua" OR "Panama" OR "Anguilla" OR "Antigua and Barbuda" OR "Aruba" OR "Bahamas" OR "Barbados" OR "British Virgin Islands" OR "Cayman Islands" OR "Cuba" OR "Dominica" OR "Dominican Republic" OR "Grenada" OR "Guadeloupe" OR "Haiti" OR "Jamaica" OR "Martinique" OR "Montserrat" OR "Netherlands Antilles" OR "Puerto Rico" OR "Saint Barthelemy" OR "Saint Kits and Nevis" OR "Saint Lucia" OR "Saint Martin" OR "Saint Vincent and the Grenadines" OR "Trinidad and Tobago" OR "United States Virgin Islands" OR "Mexico" OR "Hawaii" OR "Cape Verde" OR "Sao Tome and Principe" OR "Gambia" OR "Senegal" OR "Guinea-Bissau" OR "Guinea" OR "Sierra Leone" OR "Liberia" OR "Ivory Coast" OR "Ghana" OR "Togo" OR "Benin" OR "Nigeria" OR "Cameroon" OR "Central African Republic" OR "South Sudan" OR "Ethiopia" OR "Equatorial Guinea" OR "Gabon" OR "Congo" OR "Democratic Republic of the Congo" OR "Uganda" OR "Rwanda" OR "Burundi" OR "Kenya" OR "Somalia" OR "Tanzania" OR "Zambia" OR "Mozambique" OR "Madagascar" OR "Seychelles" OR "Mauritius" OR "Comoros" OR "Botswana" OR "Malawi" OR "Brunei" OR "Burma" OR "Myanmar" OR "Cambodia" OR "East Timor" OR "Indonesia" OR "Laos" OR "Malaysia" OR "Philippines" OR "Singapore" OR "Thailand" OR "Vietnam" OR "India" OR "Papua New Guinea" OR "Australia" OR "Solomon Islands" OR "Vanuatu" OR "New Caledonia"

#3 "Doenças Cardiovasculares" OR "Cardiovascular Diseases" OR "Enfermedades Cardiovasculares" OR "Distúrbios Induzidos Quimicamente" OR "Chemically-Induced Disorders" OR "Trastornos Químicamente Inducidos" OR "Doenças e Anormalidades Congênitas, Hereditárias e Neonatais" OR "Congenital, Hereditary, and Neonatal Diseases and Abnormalities" OR "Enfermedades y Anomalías Neonatales Congénitas y Hereditarias" OR "Doenças do Sistema Digestório" OR "Digestive System Diseases" OR "Transtornos de Origem Ambiental" OR "Disorders of Environmental Origin" OR "Trastornos de Origen Ambiental" OR "Doenças do Sistema Endócrino" OR "Endocrine System Diseases" OR "Enfermedades del Sistema Endocrino" OR "Oftalmopatias" OR "Eye Diseases" OR "Doenças Sanguíneas e Linfáticas" OR "Hemic and Lymphatic Diseases" OR "Enfermedades Hematológicas y Linfáticas" OR "Doenças do Sistema Imunitário" OR "Immune System Diseases" OR "Enfermedades del Sistema Inmune" OR "Infecções" OR "Infections" OR "Infecciones" OR "Doenças Musculoesqueléticas" OR "Musculoskeletal Diseases" OR "Enfermedades Musculoesqueléticas" OR "Neoplasias" OR "Neoplasms" OR "Doenças do Sistema Nervoso" OR "Nervous System Diseases" OR "Enfermedades del Sistema Nervioso" OR "Doenças Nutricionais e Metabólicas" OR "Nutritional and Metabolic Diseases" OR "Enfermedades Nutricionales y Metabólicas OR Doenças Profissionais" OR "Occupational Diseases" OR "Enfermedades Profesionales" OR "Otorrinolaringopatias" OR "Otorhinolaryngologic Diseases" OR "Enfermedades Otorrinolaringológicas" OR "Condições Patológicas, Sinais e Sintomas" OR "Pathological Conditions, Signs and Symptoms" OR "Condiciones Patológicas, Signos y Síntomas" OR "Doenças Respiratórias" OR "Respiratory Tract Diseases" OR "Enfermedades Respiratorias" OR "Doenças da Pele e do Tecido Conjuntivo" OR "Skin and Connective Tissue Diseases" OR "Enfermedades de la Piel y Tejido Conjuntivo" OR "Doenças Estomatognáticas" OR "Stomatognathic Diseases" OR "Enfermedades Estomatognáticas" OR "Doenças Urogenitais" OR "Urogenital Diseases" OR

"Enfermedades Urogenitales" OR "Ferimentos e Lesões" OR "Wounds and Injuries" OR "Heridas y Lesiones" OR "Estatísticas Vitais" OR "Vital Statistics" OR "Estadísticas Vitales" OR "Assistência ao Paciente" OR "Patient Care" OR "Atención al Paciente"

Detailed search:

("Incêndios Florestais" OR "Wildfires" OR "Incendios Forestales" OR "Incêndios" OR "Fires" OR "Incendios") AND ("Clima Tropical" OR " Tropical Climate" OR "Floresta Úmida" OR "Rainforest" OR "Bosque Lluvioso" OR "Amazon" OR "Brazil" OR "Argentina" OR "Peru" OR "Ecuador" OR "Bolivia" OR "Colombia" OR "Venezuela" OR "Guyana" OR "Suriname" OR "French Guiana" OR "Paraguay" OR "Panama" OR "El Salvador" OR "Belize" OR "Costa Rica" OR "El Salvador" OR "Guatemala" OR "Honduras" OR "Nicaragua" OR "Panama" OR "Anguilla" OR "Antigua and Barbuda" OR "Aruba" OR "Bahamas" OR "Barbados" OR "British Virgin Islands" OR "Cayman Islands" OR "Cuba" OR "Dominica" OR "Dominican Republic" OR "Grenada" OR "Guadeloupe" OR "Haiti" OR "Jamaica" OR "Martinique" OR "Montserrat" OR "Netherlands Antilles" OR "Puerto Rico" OR "Saint Barthelemy" OR "Saint Kits and Nevis" OR "Saint Lucia" OR "Saint Martin" OR "Saint Vincent and the Grenadines" OR "Trinidad and Tobago" OR "United States Virgin Islands" OR "Mexico" OR "Hawaii" OR "Cape Verde" OR "Sao Tome and Principe" OR "Gambia" OR "Senegal" OR "Guinea-Bissau" OR "Guinea" OR "Sierra Leone" OR "Liberia" OR "Ivory Coast" OR "Ghana" OR "Togo" OR "Benin" OR "Nigeria" OR "Cameroon" OR "Central African Republic" OR "South Sudan" OR "Ethiopia" OR "Equatorial Guinea" OR "Gabon" OR "Congo" OR "Democratic Republic of the Congo" OR "Uganda" OR "Rwanda" OR "Burundi" OR "Kenya" OR "Somalia" OR "Tanzania" OR "Zambia" OR "Mozambique" OR "Madagascar" OR "Seychelles" OR "Mauritius" OR "Comoros" OR "Botswana" OR "Malawi" OR "Brunei" OR "Burma" OR "Myanmar" OR "Cambodia" OR "East Timor" OR "Indonesia" OR "Laos" OR "Malaysia" OR "Philippines" OR "Singapore" OR "Thailand" OR "Vietnam" OR "India" OR "Papua New Guinea" OR "Australia" OR "Solomon Islands" OR "Vanuatu" OR "New Caledonia") AND ("Doenças Cardiovasculares" OR "Cardiovascular Diseases" OR "Enfermedades Cardiovasculares" OR "Distúrbios Induzidos Quimicamente" OR "Chemically-Induced Disorders" OR "Trastornos Químicamente Inducidos" OR "Doenças e Anormalidades Congênitas, Hereditárias e Neonatais" OR "Congenital, Hereditary, and Neonatal Diseases and Abnormalities" OR "Enfermedades y Anomalías Neonatales Congénitas y Hereditarias" OR "Doenças do Sistema Digestório" OR "Digestive System Diseases" OR "Transtornos de Origem Ambiental" OR "Disorders of Environmental Origin" OR "Trastornos de Origen Ambiental" OR "Doenças do Sistema Endócrino" OR "Endocrine System Diseases" OR "Enfermedades del Sistema Endocrino" OR "Oftalmopatias" OR "Eye Diseases" OR "Doenças Sanguíneas e Linfáticas" OR "Hemic and Lymphatic Diseases" OR "Enfermedades Hematológicas y Linfáticas" OR "Doenças do Sistema Imunitário" OR "Immune System Diseases" OR "Enfermedades del Sistema Inmune" OR "Infecções" OR "Infections" OR "Infecciones" OR "Doenças Musculoesqueléticas" OR "Musculoskeletal Diseases" OR "Enfermedades Musculoesqueléticas" OR "Neoplasias" OR "Neoplasms" OR "Doenças do Sistema Nervoso" OR "Nervous System Diseases" OR "Enfermedades del Sistema Nervioso" OR "Doenças Nutricionais e Metabólicas" OR "Nutritional and Metabolic Diseases" OR "Enfermedades Nutricionales y Metabólicas OR Doenças Profissionais" OR "Occupational Diseases" OR "Enfermedades Profesionales" OR "Otorrinolaringopatias" OR "Otorhinolaryngologic Diseases" OR "Enfermedades Otorrinolaringológicas" OR "Condições Patológicas, Sinais e Sintomas" OR

"Pathological Conditions, Signs and Symptoms" OR "Condiciones Patológicas, Signos y Síntomas" OR "Doenças Respiratórias" OR "Respiratory Tract Diseases" OR "Enfermedades Respiratorias" OR "Doenças da Pele e do Tecido Conjuntivo" OR "Skin and Connective Tissue Diseases" OR "Enfermedades de la Piel y Tejido Conjuntivo" OR "Doenças Estomatognáticas" OR "Stomatognathic Diseases" OR "Enfermedades Estomatognáticas" OR "Doenças Urogenitais" OR "Urogenital Diseases" OR "Enfermedades Urogenitales" OR "Ferimentos e Lesões" OR "Wounds and Injuries" OR "Heridas y Lesiones" OR "Estatísticas Vitais" OR "Vital Statistics" OR "Estadísticas Vitales" OR "Assistência ao Paciente" OR "Patient Care" OR "Atención al Paciente")

Date of search: August 18, 2023

Results: Lilacs (n = 44), Medline (n = 246), WPRIM (n = 3), BDENF – Enfermagem (n = 2), MINASPERÚ (n = 2), Recursos Multimídia (n = 2), BINACIS (n = 1), Desastres (n = 1), MedCarib (n = 1), PAHO-IRIS (n = 1), RDSM (n = 1), Coleciona SUS (n = 1).

Embase

#4

#1 AND #2 AND #3

4,019

#3

('wildfires'/syn OR 'fire'/syn OR 'deforestation'/syn) AND [embase]/lim

42,804

#2

('tropical climate'/syn OR 'climate tropical' OR 'climates tropical' OR 'tropical climates' OR 'rainforest'/syn OR 'rainforests' OR 'rain forest'/syn OR 'forest rain' OR 'rain forests' OR 'tropical rainforest'/syn OR 'rainforest tropical'/syn OR 'rainforests tropical' OR 'tropical rainforests' OR 'amazon'/syn OR 'brazil'/syn OR 'argentina'/syn OR 'peru'/syn OR 'ecuador'/syn OR 'bolivia'/syn OR 'colombia'/syn OR 'venezuela'/syn OR 'guyana'/syn OR 'suriname'/syn OR 'french guiana'/syn OR 'paraguay'/syn OR 'belize'/syn OR 'costa rica'/syn OR 'el salvador'/syn OR 'guatemala'/syn OR 'honduras'/syn OR 'nicaragua'/syn OR 'panama'/syn OR 'anguilla'/syn OR 'antigua and barbuda'/syn OR 'aruba'/syn OR 'bahamas'/syn OR 'barbados'/syn OR 'british virgin islands'/syn OR 'cayman islands'/syn OR 'cuba'/syn OR 'dominica'/syn OR 'dominican republic'/syn OR 'grenada'/syn OR 'guadeloupe'/syn OR 'haiti'/syn OR 'jamaica'/syn OR 'martinique'/syn OR 'montserrat'/syn OR 'netherlands antilles'/syn OR 'puerto rico'/syn OR 'saint barthelemy'/syn OR 'saint kits and nevis' OR 'saint lucia'/syn OR 'saint martin'/syn OR 'saint vincent and the grenadines'/syn OR 'trinidad and tobago'/syn OR 'united states virgin islands'/syn OR 'mexico'/syn OR 'hawaii'/syn OR 'cape verde'/syn OR 'sao tome and principe'/syn OR 'gambia'/syn OR 'senegal'/syn OR 'guinea-bissau'/syn OR 'guinea'/syn OR 'sierra leone'/syn OR 'liberia'/syn OR 'ivory coast'/syn OR 'ghana'/syn OR 'togo'/syn OR 'benin'/syn OR 'nigeria'/syn

OR 'cameroon'/syn OR 'central african republic'/syn OR 'south sudan'/syn OR 'ethiopia'/syn OR 'equatorial guinea'/syn OR 'gabon'/syn OR 'congo'/syn OR 'democratic republic of the congo'/syn OR 'uganda'/syn OR 'rwanda'/syn OR 'burundi'/syn OR 'kenya'/syn OR 'somalia'/syn OR 'tanzania'/syn OR 'zambia'/syn OR 'mozambique'/syn OR 'madagascar'/syn OR 'seychelles'/syn OR 'mauritius'/syn OR 'comoros'/syn OR 'botswana'/syn OR 'malawi'/syn OR 'brunei'/syn OR 'burma'/syn OR 'myanmar'/syn OR 'cambodia'/syn OR 'east timor'/syn OR 'indonesia'/syn OR 'laos'/syn OR 'malaysia'/syn OR 'philippines'/syn OR 'singapore'/syn OR 'thailand'/syn OR 'vietnam'/syn OR 'india'/syn OR 'papua new guinea'/syn OR 'australia'/syn OR 'solomon islands'/syn OR 'vanuatu'/syn OR 'new caledonia'/syn) AND [embase]/lim

4,621,441

#1

('patient care'/syn OR 'vital statistics'/syn OR 'wounds and injuries'/syn OR 'urogenital tract disease'/syn OR 'stomatognathic diseases'/syn OR 'skin and connective tissue diseases'/syn OR 'respiratory tract diseases'/syn OR 'pathological conditions, signs and symptoms'/syn OR 'otorhinolaryngologic diseases'/syn OR 'occupational diseases'/syn OR 'nutritional and metabolic diseases'/syn OR 'nervous system diseases'/syn OR 'neoplasms'/syn OR 'musculoskeletal disease'/syn OR 'infection'/syn OR 'immune system diseases'/syn OR 'hemic and lymphatic diseases'/syn OR 'eye diseases'/syn OR 'endocrine disease'/syn OR 'environmental disease'/syn OR 'digestive system diseases'/syn OR 'congenital, hereditary, and neonatal diseases and abnormalities'/syn OR 'chemically induced disorder'/syn OR 'cardiovascular diseases'/syn) AND [embase]/lim

Date of search: August 18, 2023

Records: 4,020

EconLit

(('wildfires' or 'fire' or 'deforestation') and (((((((((('tropical climate' or 'climate tropical' or 'climates tropical' or 'tropical climates' or 'rainforest' or 'rainforests' or 'rain forest' or 'forest rain' or 'rain
forests' or 'tropical rainforest' or 'rainforest tropical' or 'rainforests tropical' or 'tropical rainforests' or 'Amazon' or 'Brazil' or 'Argentina' or 'Peru' or 'Ecuador' or 'Bolivia' or 'Colombia' or 'Venezuela' or 'Guyana' or
'Suriname' or 'French Guiana' or 'Paraguay' or 'Panama' or 'El Salvador' or 'Belize' or 'Costa Rica' or 'El Salvador' or
'Guatemala' or 'Honduras' or 'Nicaragua' or 'Panama' or 'Anguilla' or 'Antigua) and Barbuda') or 'Aruba' or 'Bahamas' or
'Barbados' or 'British Virgin Islands' or 'Cayman Islands' or 'Cuba' or 'Dominica' or 'Dominican Republic' or 'Grenada'
or 'Guadeloupe' or 'Haiti' or 'Jamaica' or 'Martinique' or 'Montserrat' or 'Netherlands Antilles' or 'Puerto Rico' or
'Saint Barthelemy' or 'Saint Kits) and Nevis') or 'Saint Lucia' or 'Saint Martin' or 'Saint Vincent) and the

Grenadines') or 'Trinidad) and Tobago') or 'United States Virgin Islands' or 'Mexico' or 'Hawaii' or 'Cape Verde' or
'Sao Tome) and Principe') or 'Gambia' or 'Senegal' or 'Guinea-Bissau' or 'Guinea' or 'Sierra Leone' or 'Liberia' or
'Ivory Coast' or 'Ghana' or 'Togo' or 'Benin' or 'Nigeria' or 'Cameroon' or 'Central African Republic' or 'South Sudan'
or 'Ethiopia' or 'Equatorial Guinea' or 'Gabon' or 'Congo' or 'Democratic Republic of the Congo' or 'Uganda' or 'Rwanda'
or 'Burundi' or 'Kenya' or 'Somalia' or 'Tanzania' or 'Zambia' or 'Mozambique' or 'Madagascar' or 'Seychelles' or
'Mauritius' or 'Comoros' or 'Botswana' or 'Malawi' or 'Brunei' or 'Burma' or 'Myanmar' or 'Cambodia' or 'East Timor' or
'Indonesia' or 'Laos' or 'Malaysia' or 'Philippines' or 'Singapore' or 'Thailand' or 'Vietnam' or 'India' or 'Papua New
Guinea' or 'Australia' or 'Solomon Islands' or 'Vanuatu' or 'New Caledonia') and (((((((((((('patient care' or 'vital
statistics' or 'wounds) and injuries') or 'urogenital tract disease' or 'stomatognathic diseases' or 'skin) and
connective tissue diseases') or 'respiratory tract diseases' or 'pathological conditions, signs) and symptoms') or
'otorhinolaryngologic diseases' or 'occupational diseases' or 'nutritional) and metabolic diseases') or 'nervous system
diseases' or 'neoplasms' or 'musculoskeletal disease' or 'infection' or 'immune system diseases' or 'hemic) and
lymphatic diseases') or 'eye diseases' or 'endocrine disease' or 'environmental disease' or 'digestive system diseases'
or 'congenital, hereditary,) and neonatal diseases and abnormalities') or 'chemically induced disorder' or
'cardiovascular diseases')).mp. [mp=tx, bt, ti, ab, ct, hw, id]

Date of search: August 18, 2023

Records: 464

Appendix II - Ongoing work

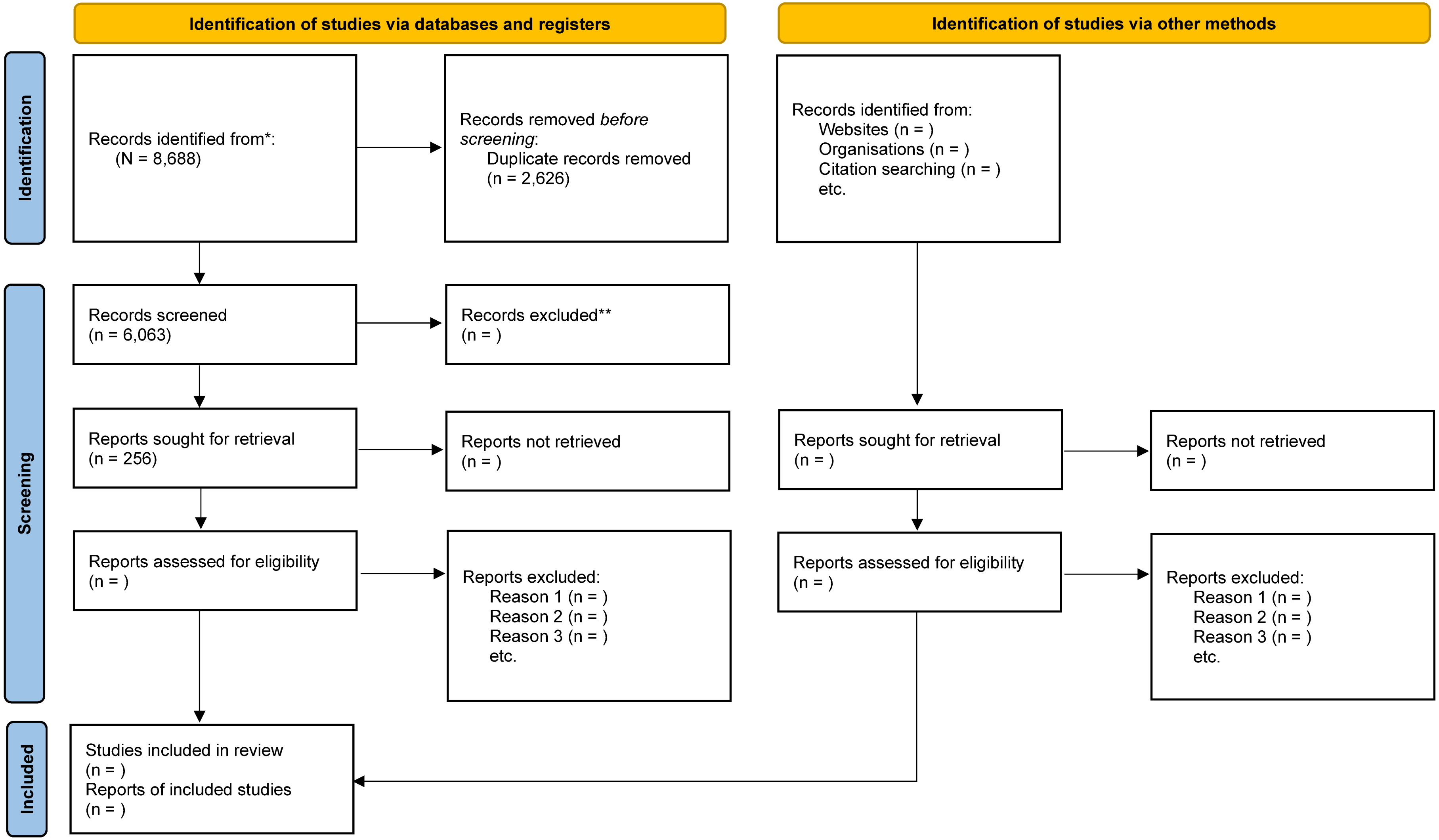

From: Page MJ, McKenzie JE, Bossuyt PM, Boutron I, Hoffmann TC, Mulrow CD, et al. The PRISMA 2020 statement: an updated guideline for reporting systematic reviews. BMJ 2021;372:n71. doi: 10.1136/bmj.n71.

Appendix III - Data extraction form

**General information**	
Reviewer who performed the data extraction:	
Date of the data extraction performed:	
Paper database:	
**Study identification**	
Publication full title:	
DOI number:	
First author name:	
Year of publication:	
Journal of publication:	
Aim of study:	
**Exposure and data source**	
Area (city, region, country):	
Exposure:	
Exposure temporal level:	
Exposure temporal duration:	
Origin of the exposure data source:	
Origin of the outcome data source:	
**Method**	
The statistical model applied:	
**Study design and findings**	
Study population:	
Health outcome:	
Type of disease:	
Lag between exposure and the health consequences	
Study findings as reported by the authors:	
Report the following list:<br>1. measure of association + standard errors for every different regression specification<br>2. Subgroups estimates<br>3. Confounders used<br>4. Identification strategy if appliable	
**Conclusions**	
Study limitations identified by the team:	
Policy recommendation from the study	
