# Supplementary material for: Wildfire, deforestation and health in tropical rainforest areas: a scoping review protocol
Source: BMJ Open. 2024 May 8;14(5):e082381. doi: 10.1136/bmjopen-2023-082381 (PMC11086528; doi:10.1136/bmjopen-2023-082381)
Supplement: Author's manuscript [file bmjopen-2023-082381.draft_revisions.pdf]

BMJ Open is committed to open peer review. As part of this commitment we make the peer review history of every article we publish publicly available.

When an article is published we post the peer reviewers' comments and the authors' responses online. We also post the versions of the paper that were used during peer review. These are the versions that the peer review comments apply to.

The versions of the paper that follow are the versions that were submitted during the peer review process. They are not the versions of record or the final published versions. They should not be cited or distributed as the published version of this manuscript.

BMJ Open is an open access journal and the full, final, typeset and author-corrected version of record of the manuscript is available on our site with no access controls, subscription charges or pay-per-view fees (http://bmjopen.bmj.com).

If you have any questions on BMJ Open's open peer review process please email info.bmjopen@bmj.com

BMJ Open

Wildfire, deforestation, and health in tropical rainforest areas: a scoping review protocol

Journal:	*BMJ Open*
Manuscript ID	bmjopen-2023-082381
Article Type:	Protocol
Date Submitted by the Author:	22-Nov-2023
Complete List of Authors:	Casais, Gustavo; Center of Data and Knowledge Integration for Health (CIDACS) - Fiocruz/BA, Center of Data and and Knowledge Integration for Health<br>Guimarães, Nathalia ; Federal University of Minas Gerais<br>Cortes, Taísa ; Center of Data and Knowledge Integration for Health (CIDACS) - Fiocruz/BA, Center of Data and and Knowledge Integration for Health<br>Pescarini , Julia ; London School of Hygiene & Tropical Medicine<br>Rebouças de Magalhães, Poliana; Center of Data and Knowledge Integration for Health (CIDACS) - Fiocruz/BA<br>Wells, Valerie; University of Glasgow<br>de Sousa Filho, José Firmino; Center of Data and Knowledge Integration for Health (CIDACS) - Fiocruz/BA<br>Delgado Neves, Danielson; Center of Data and Knowledge Integration for Health (CIDACS) - Fiocruz/BA<br>Shimonovich , Michal ; University of Glasgow<br>Olsen, Jonathan; University of Glasgow<br>de Carvalho Neto, Edgar Marcelino; Center of Data and Knowledge Integration for Health (CIDACS) - Fiocruz/BA<br>Cooper, Philip; Universidad Internacional del Ecuador; St George's, University of London<br>Katikireddi, Srinivasa; University of Glasgow<br>Emanuel, Lucas; Federal University of Bahia; Center of Data and Knowledge Integration for Health (CIDACS) - Fiocruz/BA<br>Andrade , Roberto F. S. ; Center of Data and Knowledge Integration for Health (CIDACS) - Fiocruz/BA; Federal University of Bahia<br>Ferreira dos Santos, Gervasio; Center of Data and Knowledge Integration for Health (CIDACS) - Fiocruz/BA; Federal University of Bahia<br>Barreto, Mauricio; Center of Data and Knowledge Integration for Health (CIDACS) - Fiocruz/BA; Federal University of Bahia
Keywords:	EPIDEMIOLOGY, PUBLIC HEALTH, STATISTICS & RESEARCH METHODS, TROPICAL MEDICINE

SCHOLARONE™
Manuscripts

For peer review only - http://bmjopen.bmj.com/site/about/guidelines.xhtml

Wildfire, deforestation, and health in tropical rainforest areas: a scoping review protocol

Authors

Gustavo Casais*, PhD, Center of Data and Knowledge Integration for Health (CIDACS) - Fiocruz/BA, Parque Tecnológico da Bahia, Edf. Tecnocentro, R. Mundo, 121 - sala 315 - Trobogy, Salvador - BA, Brazil, 41745-715, gustavo.casais@hotmail.com, +55 (71) 99127 3345.

Nathalia Sernizon Guimarães, PhD, Federal University of Minas Gerais, Avenida Antonio Carlos 6627, Belo Horizonte – MG, Brazil, 31270-901, nasernizon@gmail.com, +55 (31) 3409 4124.

Taísa Rodrigues Cortes, PhD, Center of Data and Knowledge Integration for Health (CIDACS) - Fiocruz/BA, Parque Tecnológico da Bahia, Edf. Tecnocentro, R. Mundo, 121 - sala 315 - Trobogy, Salvador - BA, Brazil, 41745-715, taisacortes@gmail.com, +55 (71) 99127 3345.

Julia Pescarini, PhD, London School of Hygiene and Tropical Medicine, Center of Data and and Knowledge Integration for Health (CIDACS) - Fiocruz/BA, Keppel Street, London, WC1E 7HT, United Kingdom, julia.pescarini1@lshtm.ac.uk, +44 (0)20 7636 8636.

Poliana Rebouças de Magalhães, PhD, Center of Data and Knowledge Integration for Health (CIDACS) - Fiocruz/BA, Parque Tecnológico da Bahia, Edf. Tecnocentro, R. Mundo, 121 - sala 315 - Trobogy, Salvador - BA, Brazil, 41745-715, poliana.reboucas@fiocruz.br, +55 (71) 99127 3345.

Valerie Wells, Ms, MRC/CSO Social and Public Health Sciences Unit, University of Glasgow. 90 Byres Road, Glasgow, G12 8TB, United Kingdom, Valerie.Wells@glasgow.ac.uk, +44 0141 330 4042.

José Firmino de Sousa Filho, PhD, Center of Data and Knowledge Integration for Health (CIDACS) - Fiocruz/BA, Parque Tecnológico da Bahia, Edf. Tecnocentro, R. Mundo, 121 - sala 315 - Trobogy, Salvador - BA, Brazil, 41745-715, jose.ffilho@fiocruz.br, +55 (71) 99127 3345.

Danielson Jorge Delgado Neves[1], PhD, Center of Data and Knowledge Integration for Health (CIDACS) - Fiocruz/BA, Parque Tecnológico da Bahia, Edf. Tecnocentro, R. Mundo, 121 - sala 315 - Trobogy, Salvador - BA, Brazil, 41745-715, danielson.neves@fiocruz.br, +55 (71) 99127 3345.

Michal Shimonovich, PhD, MRC/CSO Social & Public Health Sciences Unit, University of Glasgow, Berkeley Square, 99 Berkeley Street, Glasgow G3 7HR, United Kingdom, Michal.Shimonovich@glasgow.ac.uk, +44 0141 330 4042.

Jonathan R Olsen, PhD, MRC/CSO Social & Public Health Sciences Unit, University of Glasgow, Berkeley Square, 99 Berkeley Street, Glasgow G3 7HR, United Kingdom, Jonathan.Olsen@glasgow.ac.uk, +44 0141 330 4042.

Edgar Marcelino de Carvalho Neto, PhD, Center of Data and Knowledge Integration for Health (CIDACS) - Fiocruz/BA, Parque Tecnológico da Bahia, Edf. Tecnocentro, R. Mundo, 121 - sala 315 - Trobogy, Salvador - BA, Brazil, 41745-715, edgar.neto@fiocruz.br, +55 (71) 99127 3345.

For peer review only

Philip J. Cooper, PhD, St George's, University of London; School of Medicine, Universidad Internacional del Ecuador, Av. Simón Bolívar y Av. Jorge Fernández, Quito, Ecuador, pcooper@sgul.ac.uk, +593 2 2985 600.

Srinivasa Vittal Katikireddi, PhD, MRC/CSO Social & Public Health Sciences Unit, University of Glasgow, Berkeley Square, 99 Berkeley Street, Glasgow G3 7HR, United Kingdom, Vittal.Katikireddi@glasgow.ac.uk, +44 0141 330 4042.

Lucas Emanuel, Federal University of Bahia; Center of Data and Knowledge Integration for Health (CIDACS) - Fiocruz/BA, Parque Tecnológico da Bahia, Edf. Tecnocentro, R. Mundo, 121 - sala 315 - Trobogy, Salvador - BA, Brazil, 41745-715, lucasemanuel@ufba.br, +55 (71) 99127 3345.

Dr. Roberto Andrade, Federal University of Bahia; Center of Data Knowledge Integration for Health (CIDACS) - Fiocruz/BA, Parque Tecnológico da Bahia, Edf. Tecnocentro, R. Mundo, 121 - sala 315 - Trobogy, Salvador - BA, Brazil, 41745-715, randrade@ufba.br, +55 (71) 99127 3345.

Dr. Gervasio Ferreira dos Santos, Federal University of Bahia; Center of Data Knowledge Integration for Health (CIDACS) - Fiocruz/BA, Parque Tecnológico da Bahia, Edf. Tecnocentro, R. Mundo, 121 - sala 315 - Trobogy, Salvador - BA, Brazil, 41745-715, gervasios@ufba.br, +55 (71) 99127 3345.

Mauricio L. Barreto, Federal University of Bahia; Center of Data Knowledge Integration for Health (CIDACS) - Fiocruz/BA, Parque Tecnológico da Bahia, Edf. Tecnocentro, R. Mundo, 121 - sala 315 - Trobogy, Salvador - BA, Brazil, 41745-715, mauricio@ufba.br, +55 (71) 99127 3345.

*Corresponding author, Email: gustavo.casais@hotmail.com.

Word count: 1,864

Keywords: Wildfire; Deforestation; Tropical Climate; Rainforest; Health

For peer review only

Wildfire, deforestation, and health in tropical rainforest areas: a scoping review protocol

Authors

Gustavo Casais[1], Nathalia Sernizon Guimarães[2], Taísa Rodrigues Cortes[1], Julia Pescarini[1,3], Poliana Rebouças de Magalhães[1], Valerie Wells[4], José Firmino de Sousa Filho[1], Danielson Jorge Delgado Neves[1], Michal Shimonovich[4], Jonathan R Olsen[4], Edgar Marcelino de Carvalho Neto[1], Philip J. Cooper[5,6], Srinivasa Vittal Katikireddi[4], Lucas Emanuel[1,7], Roberto F. S. Andrade[1,7], Gervasio Ferreira dos Santos[1,7], Mauricio L. Barreto[1,7], on behalf of the Unit on the Social and Environmental Determinants of Health Inequalities (SEDHI)

[1]Center for Data and Knowledge Integration for Healthcare (CIDACS)

[2]Federal University of Minas Gerais

[3]London School of Hygiene and Tropical Medicine

[4]MRC/CSO Social and Public Health Sciences Unit, University of Glasgow

[5]St George's, University of London

[6]School of Medicine, Universidad Internacional del Ecuador, Quito, Ecuador

[7]Federal University of Bahia

Abstract

Introduction: Wildfires and deforestation potentially have direct effect on multiple health outcomes as well as indirect consequences for climate change. Tropical rainforest areas are characterized by high rainfall, humidity, and temperature, and they are predominantly found in low- and middle-income countries. This study will map the methods, data, and health outcomes of the scientific papers on the wildfire and deforestation in these locations.

Methods and analysis: We will carry out a scoping review according to the Joanna Briggs Institute's manual for scoping reviews and the framework proposed by Arksey and O'Malley, and Levac et al. The search for articles will be performed in 16 electronic databases using MeSH Terms and adaptations for each database. The search for local studies will be complemented by the manual search in the list of references of the studies selected to compose this review. The text screening will be done in pairs. Subsequently, we will tabulate the data by the of origin of the data source used, the methods, and the main findings on health impacts the extracted data. The results will provide descriptive statistics, along with visual representations in diagrams and tables, complemented by narrative summaries as detailed in the JBI guidelines.

Ethics and dissemination: The study does not require an ethical review due to its nature. The submission of results for publication in a peer-reviewed journal and presentation at scientific and policymakers conferences is expected.

Link to the protocol record in the Open Science Framework (OSF): https://osf.io/pnqc7/.

Strengths and limitations of this study:

For peer review only

- The proposed scoping review will be the first study to focus on assessing the wildfires and deforestation impacts on health in the tropical rainforest areas.
- The search will include several different databases, such as those from Latin America and Africa.
- It will include manuscripts in all languages with no limitations on date.
- The selected papers will be sensitive to the resolution of the tropical rainforest mapping, potentially implying improper exclusion or inclusion of studies.
- It will not conduct critical appraisal, which may result in the inclusion of studies with low relevance, reliability, validity, and applicability.

Introduction

Wildfires and deforestation have been increasingly drawing attention for their potential consequences, not only for climate change but also on the health outcomes of both local and global populations. Understanding the health effects of wildfire and deforestation on health in the low- and middle-income countries is critical for the design of evidence-based and successful mitigation plans and policies [1]. Governments across the world have been expressing this concern by adopting climate mitigation policies to contain environmental degradation. Agriculture is a significant economic sector in many of these countries, suggesting that the expansion of agricultural areas can be influenced by fluctuations in global food prices, which may lead to deforestation [2] [3]. Wildfires are directly associated with illegal forest clearing, agricultural burning of crop residue, hotter and drier conditions [4] [5] and over the past few decades, there has been an increase in the occurrence of extreme events like drought, extreme temperatures, storms, and wildfires globally. This rise is closely linked to climate change [6].

Wildfires have the potential to cause direct bodily injuries, impact housing infrastructure, and release toxic gases and particulate matter into the air [7]. Exposure to smoke from wildfires can cause acute respiratory illness and exacerbate existing disease, especially among children and elderly [8]. Additionally, the long-term effects of accumulated exposures to wildfires may be multiple including premature death, cardiovascular disease, cancer, respiratory illness, mental health, and other chronic conditions [9]. Deforestation has different causal mechanisms for public health outcomes. Deforestation can alter environmental niches, changing habitats for parasites and insects, including disease-carrying mosquitoes, which may increase the human risk of contracting vector-borne diseases such as malaria and dengue [10] [11] [12].

Although previous scoping reviews have documented potential impacts of wildfires on health outcomes [9] [13] [14] [15] [16], none have focused on rainforests in tropical regions that are located primarily in the low- and middle-income countries. Populations living in rainforest regions of the

For peer review only

Tropics include a disproportionate number of indigenous and other marginalized groups. Such populations are likely be particularly vulnerable to the adverse health effects of wildfires and deforestation given that they tend to be resource-poor, are at greater risk of premature morbidity and mortality, and have limited access to healthcare. Climate variations, including elevated temperatures, intense rainfall, and high humidity, can further impact health conditions [17]. Moreover, scoping reviews on wildfires conducted previously have certain limitations, including their focus on (i) specific population subgroups, (ii) a limited number of health outcomes, or (iii) a restricted search across only a few databases and years. The two most recent scoping reviews on health effects of deforestation were done five years ago, highlighting the need for an updated assessment [18] [19]. Conducting an updated review is crucial, especially considering the recent escalation of wildfires and deforestation. This issue is particularly significant in Brazil, where fire outbreaks have been steadily increasing, even though they have not reached the peak observed in 2004 [20] [21]. Such a review will help identify and understand the specific ways in which wildfires and deforestation affect health outcomes, hospitalizations, and vital statistics, the quantitative methods employed, and the data sources used for these analyses.

Other critical areas of investigation include geographical mapping and the analytical methods employed for analysis. There is a wide variety of information sources that provide mapping for the occurrence of wildfires and deforestation, differentiating based on geographical scale and the time period of change. Disparities in high-quality environmental data around the world highlight how some places possess better information than others, which can be considered part of the phenomenon known as the 'digital divide' [22]. Additionally, various analytical methods exist to study how wildfires and deforestation impact people's health. The outcomes of such analyses heavily rely on the modelling techniques employed, underscoring the crucial need to understand and utilize an array of available methods. This understanding is pivotal in creating thorough and accurate insights to inform the creation of future high-quality research and understand what key research gaps exist.

This scoping review aims to comprehensively map the intricate relationships between wildfires, deforestation, and their impact on health outcomes in tropical regions. Our more specific objectives will be to map: (i) the health outcomes affected by wildfires and deforestation in the tropical areas; (ii) the methods used; (iii) and the data sources related to the wildfires and deforestation. This will equip policymakers and researchers with essential information about this research area, highlighting knowledge gaps and paving the way for future research and development.

Methods and Analysis

For peer review only

This scoping review protocol follows the guidelines of the Joanna Briggs Institute. The protocol is based on the framework suggested by Peters, et al [23], Arksey and O'Malley [24], and enhanced by Levac et al. [25]. It was written according to the checklist provided by the Preferred Reporting Items for Systematic Reviews and Meta-Analyses Extension for Scoping Reviews (PRISMA-ScR) [26].

The scoping review will follow the steps below: (1) identifying the research question; (2) identifying relevant studies; (3) study selection; (4) charting the data; (5) collating, summarising, and reporting the results. The scoping review protocol was previously registered with the Open Science Framework to identify ongoing reviews and avoid unnecessary duplication of research [27].

Step 1: identifying the research question

To enhance the organization of our research question and the criteria for inclusion and exclusion we adhered to the mnemonic PCC (Population, Concept, Context), which is described with the research question in the Table 1.

According to the Oxford Dictionary, wildfire means: "a very big fire that spreads quickly and burns natural areas like woods, forests and grassland". We adopt this definition; therefore, we consider any large fire that occur in different types of vegetation which can affect urban, peri urban or rural area. The deforestation is usually associated with human activity pursuing an economic purpose (e.g., farming timber logging, expansion and infrastructure, and mining). The tropical rainforest is a warm and humid biome characterized by year-round rainfall. Renowned for its thick layers of vegetation, it consists of three distinct canopy levels located between the Tropic of Cancer and the Tropic of Capricorn [28]. We will consider the deforestation of the tropical forests located in urban, peri-urban, or rural areas.

For peer review only

Table 1 – Scoping review questions and PCC mnemonic

Question	Population (P)	Concept (C)	Context (C)

1. What are the impacts of the wildfire and deforestation on the health outcomes, hospitalization, and vital statistics in the tropical rainforest areas?<br>2. What are the methods and data sources used for this assessment?	All individuals	Mapping of the wildfire and deforestation on the health outcomes	All the areas located in the tropical rainforests

Step 2: Identifying relevant studies

Data sources

The search for scientific articles will be conducted in several databases: (1) Nursing Database (BDENF – Enfermagem), (2) National Bibliography in Argentine Health Sciences (BINACIS), (3) Coleciona SUS, (4) Desastres, (5) EconLit, (6) Embase, (7) Latin American and Caribbean Literature in Health Sciences (Lilacs), (8) Literature in Health Sciences from Caribbean countries (MedCaribe), (9) MEDLINE, (10) MEDLINE/PubMed, (11) Virtual Health Library of the Ministry of Health of Peru (MINSAPERU), (12) Literature from the Pan American Health Organization Headquarters Library (PAHO-IRIS), (13) Health Documentation Network in Mozambique (RDSM), (14) Recursos Multimídia, (15) Scopus, (16) Western Pacific Region Index Medicus (WPRIM). Access to Scopus database will be via the Capes Platform, while the databases corresponding to the previous identification number: (1), (2), (3), (4), (7), (8), (9), (11), (12), (13), (14), and (16) are available via the Virtual Health Library platform (Biblioteca Virtual da Saúde in Portuguese). In addition, references relevant to the topic will be scrutinised in the included articles.

For peer review only

Table 2 – Search terms by topics

1. 'Wildfire' OR 'Deforestation'
2. 'All categories of disease' OR 'Vital statistics' OR 'Hospitalization'
3. 'Tropical rainforest' OR 'All country names with tropical rainforest'
4. (1) AND (2) AND (3)

Note: The complete list of terms used can be found in the appendix.

Search strategy

The search strategy will be defined for each database, following the inclusion and exclusion criteria. The search terms were used according to the Medical Subject Headings (MeSH). The remaining databases were adapted by Emtree and DeCS. The expression terms were categorized into three broad aspects according to Table 2: (i) the exposure, including the wildfires and the deforestation terms, (ii) a comprehensive list of diseases, hospitalization, vital statistics terms, and (iii) tropical rainforest areas, including a list of countries with tropical rainforests [29]. The expression terms will be combined with the Boolean operators 'AND' and 'OR' in the refinement. The complete search terms in every database are in the appendix.

Step 3: Study selection

To increase the consistency of the reading of the works, a team with two reviewers will participate, in pairs, in the sequential evaluation stage of the titles, abstracts and text, independently, and then will evaluate each abstract to the inclusion criteria. In case of disagreement between the authors, it must be resolved by consensus or by the decision of one or two more authors.

The process will be registered in a flowchart of the review process according to the PRISMA-ScR [26]. All studies were exported to the Rayyan Qatar Computing Research Institute (Rayyan®), and then deduplicated by one reviewer.

Inclusion criteria

To determine and choose pertinent publications concerning the topic, the subsequent inclusion criteria will be applied: (i) quantitative studies, such as correlational, ecological, cohort, experimental, and cross-sectional studies, (ii) any individual or population groups exposed to wildfires, wildfire smoke or deforestation regardless of the exposure duration, (iii) any disease, hospitalization, or vital statistics, considered here as birth, death rate, and life expectancy, (iv) self-reported health condition.

Exclusion criteria

Studies will be excluded according to the following criteria: (i) theoretical studies; literature review (e.g., scoping review and systematic review), letter and editorials; (ii) qualitative studies (interviews, case studies etc.); (iii) environmental change only (e.g., extinction of wildlife, mosquitoes' habitats); (iv) air pollution only (e.g., air pollution from factories, mines, vehicles, without any relation to wildfires); (v) indoor fire; (vi) we will exclude studies solely focusing on health inequalities according

For peer review only

to PROGRESS Plus; and (vii) studies in which the exposed population is entirely outside the tropical area, as delimited by the Tropic of Cancer and the Tropic of Capricorn.

Step 4: Charting the data

The data of interest will be extracted with the data extraction tables and by filling out the data extraction form (in the appendix) in Microsoft Excel. During the pilot stage, two reviewers will independently conduct the task. Afterward, one reviewer will proceed, while the work will be reviewed by a second reviewer for quality assurance. The results will be categorised according to the review questions and charted in an iterative process, allowing the reviewers to continuously update these charts when additional unforeseen data are encountered. The data extraction table will be developed and tested, containing variables on the study reference (year of publication, author, journal, full title), intervention type, exposure, and data source (e.g., country, origin of the exposure data source), methods and findings (study design and modelling, health outcome, control, and treated group, point estimate, and causal identification strategy), and limitations of the study.

Step 5: Collating, summarising and reporting the results

All gathered data will be displayed in either tabular or diagrammatic formats to visually summarise the outcomes of the studies. Initially, a table containing comprehensive information about the selected papers will be provided, such as the number of studies, study design, exposure assessment (temporal and spatial scale, and data sources), statistical methods (statistical models and identification strategies), characteristics of study populations, and the countries where the studies were conducted. The data will be categorized separately for wildfires and deforestation. Different tables will be used to describe the detailed methods and data sources, followed by tables focused on findings and their subgroups. Finally, we will consider the overall implications of the results to ensure that the scoping review will provide relevant answers to the two main research questions previously posed.

Author Contributions

Gustavo Casais[123], Nathalia Sernizon Guimarães[134], Taísa Rodrigues Cortes[23], Julia Pescarini[14], Poliana Rebouças de Magalhães[23], Valerie Wells[1], José Firmino de Sousa Filho[4], Danielson Jorge Delgado Neves[4], Michal Shimonovich[34], Jonathan Olsen[4], Edgar Marcelino de Carvalho Neto[1], Philip J. Cooper[4], Srinivasa Vittal Katikireddi[4], Lucas Emanuel[4], Roberto F. S. Andrade[4], Gervasio Ferreira dos Santos[4], Mauricio L. Barreto[4].

For peer review only

[1] Data search

[2] Text Screening

[3] Data extraction

[4] Review

Patient and public involvement

There was no patient or public involvement in the design of this scoping review protocol.

Competing Interests

None declared.

Funding sources/sponsors

This research was funded by the NIHR (NIHR134801) using UK aid from the UK Government to support global health research and by the Wellcome Trust grant (226306/Z/22/Z) awarded to the CIDACS Climate and Environmental Platform (CIDACS-Clima). The views expressed in this publication are those of the author(s) and not necessarily those of the NIHR or the UK government. JO, MS, VW and SVK are employed by the MRC/CSO Social and Public Health Sciences Unit, University of Glasgow, and supported by the Medical Research Council [grant numbers MC_UU_00022/2; MC_UU_00022/4; and Chief Scientist Office [grant numbers SPHSU17; SPHSU19]. The researchers were independent of the funders; the funders had no role in the study design, data collection, analysis and interpretation of data, the decision to publish, or the preparation of the manuscript.

References

[1] S. M. Hartinger, M. Yglesias-González, L. Blanco-Villafuerte, Y. K. Palmeiro-Silva, A. G. Lescano, A. Stewart-Ibarra, ... and M. Romanello, “The 2022 South America report of The Lancet Countdown on health and climate change: trust the science. Now that we know, we must act.,” *The Lancet Regional Health–Americas,* vol. 20, 2023.

[2] D. Da Mata and M. Dotta, “Commodity booms and the environment,” SSRN 3900793, 2021.

[3] P. Dasgupta, The economics of biodiversity: the Dasgupta review, Hm Treasury, 2021.

[4] P. M. Lemieux, C. C. Lutes and D. A. Santoianni, “Emissions of organic air toxics from open burning: a comprehensive review,” *Progress in energy and combustion science,* vol. 30, no. 1, 2004.

For peer review only

[5] R. Xu, P. Yu, M. J. Abramson, F. H. Johnston, J. M. Samet, M. L. Bell, ... and Y. Guo, "Wildfires, global climate change, and human health," *New England Journal of Medicine,* vol. 383, no. 22, pp. 2173-2181, 2020.

[6] Centre for Research on the Epidemiology of Disasters, United Nations Office for Disaster Risk Reduction, "The human cost of disasters: an overview of the last 20 years (2000-2019)," 2020.

[7] C. Black and et al, "Wildfire smoke exposure and human health: Significant gaps in research for a growing public health issue," *Environmental toxicology and pharmacology,* vol. 55, pp. 186-195, 2017.

[8] R. Rocha and A. A. Sant'Anna, "Winds of fire and smoke: Air pollution and health in the Brazilian Amazon," *World Development,* vol. 151, p. 105722, 2022.

[9] E. Grant and J. D. Runkle, "Long-term health effects of wildfire exposure: a scoping review," *The Journal of Climate Change and Health,* vol. 6, p. 100110, 2022.

[10] S. C. D. C. Xavier, A. L. R. Roque, V. D. S. Lima, K. J. L. Monteiro, J. C. R. Otaviano, L. F. C. Ferreira da Silva and A. M. Jansen, "Lower richness of small wild mammal species and Chagas disease risk," *PLoS neglected tropical diseases,* vol. 6, no. 5, p. e1647.

[11] J. H. Ellwanger, B. Kulmann-Leal, V. L. Kaminski, J. Valverde-Villegas, A. B. G. VEIGA, F. R. Spilki, ... and J. A. B. Chies, "Beyond diversity loss and climate change: Impacts of Amazon deforestation on infectious diseases and public health," *Anais da Academia Brasileira de Ciências,* vol. 92.

[12] D. Lawrence and K. Vandecar, "Effects of tropical deforestation on climate and agriculture," *Nature climate change,* vol. 5, no. 1, pp. 27-36, 2015.

[13] E. Nanjappan, E. Sullo, S. Shrestha, S. Thomas and E. Nouvet, "Californian Wildfires and Associated Human Health Outcomes: An Epidemiological Scoping Review," *International Journal of Trend in Scientific Research and Development,* vol. 5, no. 5, pp. 944-953, 2021.

[14] P. To, E. Eboreime and V. I. Agyapong, "The impact of wildfires on mental health: a scoping review," *Behavioral Sciences,* vol. 11, no. 9, p. 126, 2021.

[15] C. C. Melton, C. M. Fries, R. M. Smith and L. R. Mason, "Wildfires and Older Adults: A Scoping Review of Impacts, Risks, and Interventions," *International journal of environmental research and public health,* vol. 20, no. 13, p. 6252, 2023.

[16] C. E. Reid, M. Brauer, F. H. Johnston, M. Jerrett, J. R. Balmes and C. T. Elliott, "Critical review of health impacts of wildfire smoke exposure," *Environmental health perspectives,* vol. 124, no. 9, pp. 1334-1343, 2016.

[17] R. Davis, G. R. McGregor and K. B. Enfield, "Humidity: A review and primer on atmospheric moisture and human health," *Environmental research,* vol. 144, pp. 106-116, 2016.

[18] N. D. Burkett-Cadena and A. Y. Vittor, "Deforestation and vector-borne disease: forest conversion favors important mosquito vectors of human pathogens," *Basic and applied ecology,* vol. 26, pp. 101-110, 2018.

For peer review only

[19] M. Mastel, A. Bussalleu, V. A. Paz-Soldán, G. Salmón-Mulanovich, A. Valdés-Velásquez and S. M. Hartinger, "Critical linkages between land use change and human health in the Amazon region: A scoping review," *PloS one,* vol. 13, no. 6, p. e0196414, 2018.

[20] M. Osborne, "Wildfires Reached a Five-Year High in the Brazilian Amazon," Smithsonian Magazine, 9 September 2022. [Online]. Available: https://www.smithsonianmag.com/smart-news/wildfires-reached-a-five-year-high-in-the-brazilian-amazon-180980719/. [Accessed 31 October 2023].

[21] G. Alecrim, "Número de queimadas na Amazônia em 2022 supera 2021, mas é inferior ao recorde de 2004," CNN Brasil, 20 September 2022. [Online]. Available: https://www.cnnbrasil.com.br/nacional/numero-de-queimadas-na-amazonia-em-2022-supera-2021-mas-e-inferior-ao-recorde-de-2004. [Accessed 2023 October 31].

[22] N. Kshetri, D. C. Rojas Torres, H. Besada and M. A. Moros Ochoa, "Big data as a tool to monitor and deter environmental offenders in the global south: A multiple case study," *Sustainability,* vol. 12, no. 24, p. 10436, 2020.

[23] M. D. Peters, C. M. Godfrey, H. Khalil, P. McInerney, D. Parker and C. B. Soares, "Guidance for conducting systematic scoping reviews," *JBI Evidence Implementation,* vol. 13, no. 3, pp. 141-146, 2015.

[24] H. Arksey and L. O'Malley, "Scoping studies: towards a methodological framework," *International journal of social research methodology,* vol. 8, no. 1, pp. 19-32, 2005.

[25] D. Levac, H. Colquhoun and K. K. O'Brien, "Scoping studies: advancing the methodology," *mplementation science,* vol. 5, pp. 1-9, 2010.

[26] A. C. Tricco, E. Lillie, W. Zarin, K. K. O'Brien, H. Colquhoun, D. Levac, ... and S. E. Straus, "PRISMA extension for scoping reviews (PRISMA-ScR): checklist and explanation," *Annals of internal medicine,* vol. 169, no. 7, pp. 467-473, 2018.

[27] G. Casais, N. S. Guimarães, T. R. Cortes, J. Pescarini, P. R. d. Magalhães, V. Wells, J. F. d. Sousa Filho, D. J. D. Neves, M. Shimonovich, J. R. Olsen, E. M. d. Carvalho Neto, P. J. Cooper, S. V. Katikireddi, R. Andrade, G. F. d. Santos and M. L. Barreto, "Wildfire, deforestation, and health in tropical rainforest: a scoping review protocol," 2023. [Online]. Available: https://osf.io/pnqc7/.

[28] Nasa Earth Observatory, "Rainforest," [Online]. Available: https://earthobservatory.nasa.gov/biome/biorainforest.php.

[29] D. M. Olson, E. Dinerstein, N. D. Wikramanayake, ... and K. Kassem, "Terrestrial ecoregions of the world: A new map of life on Earth," *BioScience,* vol. 51, no. 11, pp. 933-938, 2001.

[30] G. Dahlgren and M. Whitehead, "The Dahlgren-Whitehead model of health determinants: 30 years on and still chasing rainbows," *Public health,* vol. 199, pp. 20-24, 2021.

[31] Pan American Health Organization, "Social Determinants of Health," [Online]. Available: https://www.paho.org/en/topics/social-determinants-health. [Accessed 12 July 2023].

For peer review only

[32] M. Marmot, “Social determinants of health inequalities,” *The lancet,* vol. 365, no. 9464, pp. 1099-1104, 2005.

[33] B. Armstrong, F. Sera, A. M. Vicedo-Cabrera, R. Abrutzky, D. O. Åström, M. L. Bell, ... and A. Gasparrini, “The role of humidity in associations of high temperature with mortality: a multicountry, multicity study,” *Environmental health perspectives,* vol. 127, no. 9, p. 097007, 2019.

[34] J. Gao, Y. Sun, Y. Lu and L. Li, “Impact of ambient humidity on child health: a systematic review,” *PloS one,* vol. 9, no. 12, p. e112508, 2014.

For peer review only

Appendices

Appendix I – Literature search

MEDLINE/PubMed

("wildfires"[MeSH Terms] OR "wildfires"[Text Word] OR "Wildfire"[All Fields] OR "Wildland Fires"[All Fields] OR "Brush Fires"[All Fields] OR "Brush Fire"[All Fields] OR "Forest Fires"[All Fields] OR "fire forest"[All Fields] OR "fires forest"[All Fields] OR "Forest Fire"[All Fields] OR "Wild Fires"[All Fields] OR "Wild Fire"[All Fields] OR "Fires"[MeSH Terms] OR "Fires"[Text Word] OR "Fire"[All Fields] OR "fire outbreaks"[All Fields] OR "Deforestation"[All Fields] OR "Grassfire"[All Fields] OR "prescribed burn"[All Fields] OR "prescribed fire"[All Fields]) AND ("Tropical Climate"[MeSH Terms] OR "Tropical Climate"[Text Word] OR "climate tropical"[All Fields] OR "climates tropical"[All Fields] OR "Tropical Climates"[All Fields] OR "Rainforest"[MeSH Terms] OR "Rainforest"[Text Word] OR "Rainforests"[All Fields] OR "Rain Forest"[All Fields] OR "forest rain"[All Fields] OR "Rain Forests"[All Fields] OR "Tropical Rainforest"[All Fields] OR "rainforest tropical"[All Fields] OR "rainforests tropical"[All Fields] OR "Tropical Rainforests"[All Fields] OR "Amazon"[All Fields] OR "Brazil"[All Fields] OR "Argentina"[All Fields] OR "Peru"[All Fields] OR "Ecuador"[All Fields] OR "Bolivia"[All Fields] OR "Colombia"[All Fields] OR "Venezuela"[All Fields] OR "Guyana"[All Fields] OR "Suriname"[All Fields] OR "French Guiana"[All Fields] OR "Paraguay"[All Fields] OR "Panama"[All Fields] OR "El Salvador"[All Fields] OR "Belize"[All Fields] OR "Costa Rica"[All Fields] OR "El Salvador"[All Fields] OR "Guatemala"[All Fields] OR "Honduras"[All Fields] OR "Nicaragua"[All Fields] OR "Panama"[All Fields] OR "Anguilla"[All Fields] OR "Antigua and Barbuda"[All Fields] OR "Aruba"[All Fields] OR "Bahamas"[All Fields] OR "Barbados"[All Fields] OR "British Virgin Islands"[All Fields] OR "Cayman Islands"[All Fields] OR "Cuba"[All Fields] OR "Dominica"[All Fields] OR "Dominican Republic"[All Fields] OR "Grenada"[All Fields] OR "Guadeloupe"[All Fields] OR "Haiti"[All Fields] OR "Jamaica"[All Fields] OR "Martinique"[All Fields] OR "Montserrat"[All Fields] OR "Netherlands Antilles"[All Fields] OR "Puerto Rico"[All Fields] OR "Saint Barthelemy"[All Fields] OR "Saint Kits and Nevis"[All Fields] OR "Saint Lucia"[All Fields] OR "Saint Martin"[All Fields] OR "Saint Vincent and the Grenadines"[All Fields] OR "Trinidad and Tobago"[All Fields] OR "United States Virgin Islands"[All Fields] OR "Mexico"[All Fields] OR "Hawaii"[All Fields] OR "Cape Verde"[All Fields] OR "Sao Tome and Principe"[All Fields] OR "Gambia"[All Fields] OR "Senegal"[All Fields] OR "Guinea-Bissau"[All Fields] OR "Guinea"[All Fields] OR "Sierra Leone"[All Fields] OR "Liberia"[All Fields] OR "Ivory Coast"[All Fields] OR "Ghana"[All Fields] OR "Togo"[All Fields] OR "Benin"[All Fields] OR "Nigeria"[All Fields] OR "Cameroon"[All Fields] OR "Central African Republic"[All Fields] OR "South Sudan"[All Fields] OR "Ethiopia"[All Fields] OR "Equatorial Guinea"[All Fields] OR "Gabon"[All Fields] OR "Congo"[All Fields] OR "Democratic Republic of the Congo"[All Fields] OR "Uganda"[All Fields] OR "Rwanda"[All Fields] OR "Burundi"[All Fields] OR "Kenya"[All Fields] OR "Somalia"[All Fields] OR "Tanzania"[All Fields] OR "Zambia"[All Fields] OR "Mozambique"[All Fields] OR "Madagascar"[All Fields] OR "Seychelles"[All Fields] OR "Mauritius"[All Fields] OR "Comoros"[All Fields] OR "Botswana"[All Fields] OR "Malawi"[All Fields] OR "Brunei"[All Fields] OR "Burma"[All Fields] OR "Myanmar"[All Fields] OR "Cambodia"[All Fields] OR "East Timor"[All Fields] OR

For peer review only

"Indonesia"[All Fields] OR "Laos"[All Fields] OR "Malaysia"[All Fields] OR "Philippines"[All Fields] OR "Singapore"[All Fields] OR "Thailand"[All Fields] OR "Vietnam"[All Fields] OR "India"[All Fields] OR "Papua New Guinea"[All Fields] OR "Australia"[All Fields] OR "Solomon Islands"[All Fields] OR "Vanuatu"[All Fields] OR "New Caledonia"[All Fields]) AND ("cardiovascular diseases"[MeSH Terms] OR "cardiovascular diseases"[Text Word] OR "Cardiovascular Disease"[All Fields] OR "disease cardiovascular"[All Fields] OR "Major Adverse Cardiac Events"[All Fields] OR "Cardiac Events"[All Fields] OR "Cardiac Event"[All Fields] OR "event cardiac"[All Fields] OR "Adverse Cardiac Event"[All Fields] OR "Adverse Cardiac Events"[All Fields] OR "cardiac events adverse"[All Fields] OR "chemically induced disorders"[MeSH Terms] OR "chemically induced disorders"[Text Word] OR "chemically induced disorders"[All Fields] OR "Chemically-Induced Disorder"[All Fields] OR "congenital, hereditary, and neonatal diseases and abnormalities"[MeSH Terms] OR "congenital hereditary and neonatal diseases and abnormalities"[Text Word] OR "Congenital Disorders"[All Fields] OR "disorder congenital"[All Fields] OR "disorders congenital"[All Fields] OR "Neonatal Diseases and Abnormalities"[All Fields] OR "Digestive System Diseases"[MeSH Terms] OR "Digestive System Diseases"[Text Word] OR "Digestive System Disease"[All Fields] OR "Digestive System Disorders"[All Fields] OR "Digestive System Disorder"[All Fields] OR "system disorders digestive"[All Fields] OR "Hepatobiliary Disorders"[All Fields] OR "Hepatobiliary Disorder"[All Fields] OR "Hepatobiliary Diseases"[All Fields] OR "Hepatobiliary Disease"[All Fields] OR "Disorders of Environmental Origin"[MeSH Terms] OR "Disorders of Environmental Origin"[Text Word] OR "Endocrine System Diseases"[MeSH Terms] OR "Endocrine System Diseases"[Text Word] OR "disease endocrine system"[All Fields] OR "diseases endocrine system"[All Fields] OR "Endocrine System Disease"[All Fields] OR "system disease endocrine"[All Fields] OR "system diseases endocrine"[All Fields] OR "Endocrine Diseases"[All Fields] OR "disease endocrine"[All Fields] OR "diseases endocrine"[All Fields] OR "Endocrine Disease"[All Fields] OR "Diseases of Endocrine System"[All Fields] OR "Eye Diseases"[MeSH Terms] OR "Eye Diseases"[Text Word] OR "Eye Disease"[All Fields] OR "Eye Disorders"[All Fields] OR "Eye Disorder"[All Fields] OR "Hemic and Lymphatic Diseases"[MeSH Terms] OR "Hemic and Lymphatic Diseases"[Text Word] OR "Blood and Lymphatic System Disorders"[All Fields] OR "Immune System Diseases"[MeSH Terms] OR "Immune System Diseases"[Text Word] OR "disease immune system"[All Fields] OR "Immune System Disease"[All Fields] OR "Immunologic Diseases"[All Fields] OR "disease immunologic"[All Fields] OR "Immunologic Disease"[All Fields] OR "Immunological Diseases"[All Fields] OR "disease immunological"[All Fields] OR "Immunological Disease"[All Fields] OR "Immune Diseases"[All Fields] OR "disease immune"[All Fields] OR "Immune Disease"[All Fields] OR "Diseases of Immune System"[All Fields] OR "Immune Disorders"[All Fields] OR "Immune Disorder"[All Fields] OR "Immune System Disorders"[All Fields] OR "disorder immune system"[All Fields] OR "Immune System Disorder"[All Fields] OR "Infections"[MeSH Terms] OR "Infections"[Text Word] OR "Infection and Infestation"[All Fields] OR "Infestation and Infection"[All Fields] OR "Infections and Infestations"[All Fields] OR "Infestations and Infections"[All Fields] OR "Infection"[All Fields] OR "Musculoskeletal Diseases"[MeSH Terms] OR "Musculoskeletal Diseases"[Text Word] OR "Musculoskeletal Disease"[All Fields] OR "Orthopedic Disorders"[All Fields] OR "Orthopedic Disorder"[All Fields] OR "Neoplasms"[MeSH Terms] OR "Neoplasms"[Text Word] OR "Tumor"[All Fields] OR "Neoplasm"[All Fields] OR "Tumors"[All Fields] OR "Neoplasia"[All Fields] OR "Neoplasias"[All Fields] OR "Cancer"[All Fields] OR "Cancers"[All Fields] OR "Malignant Neoplasm"[All Fields] OR "Malignancy"[All Fields] OR "Malignancies"[All Fields] OR "Malignant Neoplasms"[All Fields] OR "neoplasm malignant"[All Fields] OR "neoplasms malignant"[All Fields] OR "Benign

For peer review only

Neoplasms"[All Fields] OR "Benign Neoplasm"[All Fields] OR "neoplasms benign"[All Fields] OR "neoplasm benign"[All Fields] OR "Nervous System Diseases"[MeSH Terms] OR "Nervous System Diseases"[Text Word] OR "disease nervous system"[All Fields] OR "diseases nervous system"[All Fields] OR "Nervous System Disease"[All Fields] OR "Neurologic Disorders"[All Fields] OR "disorder neurologic"[All Fields] OR "disorders neurologic"[All Fields] OR "Neurologic Disorder"[All Fields] OR "Neurological Disorders"[All Fields] OR "disorder neurological"[All Fields] OR "disorders neurological"[All Fields] OR "Neurological Disorder"[All Fields] OR "Nervous System Disorders"[All Fields] OR "disorder nervous system"[All Fields] OR "disorders nervous system"[All Fields] OR "Nervous System Disorder"[All Fields] OR "Nutritional and Metabolic Diseases"[MeSH Terms] OR "Nutritional and Metabolic Diseases"[Text Word] OR "Occupational Diseases"[MeSH Terms] OR "Occupational Diseases"[Text Word] OR "disease occupational"[All Fields] OR "Occupational Disease"[All Fields] OR "Occupational Illnesses"[All Fields] OR "illnesses occupational"[All Fields] OR "diseases occupational"[All Fields] OR "Otorhinolaryngologic Diseases"[MeSH Terms] OR "Otorhinolaryngologic Diseases"[Text Word] OR "Otorhinolaryngological Disease"[All Fields] OR "Otorhinolaryngological Diseases"[All Fields] OR "Otolaryngological Diseases"[All Fields] OR "diseases otolaryngological"[All Fields] OR "Otolaryngological Disease"[All Fields] OR "Otorhinolaryngologic Disease"[All Fields] OR "ENT Diseases"[All Fields] OR "disease ent"[All Fields] OR "diseases ent"[All Fields] OR "ENT Disease"[All Fields] OR "Otolaryngologic Diseases"[All Fields] OR "disease otolaryngologic"[All Fields] OR "diseases otolaryngologic"[All Fields] OR "Otolaryngologic Disease"[All Fields] OR "pathological conditions, signs and symptoms"[MeSH Terms] OR "pathological conditions signs and symptoms"[Text Word] OR "Respiratory Tract Diseases"[MeSH Terms] OR "Respiratory Tract Diseases"[Text Word] OR "disease respiratory tract"[All Fields] OR "Respiratory Tract Disease"[All Fields] OR "Respiratory Diseases"[All Fields] OR "Respiratory System Diseases"[All Fields] OR "disease respiratory system"[All Fields] OR "Respiratory System Disease"[All Fields] OR "Skin and Connective Tissue Diseases"[MeSH Terms] OR "Skin and Connective Tissue Diseases"[Text Word] OR "Stomatognathic Diseases"[MeSH Terms] OR "Stomatognathic Diseases"[Text Word] OR "Stomatognathic Disease"[All Fields] OR "Mouth and Tooth Diseases"[All Fields] OR "Dental Diseases"[All Fields] OR "Dental Disease"[All Fields] OR "disease dental"[All Fields] OR "diseases dental"[All Fields] OR "Urogenital Diseases"[MeSH Terms] OR "Urogenital Diseases"[Text Word] OR "disease urogenital"[All Fields] OR "Urogenital Disease"[All Fields] OR "Genitourinary Diseases"[All Fields] OR "disease genitourinary"[All Fields] OR "Genitourinary Disease"[All Fields] OR "Wounds and Injuries"[MeSH Terms] OR "Wounds and Injuries"[Text Word] OR "Injuries and Wounds"[All Fields] OR "Wounds and Injury"[All Fields] OR "Injury and Wounds"[All Fields] OR "wounds injury"[All Fields] OR "Trauma"[All Fields] OR "Traumas"[All Fields] OR "injuries wounds"[All Fields] OR "research related injuries"[All Fields] OR "research related injuries"[All Fields] OR "Research-Related Injury"[All Fields] OR "Injuries"[All Fields] OR "Injury"[All Fields] OR "Wounds"[All Fields] OR "Wound"[All Fields] OR "Vital Statistics"[MeSH Terms] OR "Vital Statistics"[Text Word] OR "statistics vital"[All Fields] OR "Vital Statistics Registration"[All Fields] OR "Registration of Vital Statistics"[All Fields] OR "Vital Statistics Registrations"[All Fields] OR "registration vital statistics"[All Fields] OR "registrations vital statistics"[All Fields] OR "Patient Care"[MeSH Terms] OR "Patient Care"[Text Word] OR "care patient"[All Fields] OR "Informal care"[All Fields] OR "Informal cares"[All Fields] OR "care informal"[All Fields])

Date of search: August 18, 2023

For peer review only

Results: 2,296 [4]

Scopus

Search: ("wildfires" OR "wildfire" OR "wildland fires" OR "brush fires" OR "brush fire" OR "forest fires" OR "fire forest" OR "fires forest" OR "forest fire" OR "wild fires" OR "wild fire" OR "fires" OR "fire" OR "fire outbreaks" OR "deforestation" OR "grassfire" OR "prescribed burn" OR "prescribed fire")AND ("tropical climate" OR "climate tropical" OR "climates tropical" OR "tropical climates" OR "rainforest" OR "rainforests" OR "rain forest" OR "forest rain" OR "rain forests" OR "tropical rainforest" OR "rainforest tropical" OR "rainforests tropical" OR "tropical rainforests" OR "Amazon" OR "Brazil" OR "Argentina" OR "Peru" OR "Ecuador" OR "Bolivia" OR "Colombia" OR "Venezuela" OR "Guyana" OR "Suriname" OR "French Guiana" OR "Paraguay" OR "Panama" OR "El Salvador" OR "Belize" OR "Costa Rica" OR "El Salvador" OR "Guatemala" OR "Honduras" OR "Nicaragua" OR "Panama" OR "Anguilla" OR "Antigua and Barbuda" OR "Aruba" OR "Bahamas" OR "Barbados" OR "British Virgin Islands" OR "Cayman Islands" OR "Cuba" OR "Dominica" OR "Dominican Republic" OR "Grenada" OR "Guadeloupe" OR "Haiti" OR "Jamaica" OR "Martinique" OR "Montserrat" OR "Netherlands Antilles" OR "Puerto Rico" OR "Saint Barthelemy" OR "Saint Kits and Nevis" OR "Saint Lucia" OR "Saint Martin" OR "Saint Vincent and the Grenadines" OR "Trinidad and Tobago" OR "United States Virgin Islands" OR "Mexico" OR "Hawaii" OR "Cape Verde" OR "Sao Tome and Principe" OR "Gambia" OR "Senegal" OR "Guinea-Bissau" OR "Guinea" OR "Sierra Leone" OR "Liberia" OR "Ivory Coast" OR "Ghana" OR "Togo" OR "Benin" OR "Nigeria" OR "Cameroon" OR "Central African Republic" OR "South Sudan" OR "Ethiopia" OR "Equatorial Guinea" OR "Gabon" OR "Congo" OR "Democratic Republic of the Congo" OR "Uganda" OR "Rwanda" OR "Burundi" OR "Kenya" OR "Somalia" OR "Tanzania" OR "Zambia" OR "Mozambique" OR "Madagascar" OR "Seychelles" OR "Mauritius" OR "Comoros" OR "Botswana" OR "Malawi" OR "Brunei" OR "Burma" OR "Myanmar" OR "Cambodia" OR "East Timor" OR "Indonesia" OR "Laos" OR "Malaysia" OR "Philippines" OR "Singapore" OR "Thailand" OR "Vietnam" OR "India" OR "Papua New Guinea" OR "Australia" OR "Solomon Islands" OR "Vanuatu" OR "New Caledonia") AND ("cardiovascular diseases" OR "Cardiovascular Disease" OR "disease cardiovascular" OR "Major Adverse Cardiac Events" OR "Cardiac Events" OR "Cardiac Event" OR "event cardiac" OR "Adverse Cardiac Event" OR "Adverse Cardiac Events" OR "cardiac events adverse" OR "chemically induced disorders" OR "chemically induced disorders" OR "Chemically-Induced Disorder" OR "congenital, hereditary, and neonatal diseases and abnormalities" OR "Congenital Disorders" OR "disorder congenital" OR "disorders congenital" OR "Neonatal Diseases and Abnormalities" OR "Digestive System Diseases" OR "Digestive System Disease" OR "Digestive System Disorders" OR "Digestive System Disorder" OR "system disorders digestive" OR "Hepatobiliary Disorders" OR "Hepatobiliary Disorder" OR "Hepatobiliary Diseases" OR "Hepatobiliary Disease" OR "Disorders of Environmental Origin"OR "Endocrine System Diseases" OR "disease endocrine system" OR "diseases endocrine system" OR "Endocrine System Disease" OR "system disease endocrine" OR "system diseases endocrine" OR "Endocrine Diseases" OR "disease endocrine" OR "diseases endocrine" OR "Endocrine Disease" OR "Diseases of Endocrine System" OR "Eye Diseases" OR "Eye Disease" OR "Eye Disorders" OR "Eye Disorder" OR "Hemic and Lymphatic Diseases" OR "Blood and Lymphatic System Disorders" OR "Immune System Diseases" OR "disease immune system" OR "Immune System Disease" OR "Immunologic Diseases" OR "disease immunologic" OR "Immunologic Disease" OR "Immunological Diseases"

For peer review only

OR "disease immunological" OR "Immunological Disease" OR "Immune Diseases" OR "disease immune" OR "Immune Disease" OR "Diseases of Immune System" OR "Immune Disorders" OR "Immune Disorder" OR "Immune System Disorders" OR "disorder immune system" OR "Immune System Disorder" OR "Infections" OR "Infection and Infestation" OR "Infestation and Infection" OR "Infections and Infestations" OR "Infestations and Infections" OR "Infection" OR "Musculoskeletal Diseases" OR "Musculoskeletal Disease" OR "Orthopedic Disorders" OR "Orthopedic Disorder" OR "Neoplasms" OR "Tumor" OR "Neoplasm" OR "Tumors" OR "Neoplasia" OR "Neoplasias" OR "Cancer" OR "Cancers" OR "Malignant Neoplasm" OR "Malignancy" OR "Malignancies" OR "Malignant Neoplasms" OR "neoplasm malignant" OR "neoplasms malignant" OR "Benign Neoplasms" OR "Benign Neoplasm" OR "neoplasms benign" OR "neoplasm benign" OR "Nervous System Diseases" OR "disease nervous system" OR "diseases nervous system" OR "Nervous System Disease" OR "Neurologic Disorders" OR "disorder neurologic" OR "disorders neurologic" OR "Neurologic Disorder" OR "Neurological Disorders" OR "disorder neurological" OR "disorders neurological" OR "Neurological Disorder" OR "Nervous System Disorders" OR "disorder nervous system" OR "disorders nervous system" OR "Nervous System Disorder" OR "Nutritional and Metabolic Diseases" OR "Occupational Diseases" OR "disease occupational" OR "Occupational Disease" OR "Occupational Illnesses" OR "illnesses occupational" OR "diseases occupational" OR "Otorhinolaryngologic Diseases" OR "Otorhinolaryngological Disease" OR "Otorhinolaryngological Diseases" OR "Otolaryngological Diseases" OR "diseases otolaryngological" OR "Otolaryngological Disease" OR "Otorhinolaryngologic Disease" OR "ENT Diseases" OR "disease ent" OR "diseases ent" OR "ENT Disease" OR "Otolaryngologic Diseases" OR "disease otolaryngologic" OR "diseases otolaryngologic" OR "Otolaryngologic Disease" OR "pathological conditions, signs and symptoms" OR "Respiratory Tract Diseases" OR "disease respiratory tract" OR "Respiratory Tract Disease" OR "Respiratory Diseases" OR "Respiratory System Diseases" OR "disease respiratory system" OR "Respiratory System Disease" OR "Skin and Connective Tissue Diseases" OR "Stomatognathic Diseases" OR "Stomatognathic Disease" OR "Mouth and Tooth Diseases" OR "Dental Diseases" OR "Dental Disease" OR "disease dental" OR "diseases dental" OR "Urogenital Diseases" OR "disease urogenital" OR "Urogenital Disease" OR "Genitourinary Diseases" OR "disease genitourinary" OR "Genitourinary Disease" OR "Wounds and Injuries" OR "Injuries and Wounds" OR "Wounds and Injury" OR "Injury and Wounds" OR "wounds injury" OR "Trauma" OR "Traumas" OR "injuries wounds" OR "research related injuries" OR "research related injuries" OR "Research-Related Injury" OR "Injuries" OR "Injury" OR "Wounds" OR "Wound" OR "Vital Statistics" OR "statistics vital" OR "Vital Statistics Registration" OR "Registration of Vital Statistics" OR "Vital Statistics Registrations" OR "registration vital statistics" OR "registrations vital statistics" OR "Patient Care" OR "care patient" OR "Informal care" OR "Informal cares" OR "care informal")

Date of search: August 18, 2023

Results: 1,604

Biblioteca Virtual em Saúde

For peer review only

#1 "Incêndios Florestais" **OR** "Wildfires" OR "Incendios Forestales" OR "Incêndios" OR "Fires" OR "Incendios"

#2 "Clima Tropical" **OR** " Tropical Climate" **OR** "Floresta Úmida" OR "Rainforest" OR "Bosque Lluvioso" **OR** "Amazon" OR "Brazil" OR "Argentina" OR "Peru" OR "Ecuador" OR "Bolivia" OR "Colombia" OR "Venezuela" OR "Guyana" OR "Suriname" OR "French Guiana" OR "Paraguay" OR "Panama" OR "El Salvador" OR "Belize" OR "Costa Rica" OR "El Salvador" OR "Guatemala" OR "Honduras" OR "Nicaragua" OR "Panama" OR "Anguilla" OR "Antigua and Barbuda" OR "Aruba" OR "Bahamas" OR "Barbados" OR "British Virgin Islands" OR "Cayman Islands" OR "Cuba" OR "Dominica" OR "Dominican Republic" OR "Grenada" OR "Guadeloupe" OR "Haiti" OR "Jamaica" OR "Martinique" OR "Montserrat" OR "Netherlands Antilles" OR "Puerto Rico" OR "Saint Barthelemy" OR "Saint Kits and Nevis" OR "Saint Lucia" OR "Saint Martin" OR "Saint Vincent and the Grenadines" OR "Trinidad and Tobago" OR "United States Virgin Islands" OR "Mexico" OR "Hawaii" OR "Cape Verde" OR "Sao Tome and Principe" OR "Gambia" OR "Senegal" OR "Guinea-Bissau" OR "Guinea" OR "Sierra Leone" OR "Liberia" OR "Ivory Coast" OR "Ghana" OR "Togo" OR "Benin" OR "Nigeria" OR "Cameroon" OR "Central African Republic" OR "South Sudan" OR "Ethiopia" OR "Equatorial Guinea" OR "Gabon" OR "Congo" OR "Democratic Republic of the Congo" OR "Uganda" OR "Rwanda" OR "Burundi" OR "Kenya" OR "Somalia" OR "Tanzania" OR "Zambia" OR "Mozambique" OR "Madagascar" OR "Seychelles" OR "Mauritius" OR "Comoros" OR "Botswana" OR "Malawi" OR "Brunei" OR "Burma" OR "Myanmar" OR "Cambodia" OR "East Timor" OR "Indonesia" OR "Laos" OR "Malaysia" OR "Philippines" OR "Singapore" OR "Thailand" OR "Vietnam" OR "India" OR "Papua New Guinea" OR "Australia" OR "Solomon Islands" OR "Vanuatu" OR "New Caledonia"

#3 "Doenças Cardiovasculares" OR "Cardiovascular Diseases" OR "Enfermedades Cardiovasculares" OR "Distúrbios Induzidos Quimicamente" OR "Chemically-Induced Disorders" OR "Trastornos Químicamente Inducidos" OR "Doenças e Anormalidades Congênitas, Hereditárias e Neonatais" OR "Congenital, Hereditary, and Neonatal Diseases and Abnormalities" OR "Enfermedades y Anomalías Neonatales Congénitas y Hereditarias" OR "Doenças do Sistema Digestório" OR "Digestive System Diseases" OR "Transtornos de Origem Ambiental" OR "Disorders of Environmental Origin" OR "Trastornos de Origen Ambiental" OR "Doenças do Sistema Endócrino" OR "Endocrine System Diseases" OR "Enfermedades del Sistema Endocrino" OR "Oftalmopatias" OR "Eye Diseases" OR "Doenças Sanguíneas e Linfáticas" OR "Hemic and Lymphatic Diseases" OR "Enfermedades Hematológicas y Linfáticas" OR "Doenças do Sistema Imunitário" OR "Immune System Diseases" OR "Enfermedades del Sistema Inmune" OR "Infecções" OR "Infections" OR "Infecciones" OR "Doenças Musculoesqueléticas" OR "Musculoskeletal Diseases" OR "Enfermedades Musculoesqueléticas" OR "Neoplasias" OR "Neoplasms" OR "Doenças do Sistema Nervoso" OR "Nervous System Diseases" OR "Enfermedades del Sistema Nervioso" OR "Doenças Nutricionais e Metabólicas" OR "Nutritional and Metabolic Diseases" OR "Enfermedades Nutricionales y Metabólicas OR Doenças Profissionais" OR "Occupational Diseases" OR "Enfermedades Profesionales" OR "Otorrinolaringopatias" OR "Otorhinolaryngologic Diseases" OR "Enfermedades Otorrinolaringológicas" OR "Condições Patológicas, Sinais e Sintomas" OR "Pathological Conditions, Signs and Symptoms" OR "Condiciones Patológicas, Signos y Síntomas" OR "Doenças Respiratórias" OR "Respiratory Tract Diseases" OR "Enfermedades Respiratorias" OR "Doenças da Pele e do Tecido Conjuntivo" OR "Skin and Connective Tissue Diseases" OR "Enfermedades de la Piel y Tejido Conjuntivo" OR "Doenças Estomatognáticas" OR "Stomatognathic Diseases" OR "Enfermedades Estomatognáticas" OR "Doenças Urogenitais" OR "Urogenital Diseases" OR

"Enfermedades Urogenitales" OR "Ferimentos e Lesões" OR "Wounds and Injuries" OR "Heridas y Lesiones" OR "Estatísticas Vitais" OR "Vital Statistics" OR "Estadísticas Vitales" OR "Assistência ao Paciente" OR "Patient Care" OR "Atención al Paciente"

Detailed search:

("Incêndios Florestais" OR "Wildfires" OR "Incendios Forestales" OR "Incêndios" OR "Fires" OR "Incendios") AND ("Clima Tropical" OR " Tropical Climate" OR "Floresta Úmida" OR "Rainforest" OR "Bosque Lluvioso" OR "Amazon" OR "Brazil" OR "Argentina" OR "Peru" OR "Ecuador" OR "Bolivia" OR "Colombia" OR "Venezuela" OR "Guyana" OR "Suriname" OR "French Guiana" OR "Paraguay" OR "Panama" OR "El Salvador" OR "Belize" OR "Costa Rica" OR "El Salvador" OR "Guatemala" OR "Honduras" OR "Nicaragua" OR "Panama" OR "Anguilla" OR "Antigua and Barbuda" OR "Aruba" OR "Bahamas" OR "Barbados" OR "British Virgin Islands" OR "Cayman Islands" OR "Cuba" OR "Dominica" OR "Dominican Republic" OR "Grenada" OR "Guadeloupe" OR "Haiti" OR "Jamaica" OR "Martinique" OR "Montserrat" OR "Netherlands Antilles" OR "Puerto Rico" OR "Saint Barthelemy" OR "Saint Kits and Nevis" OR "Saint Lucia" OR "Saint Martin" OR "Saint Vincent and the Grenadines" OR "Trinidad and Tobago" OR "United States Virgin Islands" OR "Mexico" OR "Hawaii" OR "Cape Verde" OR "Sao Tome and Principe" OR "Gambia" OR "Senegal" OR "Guinea-Bissau" OR "Guinea" OR "Sierra Leone" OR "Liberia" OR "Ivory Coast" OR "Ghana" OR "Togo" OR "Benin" OR "Nigeria" OR "Cameroon" OR "Central African Republic" OR "South Sudan" OR "Ethiopia" OR "Equatorial Guinea" OR "Gabon" OR "Congo" OR "Democratic Republic of the Congo" OR "Uganda" OR "Rwanda" OR "Burundi" OR "Kenya" OR "Somalia" OR "Tanzania" OR "Zambia" OR "Mozambique" OR "Madagascar" OR "Seychelles" OR "Mauritius" OR "Comoros" OR "Botswana" OR "Malawi" OR "Brunei" OR "Burma" OR "Myanmar" OR "Cambodia" OR "East Timor" OR "Indonesia" OR "Laos" OR "Malaysia" OR "Philippines" OR "Singapore" OR "Thailand" OR "Vietnam" OR "India" OR "Papua New Guinea" OR "Australia" OR "Solomon Islands" OR "Vanuatu" OR "New Caledonia") AND ("Doenças Cardiovasculares" OR "Cardiovascular Diseases" OR "Enfermedades Cardiovasculares" OR "Distúrbios Induzidos Quimicamente" OR "Chemically-Induced Disorders" OR "Trastornos Químicamente Inducidos" OR "Doenças e Anormalidades Congênitas, Hereditárias e Neonatais" OR "Congenital, Hereditary, and Neonatal Diseases and Abnormalities" OR "Enfermedades y Anomalías Neonatales Congénitas y Hereditarias" OR "Doenças do Sistema Digestório" OR "Digestive System Diseases" OR "Transtornos de Origem Ambiental" OR "Disorders of Environmental Origin" OR "Trastornos de Origen Ambiental" OR "Doenças do Sistema Endócrino" OR "Endocrine System Diseases" OR "Enfermedades del Sistema Endocrino" OR "Oftalmopatias" OR "Eye Diseases" OR "Doenças Sanguíneas e Linfáticas" OR "Hemic and Lymphatic Diseases" OR "Enfermedades Hematológicas y Linfáticas" OR "Doenças do Sistema Imunitário" OR "Immune System Diseases" OR "Enfermedades del Sistema Inmune" OR "Infecções" OR "Infections" OR "Infecciones" OR "Doenças Musculoesqueléticas" OR "Musculoskeletal Diseases" OR "Enfermedades Musculoesqueléticas" OR "Neoplasias" OR "Neoplasms" OR "Doenças do Sistema Nervoso" OR "Nervous System Diseases" OR "Enfermedades del Sistema Nervioso" OR "Doenças Nutricionais e Metabólicas" OR "Nutritional and Metabolic Diseases" OR "Enfermedades Nutricionales y Metabólicas OR Doenças Profissionais" OR "Occupational Diseases" OR "Enfermedades Profesionales" OR "Otorrinolaringopatias" OR "Otorhinolaryngologic Diseases" OR "Enfermedades Otorrinolaringológicas" OR "Condições Patológicas, Sinais e Sintomas" OR

"Pathological Conditions, Signs and Symptoms" OR "Condiciones Patológicas, Signos y Síntomas" OR "Doenças Respiratórias" OR "Respiratory Tract Diseases" OR "Enfermedades Respiratorias" OR "Doenças da Pele e do Tecido Conjuntivo" OR "Skin and Connective Tissue Diseases" OR "Enfermedades de la Piel y Tejido Conjuntivo" OR "Doenças Estomatognáticas" OR "Stomatognathic Diseases" OR "Enfermedades Estomatognáticas" OR "Doenças Urogenitais" OR "Urogenital Diseases" OR "Enfermedades Urogenitales" OR "Ferimentos e Lesões" OR "Wounds and Injuries" OR "Heridas y Lesiones" OR "Estatísticas Vitais" OR "Vital Statistics" OR "Estadísticas Vitales" OR "Assistência ao Paciente" OR "Patient Care" OR "Atención al Paciente")

Date of search: August 18, 2023

Results: Lilacs (n = 44), Medline (n = 246), WPRIM (n = 3), BDENF – Enfermagem (n = 2), MINASPERÚ (n = 2), Recursos Multimídia (n = 2), BINACIS (n = 1), Desastres (n = 1), MedCarib (n = 1), PAHO-IRIS (n = 1), RDSM (n = 1), Coleciona SUS (n = 1).

Embase

#4

#1 AND #2 AND #3

4,019

#3

('wildfires'/syn OR 'fire'/syn OR 'deforestation'/syn) AND [embase]/lim

42,804

#2

('tropical climate'/syn OR 'climate tropical' OR 'climates tropical' OR 'tropical climates' OR 'rainforest'/syn OR 'rainforests' OR 'rain forest'/syn OR 'forest rain' OR 'rain forests' OR 'tropical rainforest'/syn OR 'rainforest tropical'/syn OR 'rainforests tropical' OR 'tropical rainforests' OR 'amazon'/syn OR 'brazil'/syn OR 'argentina'/syn OR 'peru'/syn OR 'ecuador'/syn OR 'bolivia'/syn OR 'colombia'/syn OR 'venezuela'/syn OR 'guyana'/syn OR 'suriname'/syn OR 'french guiana'/syn OR 'paraguay'/syn OR 'belize'/syn OR 'costa rica'/syn OR 'el salvador'/syn OR 'guatemala'/syn OR 'honduras'/syn OR 'nicaragua'/syn OR 'panama'/syn OR 'anguilla'/syn OR 'antigua and barbuda'/syn OR 'aruba'/syn OR 'bahamas'/syn OR 'barbados'/syn OR 'british virgin islands'/syn OR 'cayman islands'/syn OR 'cuba'/syn OR 'dominica'/syn OR 'dominican republic'/syn OR 'grenada'/syn OR 'guadeloupe'/syn OR 'haiti'/syn OR 'jamaica'/syn OR 'martinique'/syn OR 'montserrat'/syn OR 'netherlands antilles'/syn OR 'puerto rico'/syn OR 'saint barthelemy'/syn OR 'saint kits and nevis' OR 'saint lucia'/syn OR 'saint martin'/syn OR 'saint vincent and the grenadines'/syn OR 'trinidad and tobago'/syn OR 'united states virgin islands'/syn OR 'mexico'/syn OR 'hawaii'/syn OR 'cape verde'/syn OR 'sao tome and principe'/syn OR 'gambia'/syn OR 'senegal'/syn OR 'guinea-bissau'/syn OR 'guinea'/syn OR 'sierra leone'/syn OR 'liberia'/syn OR 'ivory coast'/syn OR 'ghana'/syn OR 'togo'/syn OR 'benin'/syn OR 'nigeria'/syn

OR 'cameroon'/syn OR 'central african republic'/syn OR 'south sudan'/syn OR 'ethiopia'/syn OR 'equatorial guinea'/syn OR 'gabon'/syn OR 'congo'/syn OR 'democratic republic of the congo'/syn OR 'uganda'/syn OR 'rwanda'/syn OR 'burundi'/syn OR 'kenya'/syn OR 'somalia'/syn OR 'tanzania'/syn OR 'zambia'/syn OR 'mozambique'/syn OR 'madagascar'/syn OR 'seychelles'/syn OR 'mauritius'/syn OR 'comoros'/syn OR 'botswana'/syn OR 'malawi'/syn OR 'brunei'/syn OR 'burma'/syn OR 'myanmar'/syn OR 'cambodia'/syn OR 'east timor'/syn OR 'indonesia'/syn OR 'laos'/syn OR 'malaysia'/syn OR 'philippines'/syn OR 'singapore'/syn OR 'thailand'/syn OR 'vietnam'/syn OR 'india'/syn OR 'papua new guinea'/syn OR 'australia'/syn OR 'solomon islands'/syn OR 'vanuatu'/syn OR 'new caledonia'/syn) AND [embase]/lim

4,621,441

#1

('patient care'/syn OR 'vital statistics'/syn OR 'wounds and injuries'/syn OR 'urogenital tract disease'/syn OR 'stomatognathic diseases'/syn OR 'skin and connective tissue diseases'/syn OR 'respiratory tract diseases'/syn OR 'pathological conditions, signs and symptoms'/syn OR 'otorhinolaryngologic diseases'/syn OR 'occupational diseases'/syn OR 'nutritional and metabolic diseases'/syn OR 'nervous system diseases'/syn OR 'neoplasms'/syn OR 'musculoskeletal disease'/syn OR 'infection'/syn OR 'immune system diseases'/syn OR 'hemic and lymphatic diseases'/syn OR 'eye diseases'/syn OR 'endocrine disease'/syn OR 'environmental disease'/syn OR 'digestive system diseases'/syn OR 'congenital, hereditary, and neonatal diseases and abnormalities'/syn OR 'chemically induced disorder'/syn OR 'cardiovascular diseases'/syn) AND [embase]/lim

Date of search: August 18, 2023

Records: 4,020

EconLit

(('wildfires' or 'fire' or 'deforestation') and ((((((((((('tropical climate' or 'climate tropical' or 'climates tropical' or 'tropical climates' or 'rainforest' or 'rainforests' or 'rain forest' or 'forest rain' or 'rain
forests' or 'tropical rainforest' or 'rainforest tropical' or 'rainforests tropical' or 'tropical rainforests' or 'Amazon' or 'Brazil' or 'Argentina' or 'Peru' or 'Ecuador' or 'Bolivia' or 'Colombia' or 'Venezuela' or 'Guyana' or
'Suriname' or 'French Guiana' or 'Paraguay' or 'Panama' or 'El Salvador' or 'Belize' or 'Costa Rica' or 'El Salvador' or
'Guatemala' or 'Honduras' or 'Nicaragua' or 'Panama' or 'Anguilla' or 'Antigua) and Barbuda') or 'Aruba' or 'Bahamas' or
'Barbados' or 'British Virgin Islands' or 'Cayman Islands' or 'Cuba' or 'Dominica' or 'Dominican Republic' or 'Grenada'
or 'Guadeloupe' or 'Haiti' or 'Jamaica' or 'Martinique' or 'Montserrat' or 'Netherlands Antilles' or 'Puerto Rico' or
'Saint Barthelemy' or 'Saint Kits) and Nevis') or 'Saint Lucia' or 'Saint Martin' or 'Saint Vincent) and the

For peer review only

Grenadines') or 'Trinidad) and Tobago') or 'United States Virgin Islands' or 'Mexico' or 'Hawaii' or 'Cape Verde' or 'Sao Tome) and Principe') or 'Gambia' or 'Senegal' or 'Guinea-Bissau' or 'Guinea' or 'Sierra Leone' or 'Liberia' or 'Ivory Coast' or 'Ghana' or 'Togo' or 'Benin' or 'Nigeria' or 'Cameroon' or 'Central African Republic' or 'South Sudan' or 'Ethiopia' or 'Equatorial Guinea' or 'Gabon' or 'Congo' or 'Democratic Republic of the Congo' or 'Uganda' or 'Rwanda' or 'Burundi' or 'Kenya' or 'Somalia' or 'Tanzania' or 'Zambia' or 'Mozambique' or 'Madagascar' or 'Seychelles' or 'Mauritius' or 'Comoros' or 'Botswana' or 'Malawi' or 'Brunei' or 'Burma' or 'Myanmar' or 'Cambodia' or 'East Timor' or 'Indonesia' or 'Laos' or 'Malaysia' or 'Philippines' or 'Singapore' or 'Thailand' or 'Vietnam' or 'India' or 'Papua New Guinea' or 'Australia' or 'Solomon Islands' or 'Vanuatu' or 'New Caledonia') and ((((((((((((('patient care' or 'vital statistics' or 'wounds) and injuries') or 'urogenital tract disease' or 'stomatognathic diseases' or 'skin) and connective tissue diseases') or 'respiratory tract diseases' or 'pathological conditions, signs) and symptoms') or 'otorhinolaryngologic diseases' or 'occupational diseases' or 'nutritional) and metabolic diseases') or 'nervous system diseases' or 'neoplasms' or 'musculoskeletal disease' or 'infection' or 'immune system diseases' or 'hemic) and lymphatic diseases') or 'eye diseases' or 'endocrine disease' or 'environmental disease' or 'digestive system diseases' or 'congenital, hereditary,) and neonatal diseases and abnormalities') or 'chemically induced disorder' or 'cardiovascular diseases')).mp. [mp=tx, bt, ti, ab, ct, hw, id]

Date of search: August 18, 2023

Records: 464

For peer review only

Appendix II - Data extraction form

General information	
Reviewer who performed the data extraction:	
Date of the data extraction performed:	
Paper database:	
**Study identification**	
Publication full title:	
DOI number:	
First author name:	
Year of publication:	
Journal of publication:	
Aim of study:	
**Exposure and data source**	
Area (city, region, country):	
Exposure:	
Exposure temporal level:	
Exposure temporal duration:	
Origin of the exposure data source:	
Origin of the outcome data source:	
**Method**	
The statistical model applied:	
**Study design and findings**	
Study population:	
Health outcome:	
Type of disease:	
Study findings as reported by the authors:	
Report the following list:<br>1. measure of association + standard errors for every different regression specification<br>2. Subgroups estimates<br>3. Confounders used<br>4. Identification strategy if appliable	
**Conclusions**	
Study limitations identified by the team:	

For peer review only

Appendix III - Ongoing work

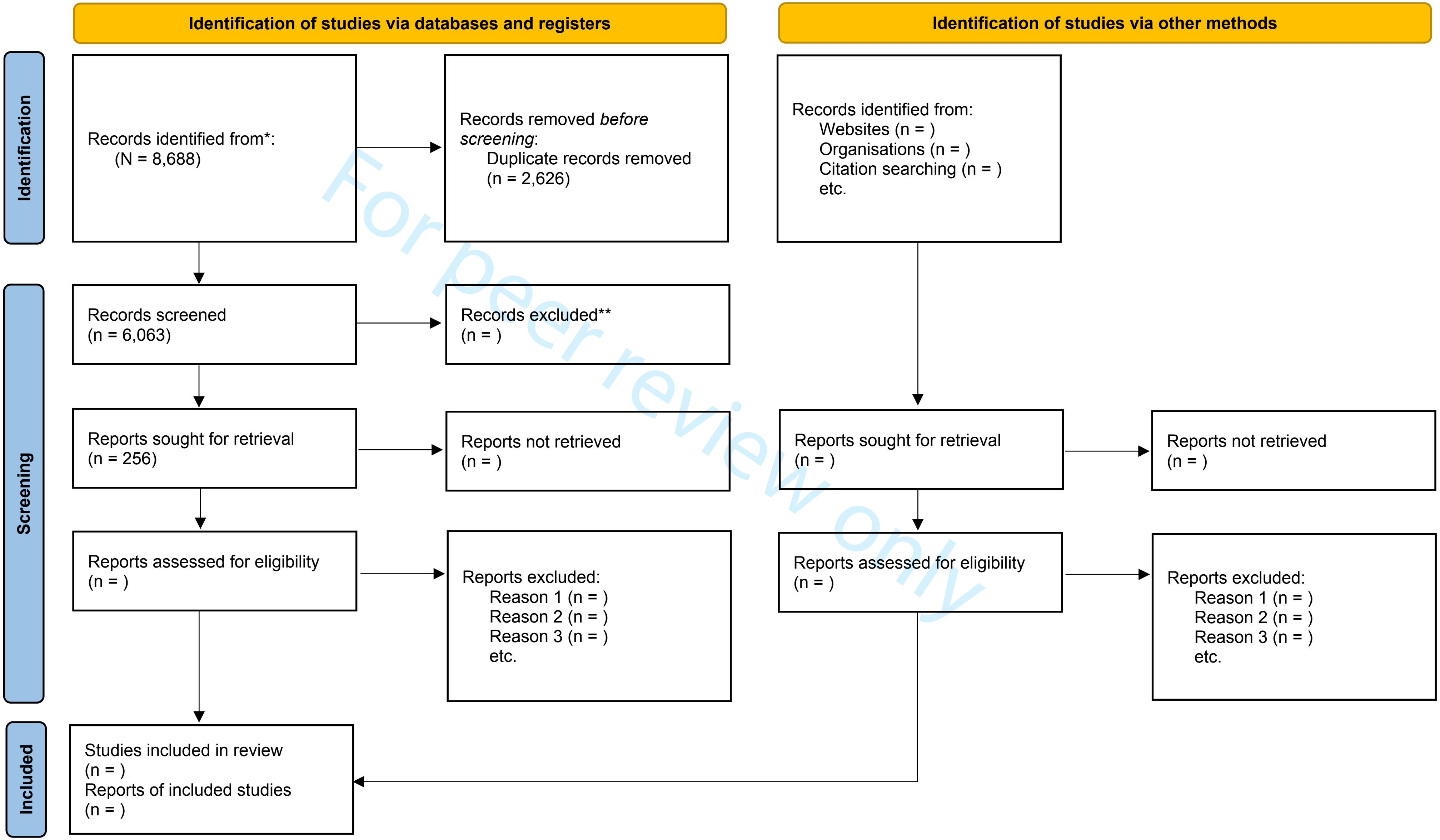

From: Page MJ, McKenzie JE, Bossuyt PM, Boutron I, Hoffmann TC, Mulrow CD, et al. The PRISMA 2020 statement: an updated guideline for reporting systematic reviews. BMJ 2021;372:n71. doi: 10.1136/bmj.n71.

For peer review only

BMJ Open

Wildfire, deforestation, and health in tropical rainforest areas: a scoping review protocol

Journal:	*BMJ Open*
Manuscript ID	bmjopen-2023-082381.R1
Article Type:	Protocol
Date Submitted by the Author:	20-Mar-2024
Complete List of Authors:	Casais, Gustavo; Fiocruz Brasilia, Center of Data and and Knowledge Integration for Health<br>Guimarães, Nathalia ; Federal University of Minas Gerais, Departament of Nutrition<br>Cortes, Taísa ; Center of Data and Knowledge Integration for Health (CIDACS) - Fiocruz/BA, Center of Data and and Knowledge Integration for Health<br>Pescarini , Julia ; London School of Hygiene & Tropical Medicine; Center of Data and Knowledge Integration for Health (CIDACS)<br>Rebouças de Magalhães, Poliana; Center of Data and Knowledge Integration for Health (CIDACS) - Fiocruz/BA<br>Wells, Valerie; University of Glasgow<br>de Sousa Filho, José Firmino; Center of Data and Knowledge Integration for Health (CIDACS) - Fiocruz/BA<br>Delgado Neves, Danielson; Center of Data and Knowledge Integration for Health (CIDACS) - Fiocruz/BA<br>Shimonovich , Michal ; University of Glasgow<br>Olsen, Jonathan; University of Glasgow<br>de Carvalho Neto, Edgar Marcelino; Center of Data and Knowledge Integration for Health (CIDACS) - Fiocruz/BA<br>Cooper, Philip; Universidad Internacional del Ecuador; St George's, University of London<br>Katikireddi, Srinivasa; University of Glasgow<br>Emanuel, Lucas; Federal University of Bahia; Center of Data and Knowledge Integration for Health (CIDACS) - Fiocruz/BA<br>Andrade , Roberto F. S. ; Center of Data and Knowledge Integration for Health (CIDACS) - Fiocruz/BA; Federal University of Bahia<br>Ferreira dos Santos, Gervasio; Center of Data and Knowledge Integration for Health (CIDACS) - Fiocruz/BA; Federal University of Bahia<br>Barreto, Mauricio; Center of Data and Knowledge Integration for Health (CIDACS) - Fiocruz/BA; Federal University of Bahia
**Primary Subject Heading**:	Public health
Secondary Subject Heading:	Epidemiology, Global health, Health policy, Health economics
Keywords:	EPIDEMIOLOGY, PUBLIC HEALTH, STATISTICS & RESEARCH METHODS, TROPICAL MEDICINE

Wildfire, deforestation, and health in tropical rainforest areas: a scoping review protocol

Authors

Gustavo Casais*, PhD, Center of Data and Knowledge Integration for Health (CIDACS) - Fiocruz/BA, Parque Tecnológico da Bahia, Edf. Tecnocentro, R. Mundo, 121 - sala 315 - Trobogy, Salvador - BA, Brazil, 41745-715, gustavo.casais@hotmail.com, +55 (71) 99127 3345.

Nathalia Sernizon Guimarães, PhD, Professor at Departament of Nutrition, Federal University of Minas Gerais, Belo Horizonte, Minas Gerais, Brazil zip code 30130100.

Taísa Rodrigues Cortes, PhD, Center of Data and Knowledge Integration for Health (CIDACS) - Fiocruz/BA, Parque Tecnológico da Bahia, Edf. Tecnocentro, R. Mundo, 121 - sala 315 - Trobogy, Salvador - BA, Brazil, 41745-715, taisacortes@gmail.com, +55 (71) 99127 3345.

Julia Pescarini, PhD, London School of Hygiene and Tropical Medicine, Center of Data and and Knowledge Integration for Health (CIDACS) - Fiocruz/BA, Keppel Street, London, WC1E 7HT, United Kingdom, julia.pescarini1@lshtm.ac.uk, +44 (0)20 7636 8636.

Poliana Rebouças de Magalhães, PhD, Center of Data and Knowledge Integration for Health (CIDACS) - Fiocruz/BA, Parque Tecnológico da Bahia, Edf. Tecnocentro, R. Mundo, 121 - sala 315 - Trobogy, Salvador - BA, Brazil, 41745-715, poliana.reboucas@fiocruz.br, +55 (71) 99127 3345.

Valerie Wells, Ms, MRC/CSO Social and Public Health Sciences Unit, University of Glasgow. 90 Byres Road, Glasgow, G12 8TB, United Kingdom, Valerie.Wells@glasgow.ac.uk, +44 0141 330 4042.

José Firmino de Sousa Filho, PhD, Center of Data and Knowledge Integration for Health (CIDACS) - Fiocruz/BA, Parque Tecnológico da Bahia, Edf. Tecnocentro, R. Mundo, 121 - sala 315 - Trobogy, Salvador - BA, Brazil, 41745-715, jose.ffilho@fiocruz.br, +55 (71) 99127 3345.

Danielson Jorge Delgado Neves[1], PhD, Center of Data and Knowledge Integration for Health (CIDACS) - Fiocruz/BA, Parque Tecnológico da Bahia, Edf. Tecnocentro, R. Mundo, 121 - sala 315 - Trobogy, Salvador - BA, Brazil, 41745-715, danielson.neves@fiocruz.br, +55 (71) 99127 3345.

Michal Shimonovich, PhD, MRC/CSO Social & Public Health Sciences Unit, University of Glasgow, Berkeley Square, 99 Berkeley Street, Glasgow G3 7HR, United Kingdom, Michal.Shimonovich@glasgow.ac.uk, +44 0141 330 4042.

Jonathan R Olsen, PhD, MRC/CSO Social & Public Health Sciences Unit, University of Glasgow, Berkeley Square, 99 Berkeley Street, Glasgow G3 7HR, United Kingdom, Jonathan.Olsen@glasgow.ac.uk, +44 0141 330 4042.

Edgar Marcelino de Carvalho Neto, PhD, Center of Data and Knowledge Integration for Health (CIDACS) - Fiocruz/BA, Parque Tecnológico da Bahia, Edf. Tecnocentro, R. Mundo, 121 - sala 315 - Trobogy, Salvador - BA, Brazil, 41745-715, edgar.neto@fiocruz.br, +55 (71) 99127 3345.

For peer review only

Philip J. Cooper, PhD, St George's, University of London; School of Medicine, Universidad Internacional del Ecuador, Av. Simón Bolívar y Av. Jorge Fernández, Quito, Ecuador, pcooper@sgul.ac.uk, +593 2 2985 600.

Srinivasa Vittal Katikireddi, PhD, MRC/CSO Social & Public Health Sciences Unit, University of Glasgow, Berkeley Square, 99 Berkeley Street, Glasgow G3 7HR, United Kingdom, Vittal.Katikireddi@glasgow.ac.uk, +44 0141 330 4042.

Lucas Emanuel, Federal University of Bahia; Center of Data and Knowledge Integration for Health (CIDACS) - Fiocruz/BA, Parque Tecnológico da Bahia, Edf. Tecnocentro, R. Mundo, 121 - sala 315 - Trobogy, Salvador - BA, Brazil, 41745-715, lucasemanuel@ufba.br, +55 (71) 99127 3345.

Dr. Roberto Fernandes Silva Andrade, Federal University of Bahia; Center of Data Knowledge Integration for Health (CIDACS) - Fiocruz/BA, Parque Tecnológico da Bahia, Edf. Tecnocentro, R. Mundo, 121 - sala 315 - Trobogy, Salvador - BA, Brazil, 41745-715, randrade@ufba.br, +55 (71) 99127 3345.

Dr. Gervasio Ferreira dos Santos, Federal University of Bahia; Center of Data Knowledge Integration for Health (CIDACS) - Fiocruz/BA, Parque Tecnológico da Bahia, Edf. Tecnocentro, R. Mundo, 121 - sala 315 - Trobogy, Salvador - BA, Brazil, 41745-715, gervasios@ufba.br, +55 (71) 99127 3345.

Mauricio L. Barreto, Federal University of Bahia; Center of Data Knowledge Integration for Health (CIDACS) - Fiocruz/BA, Parque Tecnológico da Bahia, Edf. Tecnocentro, R. Mundo, 121 - sala 315 - Trobogy, Salvador - BA, Brazil, 41745-715, mauricio@ufba.br, +55 (71) 99127 3345.

*Corresponding author, Email: gustavo.casais@hotmail.com.

Word count: 2,501

Keywords: Wildfire; Deforestation; Tropical Climate; Rainforest; Health

For peer review only

Wildfire, deforestation, and health in tropical rainforest areas: a scoping review protocol

Authors

Gustavo Casais[1], Nathalia Sernizon Guimarães[2], Taísa Rodrigues Cortes[1], Julia Pescarini[1,3], Poliana Rebouças de Magalhães[1], Valerie Wells[4], José Firmino de Sousa Filho[1], Danielson Jorge Delgado Neves[1], Michal Shimonovich[4], Jonathan R Olsen[4], Edgar Marcelino de Carvalho Neto[1], Philip J. Cooper[5,6], Srinivasa Vittal Katikireddi[4], Lucas Emanuel[1,7], Roberto F. S. Andrade[1,7], Gervasio Ferreira dos Santos[1,7], Mauricio L. Barreto[1,7], on behalf of the Unit on the Social and Environmental Determinants of Health Inequalities (SEDHI)

[1]Center for Data and Knowledge Integration for Healthcare (CIDACS)

[2]Federal University of Minas Gerais

[3]London School of Hygiene and Tropical Medicine

[4]MRC/CSO Social and Public Health Sciences Unit, University of Glasgow

[5]St George's, University of London

[6]School of Medicine, Universidad Internacional del Ecuador, Quito, Ecuador

[7]Federal University of Bahia

Abstract

Introduction: Wildfires and deforestation potentially have direct effects on multiple health outcomes as well as indirect consequences for climate change. Tropical rainforest areas are characterized by high rainfall, humidity, and temperature, and they are predominantly found in low- and middle-income countries. This study aims to synthesize the methods, data, and health outcomes reported in scientific papers on wildfires and deforestation in these locations.

Methods and analysis: We will carry out a scoping review according to the Joanna Briggs Institute's manual for scoping reviews and the framework proposed by Arksey and O'Malley, and Levac et al. The search for articles was performed on August 18, 2023, in 16 electronic databases using MeSH Terms and adaptations for each database from database inception. The search for local studies will be complemented by the manual search in the list of references of the studies selected to compose this review. We screened studies written in English, French, Portuguese and Spanish. We included quantitative studies assessing any human disease outcome, hospitalization, and vital statistics in regions of tropical rainforest. We exclude qualitative studies and quantitative studies whose outcomes do not cover those of interest. The text screening was done by two independent reviewers. Subsequently, we will tabulate the data by the origin of the data source used, the methods, and the main findings on health impacts the extracted data. The results will provide descriptive statistics, along with visual representations in diagrams and tables, complemented by narrative summaries as detailed in the JBI guidelines.

Ethics and dissemination: The study does not require an ethical review as it is meta-research and utilizes published, deidentified secondary data sources. The submission of results for publication in a peer-reviewed journal and presentation at scientific and policymakers' conferences is expected.

Link to the protocol record in the Open Science Framework (OSF): https://osf.io/pnqc7/.

For peer review only

Strengths and limitations of this study:

- To the best of our knowledge, this scoping review will be the first to assess the health impacts of wildfires and deforestation across a broad range of tropical rainforest regions.
- The search will include several different databases, such as those from Latin America and Africa.
- It will include manuscripts in English, French, Portuguese and Spanish from database inception.
- The ultimate selection of papers will be heavily influenced by the resolution of the shapefile used to delineate the tropical rainforest biome. This sensitivity to the map's resolution may result in the improper exclusion or inclusion of studies.
- A major limitation is the absence of critical appraisal. This means studies with potentially low relevance, reliability, validity and applicability might be included.

Introduction

One of the significant contributors to climate change is improper land use resulting from agriculture, logging, and mining, which potentially leads to wildfires and deforestation [1] [2]. Currently, wildfires and deforestation have been increasingly drawing attention for their potential consequences, not only for climate change but also for the health outcomes of both local and global populations. Governments across the world have been expressing this concern by adopting climate mitigation policies to contain environmental degradation. Understanding the health effects of wildfires and deforestation on populations in low- and middle-income countries is critical for designing evidence-based and successful mitigation plans and policies [3].

Tropical rainforests are home to not only a vast array of animal and plant species but also play a vital role in sustaining human well-being [4]. These ecosystems provide essential resources such as cocoa, coffee beans, bananas, vanilla, and cinnamon, which are used in everyday products. Furthermore, they are a rich source of chemical compounds instrumental in the development of medicines. These biomes are the ancestral homes of Indigenous and traditional peoples who not only live within these ecosystems but also actively preserve them along with their cultures. Rainforests are crucial in stabilizing the climate and maintaining the water cycle, contributing to the overall balance of global weather patterns [5] [6]. Without these rainforests, the dynamics and control of zoonotic diseases and vector-borne infections would be significantly disrupted [7]. The loss of rainforests would have severe consequences for the economy, global biodiversity and ecosystem services [8].

While wildfires and deforestation often stem from common causes, they are also influenced by distinct drivers. Human activities, such as land clearing for agriculture, logging and development, play a significant role in both phenomena [9] [10]. In low- and middle-income countries, where

For peer review only

agriculture constitutes a substantial portion of the economy, fluctuations in global demand can drive the expansion of agricultural areas, contributing to deforestation and increasing wildfire susceptibility [11] [12]. Additionally, inadequate policies, weak enforcement of regulations, and governance issues exacerbate both deforestation and wildfire risks by permitting unsustainable land use practices and inadequately allocating resources for fire prevention and suppression efforts [13]. Moreover, the escalating frequency and intensity of extreme events, such as droughts, extreme temperatures, and storms, linked to climate change, have been amplifying wildfire occurrences globally over the past few decades [14]. Also, due to the usually high humidity in tropical rainforests, wildfires very rarely occur by natural causes, so that both deforestations and wildfires are much closely related to human activity [15] [16].

Wildfires have the potential to cause bodily injuries, impact housing infrastructure, and release toxic gases and particulate matter into the air [17]. Exposure to smoke from wildfires can cause acute respiratory illness and exacerbate existing disease, especially among children and elderly [18]. Additionally, the long-term effects of accumulated exposures to wildfires may be multiple including premature death, cardiovascular disease, cancer, respiratory illness, mental health, and other chronic conditions [19].

Deforestation has different causal mechanisms for public health outcomes. Deforestation can alter environmental niches, changing habitats for parasites and insects, including disease-carrying mosquitoes, which may increase the human risk of contracting vector-borne diseases such as malaria and dengue [20] [7] [21]. Additionally, in the long run, deforestation can reduce the level of water in the atmosphere, lead to soil erosion, desertification, flooding, and increase the local temperature [22] [23] [24].

We have noticed that previous reviews on the health impacts of wildfires and deforestation have some limitations. They often focus on specific population groups or a limited number of health outcomes, and they might not search comprehensively across databases or years [19] [25] [26] [27] [28] [29] [30]. Notably, no review has specifically addressed the health effects of wildfires in tropical rainforest areas, which are mainly found in low- and middle-income countries. These regions are home to many Indigenous and marginalized groups who are particularly vulnerable due to limited resources, higher risk of health problems, and limited access to healthcare [31] [32] [33]. Tropical climate, characterized by elevated temperatures, intense rainfall, and high humidity, can further impact health conditions [34] [35]. Moreover, the most recent reviews on the health effects of deforestation are at least four years old, indicating the need for an updated assessment [29] [30].

For peer review only

Conducting an updated review is crucial, especially considering the recent escalation of wildfires and deforestation. This issue is particularly significant in Brazil, where fire outbreaks have been steadily increasing, even though they have not reached the peak observed in 2004 [36] [37]. Such a review will help identify and understand the specific ways in which wildfires and deforestation affect health outcomes, hospitalizations, and vital statistics, the quantitative methods employed, and the data sources used for these analyses.

Other critical areas of investigation include geographical mapping and the analytical methods employed for analysis. There is a wide variety of information sources that provide mapping for the occurrence of wildfires and deforestation, differentiating based on geographical scale and the time period of change. Disparities in high-quality environmental data around the world highlight how some places possess better information than others, which can be considered part of the phenomenon known as the 'digital divide' [38]. Additionally, various analytical methods exist to study how wildfires and deforestation impact people's health. The outcomes of such analyses heavily rely on the modelling techniques employed, underscoring the crucial need to understand and utilize an array of available methods. This understanding is pivotal in creating thorough and accurate insights to inform the creation of future high-quality research and understand what key research gaps exist.

This scoping review aims to comprehensively synthesize the intricate relationships between wildfires, deforestation, and their impact on health outcomes in tropical rainforest regions. Our more specific objectives will be to characterize: (i) the health outcomes affected by wildfires and deforestation in the tropical areas; (ii) the methods used to identify and measure their impact; (iii) the data sources related to the wildfires and deforestation; (iv) and the policy recommendations from the studies. This will equip policymakers and researchers with essential information about this research area, highlighting knowledge gaps and paving the way for future research and development.

Methods and Analysis

This scoping review protocol follows the guidelines of the Joanna Briggs Institute. The protocol is based on the framework suggested by Peters, et al [39], Arksey and O'Malley [40], and enhanced by Levac et al. [41]. It was written according to the checklist provided by the Preferred Reporting Items for Systematic Reviews and Meta-Analyses Extension for Scoping Reviews (PRISMA-ScR) [42].

The scoping review will follow the steps below: (1) identifying the research question; (2) identifying relevant studies; (3) study selection; (4) charting the data; (5) collating, summarising, and reporting the results. The scoping review protocol was previously registered with the Open Science Framework to identify ongoing reviews and avoid unnecessary duplication of research [43].

For peer review only

Step 1: identifying the research question

To enhance the organization of our research question and the criteria for inclusion and exclusion we adhered to the mnemonic PCC (Population, Concept, Context), which is described with the research question in the Table 1.

According to the Oxford Dictionary, wildfire means: "a very big fire that spreads quickly and burns natural areas like woods, forests and grassland". We adopt this definition; therefore, we consider any large fire that occur in different types of vegetation which can affect urban, peri urban or rural area. The deforestation is usually associated with human activity pursuing an economic purpose (e.g., farming, timber logging, expansion and infrastructure, and mining). The tropical rainforest is a warm and humid biome characterized by year-round rainfall. Renowned for its thick layers of vegetation, it consists of three distinct canopy levels located between the Tropic of Cancer and the Tropic of Capricorn [44]. We will consider the deforestation of the tropical forests located in urban, peri-urban, or rural areas.

Table 1 – Scoping review questions and PCC mnemonic

Question	Population (P)	Concept (C)	Context (C)
1. What are the impacts of the wildfire and deforestation on the health outcomes, hospitalization, and vital statistics in the tropical rainforest areas?<br>2. What are the methods and data sources used for this assessment?	All individuals	Health impacts of wildfires and deforestation	All the areas located in the tropical rainforests

Step 2: Identifying relevant studies

Data sources

The search for scientific articles were conducted on August 18, 2023, across several databases: (1) Nursing Database (BDENF – Enfermagem), (2) National Bibliography in Argentine Health Sciences (BINACIS), (3) Coleciona SUS, (4) Desastres, (5) EconLit, (6) Embase, (7) Latin American and Caribbean Literature in Health Sciences (Lilacs), (8) Literature in Health Sciences from Caribbean countries (MedCaribe), (9) MEDLINE, (10) MEDLINE/PubMed, (11) Virtual Health Library of the Ministry of Health of Peru (MINSAPERU), (12) Literature from the Pan American Health Organization Headquarters Library (PAHO-IRIS), (13) Health Documentation Network in Mozambique (RDSM), (14) Recursos Multimídia, (15) Scopus, (16) Western Pacific Region Index

For peer review only

Medicus (WPRIM). Access to Scopus database will be via the Capes Platform, while the databases corresponding to the previous identification number: (1), (2), (3), (4), (7), (8), (9), (11), (12), (13), (14), and (16) are available via the Virtual Health Library platform (Biblioteca Virtual da Saúde in Portuguese). To strengthen this review, we will also perform a manual search of the references in the included studies.

Table 2 – Search terms by topics

1. "Wildfires"[MeSH Terms] OR "Deforestation"[All Fields]
2. 'All categories of human diseases' OR "Vital Statistics"[MeSH Terms] OR "Patient Care"[MeSH Terms]
3. "Rainforest"[MeSH Terms] OR 'All country names with tropical rainforest'
4. (1) AND (2) AND (3)

Note: The complete list of terms used can be found in the Appendix I.

Search strategy

The search strategy will be defined for each database, following the inclusion and exclusion criteria. The search terms were used according to the Medical Subject Headings (MeSH) and the respective Entry Terms. The remaining databases were adapted by Emtree and DeCS. The expression terms were categorized into three broad aspects according to Table 2: (i) the exposure, including the wildfires and the deforestation terms, (ii) a comprehensive list of diseases, hospitalization, vital statistics terms, and (iii) tropical rainforest areas, including a list of countries with tropical rainforests. The countries that harbour tropical rainforests were selected according to two maps [44] [45]. The expression terms will be combined with the Boolean operators 'AND' and 'OR' in the refinement. The complete search terms in every database are in the Appendix I.

Step 3: Study selection

To ensure consistent evaluation of the literature, a two-stage screening process was employed by two reviewers. This process involved initial screening of titles and abstracts, followed by a more in-depth review of full texts. Regarding any disagreements among the authors, they were resolved either through consensus or by the decision of one or two additional authors.

The process will be registered in a flowchart of the review process according to the PRISMA-ScR [42]. All studies were exported to the Rayyan Qatar Computing Research Institute (Rayyan®), and then deduplicated by one reviewer. The partial results of the text screening are available in Appendix II.

For peer review only

Inclusion criteria

To determine and choose pertinent publications concerning the topic, the subsequent inclusion criteria will be applied: (i) quantitative studies from database inception, such as correlational, ecological, cohort, experimental, and cross-sectional studies, (ii) any individual or population groups exposed to wildfires, wildfire smoke or deforestation regardless of the exposure duration, (iii) any disease, hospitalization, or vital statistics, considered here as birth, death rate, and life expectancy, (iv) self-reported health condition. Only studies written in English, French, Portuguese and Spanish will be considered for inclusion.

Exclusion criteria

Studies will be excluded according to the following criteria: (i) theoretical studies; literature review (e.g., scoping review and systematic review), letter and editorials; (ii) qualitative studies (interviews, case studies etc.); (iii) environmental change only (e.g., extinction of wildlife, mosquitoes' habitats); (iv) air pollution only (e.g., air pollution from factories, mines, vehicles, without any relation to wildfires); (v) indoor fire; (vi) we will exclude studies solely focusing on health inequalities according to PROGRESS Plus; and (vii) studies in which the exposed population is entirely outside the tropical area, as delimited by the Tropic of Cancer and the Tropic of Capricorn.

The expansive nature of this review, encompassing diverse outcomes, countries, and databases, necessitates a streamlined approach. To ensure a comprehensive yet efficient exploration of the literature within the constraints of time and resources, we have opted to focus on quantitative studies. Quantitative studies often provide a more readily comparable data set, facilitating the data extraction and the synthesis of the existing research landscape in this complex field. Additionally, the exclusion of the grey literature also relies on the constraints of time and resources.

For peer review only

Step 4: Charting the data

The data of interest will be extracted with the data extraction tables and by filling out the data extraction form (in the Appendix III) in Microsoft Excel. During the pilot stage, two reviewers will independently conduct the task. Afterward, one reviewer will proceed, while the work will be reviewed by a second reviewer for quality assurance. The results will be categorised according to the review questions and charted in an iterative process, allowing the reviewers to continuously update these charts when additional unforeseen data are encountered. The data extraction table will be developed and tested, containing variables on the study reference (year of publication, author, journal, full title), intervention type, exposure, and data source (e.g., country, origin of the exposure data source), methods and findings (study design and modelling, health outcome, control, and treated

group, point estimate, and causal identification strategy, lag between exposure and the health consequences and limitations of the study.

Step 5: Collating, summarising and reporting the results

All gathered data will be displayed in either tabular or diagrammatic formats to visually summarise the outcomes of the studies. Initially, a table containing comprehensive information about the selected papers will be provided, such as the number of studies, study design, exposure assessment (temporal and spatial scale, and data sources), statistical methods (statistical models and identification strategies), characteristics of study populations, and the countries where the studies were conducted. The data will be categorized separately for wildfires and deforestation. Different tables will be used to describe the detailed methods and data sources, followed by tables focused on findings and their subgroups. Finally, we will consider the overall implications of the results to ensure that the scoping review will provide relevant answers to the two main research questions previously posed.

Discussion

The resulting scoping review will offer novel insights by synthesizing a wide array of health outcomes associated with wildfires and deforestation. It will elucidate the methodologies and data sources utilized in existing literature to assess the impact of these phenomena on public health. Moreover, the review will provide policymakers with actionable recommendations derived from studies addressing the health effects of wildfires and deforestation, including considerations of magnitude and temporal lag between exposure and outcome.

While the scoping review will offer valuable insights, it is important to acknowledge that it may encounter certain limitations. The level of detail in the map defining the tropical rainforest biome will significantly influence which studies are included in the final selection. This can lead to the unintended exclusion or inclusion of relevant research. The exclusion of the grey literature is contingent upon limitations in both time and resources available for the study. It often contains valuable insights and data that may not be found in traditional academic sources, potentially resulting in an incomplete understanding of the topic under investigation. Additionally, the scoping review will not conduct critical appraisal. This means they can't assess the quality of included studies, potentially incorporating biased or flawed research. Consequently, drawing definitive conclusions about the effectiveness of interventions or pinpointing areas needing strong future studies becomes difficult. While valuable for initial exploration, these limitations necessitate cautious interpretation of the findings.

Patient and public involvement

For peer review only

There was no patient or public involvement in the design of this scoping review protocol.

Ethics and dissemination:

The study is exempt from ethics review as it is meta-research and utilizes published, deidentified secondary data sources. The submission of results for publication in a peer-reviewed journal and presentation at scientific and policymakers' conferences is expected.

Contributors

G.C. is the primary and corresponding author and was responsible for the first and all subsequent drafts of this scoping review protocol. Study conception: J.F.S.F, P.J.C., J.O., S.V.K., L.E., R.F.S.A., G.F.S., M.L.B. Designed the search strategy: J.P., P.B.R., V.W., N.S.G., M.S. Data search: G.C., J.P., N.S.G., M.S., E.M.C.N. Text screening: G.C., T.C. Geographical map: G.C., D.J.D.N. All authors participated in discussions on the study design and critically revised drafts for improvements.

Competing Interests

None.

Funding

This research was funded by the NIHR (NIHR134801) using UK aid from the UK Government to support global health research and by the Wellcome Trust grant (226306/Z/22/Z) awarded to the CIDACS Climate and Environmental Platform (CIDACS-Clima). The views expressed in this publication are those of the author(s) and not necessarily those of the NIHR or the UK government. JO, MS, VW and SVK are employed by the MRC/CSO Social and Public Health Sciences Unit, University of Glasgow, and supported by the Medical Research Council [grant numbers MC_UU_00022/2; MC_UU_00022/4; and Chief Scientist Office [grant numbers SPHSU17; SPHSU19]. The researchers were independent of the funders; the funders had no role in the study design, data collection, analysis and interpretation of data, the decision to publish, or the preparation of the manuscript.

References

[1] P. W. Ellis, T. Gopalakrishna, R. C. Goodman, F. E. Putz, A. Roopsind, P. M. Umunay, J. Zalman, E. A. Ellis, M. Karen, T. G. Gregoire and B. W. Griscom, "Reduced-impact logging for climate change mitigation (RIL-C) can halve selective logging emissions from tropical forests," *Forest Ecology and Management,* vol. 438, 2019.

[2] L. Ying, H. Cheng, Z. Shen, P. Guan, C. Luo and X. Peng, "Relative humidity and agricultural activities dominate wildfire ignitions in Yunnan, Southwest China: Patterns, thresholds, and implications," *Agricultural and Forest Meteorology,* vol. 307, 2021.

[3] S. M. Hartinger, M. Yglesias-González, L. Blanco-Villafuerte, Y. K. Palmeiro-Silva, A. G. Lescano, A. Stewart-Ibarra, ... and M. Romanello, "The 2022 South America report of The Lancet Countdown on health and climate change: trust the science. Now that we know, we must act.," *The Lancet Regional Health–Americas,* vol. 20, 2023.

[4] C. Y. Shimamoto, A. A. Padial, C. M. Rosa and M. C. M. Marques, "Restoration of ecosystem services in tropical forests: A global meta-analysis," *Plos One,* vol. 13(12), 2018.

[5] D. Ellison, M. N. Futter and K. Bishop, "On the forest cover–water yield debate: from demand- to supply-sind thinking," *Global Change Biology,* vol. 18, 2012.

[6] F. B. F. Silvio, K. M. P. M. B. Ferraz, C. C. Cassiano, P. H. S. Brancalion, D. T. A. d. Luz, T. N. Azevedo, L. R. Tambosi and J. P. Metzger, "How good are tropical forest patches for ecosystem services?," *Landscape Ecology,* vol. 29, 2014.

[7] J. H. Ellwanger, B. Kulmann-Leal, V. L. Kaminski, J. Valverde-Villegas, A. B. G. VEIGA, F. R. Spilki, ... and J. A. B. Chies, "Beyond diversity loss and climate change: Impacts of Amazon deforestation on infectious diseases and public health," *Anais da Academia Brasileira de Ciências,* vol. 92.

[8] C. Vera, J. Baez, M. Douglas, C. B. Emmanuel, J. Marengo, J. Meitin, M. Nicolini, J. Nogues-Paegle, J. Paegle, O. Penalba, P. Salio, C. Saulo, M. A. Silva Dias, P. Silva Dias and E. Zipser, "The South American Low-Level Jet Experiment," *Bulletin of the American Meteorological Society,* vol. 87, 2006.

[9] P. M. Lemieux, C. C. Lutes and D. A. Santoianni, "Emissions of organic air toxics from open burning: a comprehensive review," *Progress in energy and combustion science,* vol. 30, no. 1, 2004.

[10] R. Xu, P. Yu, M. J. Abramson, F. H. Johnston, J. M. Samet, M. L. Bell, ... and Y. Guo, "Wildfires, global climate change, and human health," *New England Journal of Medicine,* vol. 383, no. 22, pp. 2173-2181, 2020.

[11] D. Da Mata and M. Dotta, "Commodity booms and the environment," SSRN 3900793, 2021.

[12] P. Dasgupta, The economics of biodiversity: the Dasgupta review, Hm Treasury, 2021.

[13] J. Assunção, C. Gandour and R. Rocha, "Deforestation slowdown in the Brazilian Amazon: prices or policies?," *Environment and Development Economics,* vol. 20, pp. 697-722, 2015.

[14] Centre for Research on the Epidemiology of Disasters, United Nations Office for Disaster Risk Reduction, "The human cost of disasters: an overview of the last 20 years (2000-2019)," 2020.

[15] M. Adámek, Z. Jankovská, V. Hadincová, E. Kula and J. Wild , "Drivers of forest fire occurrence in the cultural landscape of Central Europe," *Landscape Ecology,* vol. 33, p. 2031–2045, 2018.

[16] K. Thonicke, S. Venevsky, S. Sitch and W. Cramer, "The Role of Fire Disturbance for Global Vegetation Dynamics: Coupling Fire into a," *Global Ecology and Biogeography,* vol. 10, pp. 661-677, 2001.

[17] C. Black and et al, "Wildfire smoke exposure and human health: Significant gaps in research for a growing public health issue," *Environmental toxicology and pharmacology,* vol. 55, pp. 186-195, 2017.

For peer review only

[18] R. Rocha and A. A. Sant'Anna, "Winds of fire and smoke: Air pollution and health in the Brazilian Amazon," *World Development,* vol. 151, p. 105722, 2022.

[19] E. Grant and J. D. Runkle, "Long-term health effects of wildfire exposure: a scoping review," *The Journal of Climate Change and Health,* vol. 6, p. 100110, 2022.

[20] S. C. D. C. Xavier, A. L. R. Roque, V. D. S. Lima, K. J. L. Monteiro, J. C. R. Otaviano, L. F. C. Ferreira da Silva and A. M. Jansen, "Lower richness of small wild mammal species and Chagas disease risk," *PLoS neglected tropical diseases,* vol. 6, no. 5, p. e1647.

[21] D. Lawrence and K. Vandecar, "Effects of tropical deforestation on climate and agriculture," *Nature climate change,* vol. 5, no. 1, pp. 27-36, 2015.

[22] P. Alliance, "Effects of Deforestation," [Online]. Available: https://pachamama.org/effects-of-deforestation. [Accessed 05 Mar 2024].

[23] B. F. A. d. Oliveira, M. J. Bottino, P. Nobre and C. A. Nobre, "Deforestation and climate change are projected to increase heat stress risk in the Brazilian Amazon," *COMMUNICATIONS EARTH & ENVIRONMENT,* 2021.

[24] R. Becerril-Piña and C. A. Mastachi-Loza, "Desertification: Causes and Countermeasures," in *Life on land*, Springer, 2020.

[25] E. Nanjappan, E. Sullo, S. Shrestha, S. Thomas and E. Nouvet, "Californian Wildfires and Associated Human Health Outcomes: An Epidemiological Scoping Review," *International Journal of Trend in Scientific Research and Development,* vol. 5, no. 5, pp. 944-953, 2021.

[26] P. To, E. Eboreime and V. I. Agyapong, "The impact of wildfires on mental health: a scoping review," *Behavioral Sciences,* vol. 11, no. 9, p. 126, 2021.

[27] C. C. Melton, C. M. Fries, R. M. Smith and L. R. Mason, "Wildfires and Older Adults: A Scoping Review of Impacts, Risks, and Interventions," *International journal of environmental research and public health,* vol. 20, no. 13, p. 6252, 2023.

[28] C. E. Reid, M. Brauer, F. H. Johnston, M. Jerrett, J. R. Balmes and C. T. Elliott, "Critical review of health impacts of wildfire smoke exposure," *Environmental health perspectives,* vol. 124, no. 9, pp. 1334-1343, 2016.

[29] T. M. Davey and L. A. Selvey, "Relationship between Land Use/Land-Use Change and Human Health in Australia: A Scoping Study," *International Journal of Environmental Research and Public Health,* vol. 17, 2020.

[30] M. Mastel, A. Bussalleu, V. A. Paz-Soldán, G. Salmón-Mulanovich, A. Valdés-Velásquez and S. M. Hartinger, "Critical linkages between land use change and human health in the Amazon region: A scoping review," *PloS one,* vol. 13, no. 6, p. e0196414, 2018.

[31] R. Leichenko and J. A. Silva, "Climate change and poverty: vulnerability, impacts, and alleviation strategies," *WIREs Climate Change,* vol. 5, 2014.

[32] S. Hallegatte, M. Fay and E. B. Barbier, "Poverty and climate change: introduction," *Environment and Development Economics,* vol. 23, 2018.

For peer review only

[33] R. Mendelsohn, A. Dinar and L. Williams, “The distributional impact of climate change on rich and poor countries,” *Environment and Development Economics,* vol. 11, pp. 159-178, 2006.

[34] R. Davis, G. R. McGregor and K. B. Enfield, “Humidity: A review and primer on atmospheric moisture and human health,” *Environmental research,* vol. 144, pp. 106-116, 2016.

[35] J. Gao, Y. Sun, Y. Lu and L. Li, “Impact of ambient humidity on child health: a systematic review,” *PloS one,* vol. 9, no. 12, p. e112508, 2014.

[36] M. Osborne, “Wildfires Reached a Five-Year High in the Brazilian Amazon,” Smithsonian Magazine, 9 September 2022. [Online]. Available: https://www.smithsonianmag.com/smart-news/wildfires-reached-a-five-year-high-in-the-brazilian-amazon-180980719/. [Accessed 31 October 2023].

[37] G. Alecrim, “Número de queimadas na Amazônia em 2022 supera 2021, mas é inferior ao recorde de 2004,” CNN Brasil, 20 September 2022. [Online]. Available: https://www.cnnbrasil.com.br/nacional/numero-de-queimadas-na-amazonia-em-2022-supera-2021-mas-e-inferior-ao-recorde-de-2004. [Accessed 2023 October 31].

[38] N. Kshetri, D. C. Rojas Torres, H. Besada and M. A. Moros Ochoa, “Big data as a tool to monitor and deter environmental offenders in the global south: A multiple case study,” *Sustainability,* vol. 12, no. 24, p. 10436, 2020.

[39] M. D. Peters, C. M. Godfrey, H. Khalil, P. McInerney, D. Parker and C. B. Soares, “Guidance for conducting systematic scoping reviews,” *JBI Evidence Implementation,* vol. 13, no. 3, pp. 141-146, 2015.

[40] H. Arksey and L. O'Malley, “Scoping studies: towards a methodological framework,” *International journal of social research methodology,* vol. 8, no. 1, pp. 19-32, 2005.

[41] D. Levac, H. Colquhoun and K. K. O'Brien, “Scoping studies: advancing the methodology,” *mplementation science,* vol. 5, pp. 1-9, 2010.

[42] A. C. Tricco, E. Lillie, W. Zarin, K. K. O'Brien, H. Colquhoun, D. Levac, ... and S. E. Straus, “PRISMA extension for scoping reviews (PRISMA-ScR): checklist and explanation,” *Annals of internal medicine,* vol. 169, no. 7, pp. 467-473, 2018.

[43] G. Casais, N. S. Guimarães, T. R. Cortes, J. Pescarini, P. R. d. Magalhães, V. Wells, J. F. d. Sousa Filho, D. J. D. Neves, M. Shimonovich, J. R. Olsen, E. M. d. Carvalho Neto, P. J. Cooper, S. V. Katikireddi, R. Andrade, G. F. d. Santos and M. L. Barreto, “Wildfire, deforestation, and health in tropical rainforest: a scoping review protocol,” 2023. [Online]. Available: https://osf.io/pnqc7/.

[44] Nasa Earth Observatory, “Rainforest,” [Online]. Available: https://earthobservatory.nasa.gov/biome/biorainforest.php.

[45] D. M. Olson, E. Dinerstein, N. D. Wikramanayake, ... and K. Kassem, “Terrestrial ecoregions of the world: A new map of life on Earth,” *BioScience,* vol. 51, no. 11, pp. 933-938, 2001.

For peer review only

For peer review only

Appendices

Appendix I – Literature search

MEDLINE/PubMed

("wildfires"[MeSH Terms] OR "wildfires"[Text Word] OR "Wildfire"[All Fields] OR "Wildland Fires"[All Fields] OR "Brush Fires"[All Fields] OR "Brush Fire"[All Fields] OR "Forest Fires"[All Fields] OR "fire forest"[All Fields] OR "fires forest"[All Fields] OR "Forest Fire"[All Fields] OR "Wild Fires"[All Fields] OR "Wild Fire"[All Fields] OR "Fires"[MeSH Terms] OR "Fires"[Text Word] OR "Fire"[All Fields] OR "fire outbreaks"[All Fields] OR "Deforestation"[All Fields] OR "Grassfire"[All Fields] OR "prescribed burn"[All Fields] OR "prescribed fire"[All Fields]) AND ("Tropical Climate"[MeSH Terms] OR "Tropical Climate"[Text Word] OR "climate tropical"[All Fields] OR "climates tropical"[All Fields] OR "Tropical Climates"[All Fields] OR "Rainforest"[MeSH Terms] OR "Rainforest"[Text Word] OR "Rainforests"[All Fields] OR "Rain Forest"[All Fields] OR "forest rain"[All Fields] OR "Rain Forests"[All Fields] OR "Tropical Rainforest"[All Fields] OR "rainforest tropical"[All Fields] OR "rainforests tropical"[All Fields] OR "Tropical Rainforests"[All Fields] OR "Amazon"[All Fields] OR "Brazil"[All Fields] OR "Argentina"[All Fields] OR "Peru"[All Fields] OR "Ecuador"[All Fields] OR "Bolivia"[All Fields] OR "Colombia"[All Fields] OR "Venezuela"[All Fields] OR "Guyana"[All Fields] OR "Suriname"[All Fields] OR "French Guiana"[All Fields] OR "Paraguay"[All Fields] OR "Panama"[All Fields] OR "El Salvador"[All Fields] OR "Belize"[All Fields] OR "Costa Rica"[All Fields] OR "El Salvador"[All Fields] OR "Guatemala"[All Fields] OR "Honduras"[All Fields] OR "Nicaragua"[All Fields] OR "Panama"[All Fields] OR "Anguilla"[All Fields] OR "Antigua and Barbuda"[All Fields] OR "Aruba"[All Fields] OR "Bahamas"[All Fields] OR "Barbados"[All Fields] OR "British Virgin Islands"[All Fields] OR "Cayman Islands"[All Fields] OR "Cuba"[All Fields] OR "Dominica"[All Fields] OR "Dominican Republic"[All Fields] OR "Grenada"[All Fields] OR "Guadeloupe"[All Fields] OR "Haiti"[All Fields] OR "Jamaica"[All Fields] OR "Martinique"[All Fields] OR "Montserrat"[All Fields] OR "Netherlands Antilles"[All Fields] OR "Puerto Rico"[All Fields] OR "Saint Barthelemy"[All Fields] OR "Saint Kits and Nevis"[All Fields] OR "Saint Lucia"[All Fields] OR "Saint Martin"[All Fields] OR "Saint Vincent and the Grenadines"[All Fields] OR "Trinidad and Tobago"[All Fields] OR "United States Virgin Islands"[All Fields] OR "Mexico"[All Fields] OR "Hawaii"[All Fields] OR "Cape Verde"[All Fields] OR "Sao Tome and Principe"[All Fields] OR "Gambia"[All Fields] OR "Senegal"[All Fields] OR "Guinea-Bissau"[All Fields] OR "Guinea"[All Fields] OR "Sierra Leone"[All Fields] OR "Liberia"[All Fields] OR "Ivory Coast"[All Fields] OR "Ghana"[All Fields] OR "Togo"[All Fields] OR "Benin"[All Fields] OR "Nigeria"[All Fields] OR "Cameroon"[All Fields] OR "Central African Republic"[All Fields] OR "South Sudan"[All Fields] OR "Ethiopia"[All Fields] OR "Equatorial Guinea"[All Fields] OR "Gabon"[All Fields] OR "Congo"[All Fields] OR "Democratic Republic of the Congo"[All Fields] OR "Uganda"[All Fields] OR "Rwanda"[All Fields] OR "Burundi"[All Fields] OR "Kenya"[All Fields] OR "Somalia"[All Fields] OR "Tanzania"[All Fields] OR "Zambia"[All Fields] OR "Mozambique"[All Fields] OR "Madagascar"[All Fields] OR "Seychelles"[All Fields] OR "Mauritius"[All Fields] OR "Comoros"[All Fields] OR "Botswana"[All Fields] OR "Malawi"[All Fields] OR "Brunei"[All Fields] OR "Burma"[All Fields] OR "Myanmar"[All Fields] OR "Cambodia"[All Fields] OR "East Timor"[All Fields] OR

For peer review only

"Indonesia"[All Fields] OR "Laos"[All Fields] OR "Malaysia"[All Fields] OR "Philippines"[All Fields] OR "Singapore"[All Fields] OR "Thailand"[All Fields] OR "Vietnam"[All Fields] OR "India"[All Fields] OR "Papua New Guinea"[All Fields] OR "Australia"[All Fields] OR "Solomon Islands"[All Fields] OR "Vanuatu"[All Fields] OR "New Caledonia"[All Fields]) AND ("cardiovascular diseases"[MeSH Terms] OR "cardiovascular diseases"[Text Word] OR "Cardiovascular Disease"[All Fields] OR "disease cardiovascular"[All Fields] OR "Major Adverse Cardiac Events"[All Fields] OR "Cardiac Events"[All Fields] OR "Cardiac Event"[All Fields] OR "event cardiac"[All Fields] OR "Adverse Cardiac Event"[All Fields] OR "Adverse Cardiac Events"[All Fields] OR "cardiac events adverse"[All Fields] OR "chemically induced disorders"[MeSH Terms] OR "chemically induced disorders"[Text Word] OR "chemically induced disorders"[All Fields] OR "Chemically-Induced Disorder"[All Fields] OR "congenital, hereditary, and neonatal diseases and abnormalities"[MeSH Terms] OR "congenital hereditary and neonatal diseases and abnormalities"[Text Word] OR "Congenital Disorders"[All Fields] OR "disorder congenital"[All Fields] OR "disorders congenital"[All Fields] OR "Neonatal Diseases and Abnormalities"[All Fields] OR "Digestive System Diseases"[MeSH Terms] OR "Digestive System Diseases"[Text Word] OR "Digestive System Disease"[All Fields] OR "Digestive System Disorders"[All Fields] OR "Digestive System Disorder"[All Fields] OR "system disorders digestive"[All Fields] OR "Hepatobiliary Disorders"[All Fields] OR "Hepatobiliary Disorder"[All Fields] OR "Hepatobiliary Diseases"[All Fields] OR "Hepatobiliary Disease"[All Fields] OR "Disorders of Environmental Origin"[MeSH Terms] OR "Disorders of Environmental Origin"[Text Word] OR "Endocrine System Diseases"[MeSH Terms] OR "Endocrine System Diseases"[Text Word] OR "disease endocrine system"[All Fields] OR "diseases endocrine system"[All Fields] OR "Endocrine System Disease"[All Fields] OR "system disease endocrine"[All Fields] OR "system diseases endocrine"[All Fields] OR "Endocrine Diseases"[All Fields] OR "disease endocrine"[All Fields] OR "diseases endocrine"[All Fields] OR "Endocrine Disease"[All Fields] OR "Diseases of Endocrine System"[All Fields] OR "Eye Diseases"[MeSH Terms] OR "Eye Diseases"[Text Word] OR "Eye Disease"[All Fields] OR "Eye Disorders"[All Fields] OR "Eye Disorder"[All Fields] OR "Hemic and Lymphatic Diseases"[MeSH Terms] OR "Hemic and Lymphatic Diseases"[Text Word] OR "Blood and Lymphatic System Disorders"[All Fields] OR "Immune System Diseases"[MeSH Terms] OR "Immune System Diseases"[Text Word] OR "disease immune system"[All Fields] OR "Immune System Disease"[All Fields] OR "Immunologic Diseases"[All Fields] OR "disease immunologic"[All Fields] OR "Immunologic Disease"[All Fields] OR "Immunological Diseases"[All Fields] OR "disease immunological"[All Fields] OR "Immunological Disease"[All Fields] OR "Immune Diseases"[All Fields] OR "disease immune"[All Fields] OR "Immune Disease"[All Fields] OR "Diseases of Immune System"[All Fields] OR "Immune Disorders"[All Fields] OR "Immune Disorder"[All Fields] OR "Immune System Disorders"[All Fields] OR "disorder immune system"[All Fields] OR "Immune System Disorder"[All Fields] OR "Infections"[MeSH Terms] OR "Infections"[Text Word] OR "Infection and Infestation"[All Fields] OR "Infestation and Infection"[All Fields] OR "Infections and Infestations"[All Fields] OR "Infestations and Infections"[All Fields] OR "Infection"[All Fields] OR "Musculoskeletal Diseases"[MeSH Terms] OR "Musculoskeletal Diseases"[Text Word] OR "Musculoskeletal Disease"[All Fields] OR "Orthopedic Disorders"[All Fields] OR "Orthopedic Disorder"[All Fields] OR "Neoplasms"[MeSH Terms] OR "Neoplasms"[Text Word] OR "Tumor"[All Fields] OR "Neoplasm"[All Fields] OR "Tumors"[All Fields] OR "Neoplasia"[All Fields] OR "Neoplasias"[All Fields] OR "Cancer"[All Fields] OR "Cancers"[All Fields] OR "Malignant Neoplasm"[All Fields] OR "Malignancy"[All Fields] OR "Malignancies"[All Fields] OR "Malignant Neoplasms"[All Fields] OR "neoplasm malignant"[All Fields] OR "neoplasms malignant"[All Fields] OR "Benign

For peer review only

Neoplasms"[All Fields] OR "Benign Neoplasm"[All Fields] OR "neoplasms benign"[All Fields] OR "neoplasm benign"[All Fields] OR "Nervous System Diseases"[MeSH Terms] OR "Nervous System Diseases"[Text Word] OR "disease nervous system"[All Fields] OR "diseases nervous system"[All Fields] OR "Nervous System Disease"[All Fields] OR "Neurologic Disorders"[All Fields] OR "disorder neurologic"[All Fields] OR "disorders neurologic"[All Fields] OR "Neurologic Disorder"[All Fields] OR "Neurological Disorders"[All Fields] OR "disorder neurological"[All Fields] OR "disorders neurological"[All Fields] OR "Neurological Disorder"[All Fields] OR "Nervous System Disorders"[All Fields] OR "disorder nervous system"[All Fields] OR "disorders nervous system"[All Fields] OR "Nervous System Disorder"[All Fields] OR "Nutritional and Metabolic Diseases"[MeSH Terms] OR "Nutritional and Metabolic Diseases"[Text Word] OR "Occupational Diseases"[MeSH Terms] OR "Occupational Diseases"[Text Word] OR "disease occupational"[All Fields] OR "Occupational Disease"[All Fields] OR "Occupational Illnesses"[All Fields] OR "illnesses occupational"[All Fields] OR "diseases occupational"[All Fields] OR "Otorhinolaryngologic Diseases"[MeSH Terms] OR "Otorhinolaryngologic Diseases"[Text Word] OR "Otorhinolaryngological Disease"[All Fields] OR "Otorhinolaryngological Diseases"[All Fields] OR "Otolaryngological Diseases"[All Fields] OR "diseases otolaryngological"[All Fields] OR "Otolaryngological Disease"[All Fields] OR "Otorhinolaryngologic Disease"[All Fields] OR "ENT Diseases"[All Fields] OR "disease ent"[All Fields] OR "diseases ent"[All Fields] OR "ENT Disease"[All Fields] OR "Otolaryngologic Diseases"[All Fields] OR "disease otolaryngologic"[All Fields] OR "diseases otolaryngologic"[All Fields] OR "Otolaryngologic Disease"[All Fields] OR "pathological conditions, signs and symptoms"[MeSH Terms] OR "pathological conditions signs and symptoms"[Text Word] OR "Respiratory Tract Diseases"[MeSH Terms] OR "Respiratory Tract Diseases"[Text Word] OR "disease respiratory tract"[All Fields] OR "Respiratory Tract Disease"[All Fields] OR "Respiratory Diseases"[All Fields] OR "Respiratory System Diseases"[All Fields] OR "disease respiratory system"[All Fields] OR "Respiratory System Disease"[All Fields] OR "Skin and Connective Tissue Diseases"[MeSH Terms] OR "Skin and Connective Tissue Diseases"[Text Word] OR "Stomatognathic Diseases"[MeSH Terms] OR "Stomatognathic Diseases"[Text Word] OR "Stomatognathic Disease"[All Fields] OR "Mouth and Tooth Diseases"[All Fields] OR "Dental Diseases"[All Fields] OR "Dental Disease"[All Fields] OR "disease dental"[All Fields] OR "diseases dental"[All Fields] OR "Urogenital Diseases"[MeSH Terms] OR "Urogenital Diseases"[Text Word] OR "disease urogenital"[All Fields] OR "Urogenital Disease"[All Fields] OR "Genitourinary Diseases"[All Fields] OR "disease genitourinary"[All Fields] OR "Genitourinary Disease"[All Fields] OR "Wounds and Injuries"[MeSH Terms] OR "Wounds and Injuries"[Text Word] OR "Injuries and Wounds"[All Fields] OR "Wounds and Injury"[All Fields] OR "Injury and Wounds"[All Fields] OR "wounds injury"[All Fields] OR "Trauma"[All Fields] OR "Traumas"[All Fields] OR "injuries wounds"[All Fields] OR "research related injuries"[All Fields] OR "research related injuries"[All Fields] OR "Research-Related Injury"[All Fields] OR "Injuries"[All Fields] OR "Injury"[All Fields] OR "Wounds"[All Fields] OR "Wound"[All Fields] OR "Vital Statistics"[MeSH Terms] OR "Vital Statistics"[Text Word] OR "statistics vital"[All Fields] OR "Vital Statistics Registration"[All Fields] OR "Registration of Vital Statistics"[All Fields] OR "Vital Statistics Registrations"[All Fields] OR "registration vital statistics"[All Fields] OR "registrations vital statistics"[All Fields] OR "Patient Care"[MeSH Terms] OR "Patient Care"[Text Word] OR "care patient"[All Fields] OR "Informal care"[All Fields] OR "Informal cares"[All Fields] OR "care informal"[All Fields])

Date of search: August 18, 2023

For peer review only

Results: 2,296

Scopus

Search: ("wildfires" OR "wildfire" OR "wildland fires" OR "brush fires" OR "brush fire" OR "forest fires" OR "fire forest" OR "fires forest" OR "forest fire" OR "wild fires" OR "wild fire" OR "fires" OR "fire" OR "fire outbreaks" OR "deforestation" OR "grassfire" OR "prescribed burn" OR "prescribed fire")AND ("tropical climate" OR "climate tropical" OR "climates tropical" OR "tropical climates" OR "rainforest" OR "rainforests" OR "rain forest" OR "forest rain" OR "rain forests" OR "tropical rainforest" OR "rainforest tropical" OR "rainforests tropical" OR "tropical rainforests" OR "Amazon" OR "Brazil" OR "Argentina" OR "Peru" OR "Ecuador" OR "Bolivia" OR "Colombia" OR "Venezuela" OR "Guyana" OR "Suriname" OR "French Guiana" OR "Paraguay" OR "Panama" OR "El Salvador" OR "Belize" OR "Costa Rica" OR "El Salvador" OR "Guatemala" OR "Honduras" OR "Nicaragua" OR "Panama" OR "Anguilla" OR "Antigua and Barbuda" OR "Aruba" OR "Bahamas" OR "Barbados" OR "British Virgin Islands" OR "Cayman Islands" OR "Cuba" OR "Dominica" OR "Dominican Republic" OR "Grenada" OR "Guadeloupe" OR "Haiti" OR "Jamaica" OR "Martinique" OR "Montserrat" OR "Netherlands Antilles" OR "Puerto Rico" OR "Saint Barthelemy" OR "Saint Kits and Nevis" OR "Saint Lucia" OR "Saint Martin" OR "Saint Vincent and the Grenadines" OR "Trinidad and Tobago" OR "United States Virgin Islands" OR "Mexico" OR "Hawaii" OR "Cape Verde" OR "Sao Tome and Principe" OR "Gambia" OR "Senegal" OR "Guinea-Bissau" OR "Guinea" OR "Sierra Leone" OR "Liberia" OR "Ivory Coast" OR "Ghana" OR "Togo" OR "Benin" OR "Nigeria" OR "Cameroon" OR "Central African Republic" OR "South Sudan" OR "Ethiopia" OR "Equatorial Guinea" OR "Gabon" OR "Congo" OR "Democratic Republic of the Congo" OR "Uganda" OR "Rwanda" OR "Burundi" OR "Kenya" OR "Somalia" OR "Tanzania" OR "Zambia" OR "Mozambique" OR "Madagascar" OR "Seychelles" OR "Mauritius" OR "Comoros" OR "Botswana" OR "Malawi" OR "Brunei" OR "Burma" OR "Myanmar" OR "Cambodia" OR "East Timor" OR "Indonesia" OR "Laos" OR "Malaysia" OR "Philippines" OR "Singapore" OR "Thailand" OR "Vietnam" OR "India" OR "Papua New Guinea" OR "Australia" OR "Solomon Islands" OR "Vanuatu" OR "New Caledonia") AND ("cardiovascular diseases" OR "Cardiovascular Disease" OR "disease cardiovascular" OR "Major Adverse Cardiac Events" OR "Cardiac Events" OR "Cardiac Event" OR "event cardiac" OR "Adverse Cardiac Event" OR "Adverse Cardiac Events" OR "cardiac events adverse" OR "chemically induced disorders" OR "chemically induced disorders" OR "Chemically-Induced Disorder" OR "congenital, hereditary, and neonatal diseases and abnormalities" OR "Congenital Disorders" OR "disorder congenital" OR "disorders congenital" OR "Neonatal Diseases and Abnormalities" OR "Digestive System Diseases" OR "Digestive System Disease" OR "Digestive System Disorders" OR "Digestive System Disorder" OR "system disorders digestive" OR "Hepatobiliary Disorders" OR "Hepatobiliary Disorder" OR "Hepatobiliary Diseases" OR "Hepatobiliary Disease" OR "Disorders of Environmental Origin"OR "Endocrine System Diseases" OR "disease endocrine system" OR "diseases endocrine system" OR "Endocrine System Disease" OR "system disease endocrine" OR "system diseases endocrine" OR "Endocrine Diseases" OR "disease endocrine" OR "diseases endocrine" OR "Endocrine Disease" OR "Diseases of Endocrine System" OR "Eye Diseases" OR "Eye Disease" OR "Eye Disorders" OR "Eye Disorder" OR "Hemic and Lymphatic Diseases" OR "Blood and Lymphatic System Disorders" OR "Immune System Diseases" OR "disease immune system" OR "Immune System Disease" OR "Immunologic Diseases" OR "disease immunologic" OR "Immunologic Disease" OR "Immunological Diseases"

For peer review only

OR "disease immunological" OR "Immunological Disease" OR "Immune Diseases" OR "disease immune" OR "Immune Disease" OR "Diseases of Immune System" OR "Immune Disorders" OR "Immune Disorder" OR "Immune System Disorders" OR "disorder immune system" OR "Immune System Disorder" OR "Infections" OR "Infection and Infestation" OR "Infestation and Infection" OR "Infections and Infestations" OR "Infestations and Infections" OR "Infection" OR "Musculoskeletal Diseases" OR "Musculoskeletal Disease" OR "Orthopedic Disorders" OR "Orthopedic Disorder" OR "Neoplasms" OR "Tumor" OR "Neoplasm" OR "Tumors" OR "Neoplasia" OR "Neoplasias" OR "Cancer" OR "Cancers" OR "Malignant Neoplasm" OR "Malignancy" OR "Malignancies" OR "Malignant Neoplasms" OR "neoplasm malignant" OR "neoplasms malignant" OR "Benign Neoplasms" OR "Benign Neoplasm" OR "neoplasms benign" OR "neoplasm benign" OR "Nervous System Diseases" OR "disease nervous system" OR "diseases nervous system" OR "Nervous System Disease" OR "Neurologic Disorders" OR "disorder neurologic" OR "disorders neurologic" OR "Neurologic Disorder" OR "Neurological Disorders" OR "disorder neurological" OR "disorders neurological" OR "Neurological Disorder" OR "Nervous System Disorders" OR "disorder nervous system" OR "disorders nervous system" OR "Nervous System Disorder" OR "Nutritional and Metabolic Diseases" OR "Occupational Diseases" OR "disease occupational" OR "Occupational Disease" OR "Occupational Illnesses" OR "illnesses occupational" OR "diseases occupational" OR "Otorhinolaryngologic Diseases" OR "Otorhinolaryngological Disease" OR "Otorhinolaryngological Diseases" OR "Otolaryngological Diseases" OR "diseases otolaryngological" OR "Otolaryngological Disease" OR "Otorhinolaryngologic Disease" OR "ENT Diseases" OR "disease ent" OR "diseases ent" OR "ENT Disease" OR "Otolaryngologic Diseases" OR "disease otolaryngologic" OR "diseases otolaryngologic" OR "Otolaryngologic Disease" OR "pathological conditions, signs and symptoms" OR "Respiratory Tract Diseases" OR "disease respiratory tract" OR "Respiratory Tract Disease" OR "Respiratory Diseases" OR "Respiratory System Diseases" OR "disease respiratory system" OR "Respiratory System Disease" OR "Skin and Connective Tissue Diseases" OR "Stomatognathic Diseases" OR "Stomatognathic Disease" OR "Mouth and Tooth Diseases" OR "Dental Diseases" OR "Dental Disease" OR "disease dental" OR "diseases dental" OR "Urogenital Diseases" OR "disease urogenital" OR "Urogenital Disease" OR "Genitourinary Diseases" OR "disease genitourinary" OR "Genitourinary Disease" OR "Wounds and Injuries" OR "Injuries and Wounds" OR "Wounds and Injury" OR "Injury and Wounds" OR "wounds injury" OR "Trauma" OR "Traumas" OR "injuries wounds" OR "research related injuries" OR "research related injuries" OR "Research-Related Injury" OR "Injuries" OR "Injury" OR "Wounds" OR "Wound" OR "Vital Statistics" OR "statistics vital" OR "Vital Statistics Registration" OR "Registration of Vital Statistics" OR "Vital Statistics Registrations" OR "registration vital statistics" OR "registrations vital statistics" OR "Patient Care" OR "care patient" OR "Informal care" OR "Informal cares" OR "care informal")

Date of search: August 18, 2023

Results: 1,604

Biblioteca Virtual em Saúde

For peer review only - http://bmjopen.bmj.com/site/about/guidelines.xhtml

#1 "Incêndios Florestais" **OR** "Wildfires" OR "Incendios Forestales" OR "Incêndios" OR "Fires" OR "Incendios"

#2 "Clima Tropical" **OR** " Tropical Climate" **OR** "Floresta Úmida" OR "Rainforest" OR "Bosque Lluvioso" **OR** "Amazon" OR "Brazil" OR "Argentina" OR "Peru" OR "Ecuador" OR "Bolivia" OR "Colombia" OR "Venezuela" OR "Guyana" OR "Suriname" OR "French Guiana" OR "Paraguay" OR "Panama" OR "El Salvador" OR "Belize" OR "Costa Rica" OR "El Salvador" OR "Guatemala" OR "Honduras" OR "Nicaragua" OR "Panama" OR "Anguilla" OR "Antigua and Barbuda" OR "Aruba" OR "Bahamas" OR "Barbados" OR "British Virgin Islands" OR "Cayman Islands" OR "Cuba" OR "Dominica" OR "Dominican Republic" OR "Grenada" OR "Guadeloupe" OR "Haiti" OR "Jamaica" OR "Martinique" OR "Montserrat" OR "Netherlands Antilles" OR "Puerto Rico" OR "Saint Barthelemy" OR "Saint Kits and Nevis" OR "Saint Lucia" OR "Saint Martin" OR "Saint Vincent and the Grenadines" OR "Trinidad and Tobago" OR "United States Virgin Islands" OR "Mexico" OR "Hawaii" OR "Cape Verde" OR "Sao Tome and Principe" OR "Gambia" OR "Senegal" OR "Guinea-Bissau" OR "Guinea" OR "Sierra Leone" OR "Liberia" OR "Ivory Coast" OR "Ghana" OR "Togo" OR "Benin" OR "Nigeria" OR "Cameroon" OR "Central African Republic" OR "South Sudan" OR "Ethiopia" OR "Equatorial Guinea" OR "Gabon" OR "Congo" OR "Democratic Republic of the Congo" OR "Uganda" OR "Rwanda" OR "Burundi" OR "Kenya" OR "Somalia" OR "Tanzania" OR "Zambia" OR "Mozambique" OR "Madagascar" OR "Seychelles" OR "Mauritius" OR "Comoros" OR "Botswana" OR "Malawi" OR "Brunei" OR "Burma" OR "Myanmar" OR "Cambodia" OR "East Timor" OR "Indonesia" OR "Laos" OR "Malaysia" OR "Philippines" OR "Singapore" OR "Thailand" OR "Vietnam" OR "India" OR "Papua New Guinea" OR "Australia" OR "Solomon Islands" OR "Vanuatu" OR "New Caledonia"

#3 "Doenças Cardiovasculares" OR "Cardiovascular Diseases" OR "Enfermedades Cardiovasculares" OR "Distúrbios Induzidos Quimicamente" OR "Chemically-Induced Disorders" OR "Trastornos Químicamente Inducidos" OR "Doenças e Anormalidades Congênitas, Hereditárias e Neonatais" OR "Congenital, Hereditary, and Neonatal Diseases and Abnormalities" OR "Enfermedades y Anomalías Neonatales Congénitas y Hereditarias" OR "Doenças do Sistema Digestório" OR "Digestive System Diseases" OR "Transtornos de Origem Ambiental" OR "Disorders of Environmental Origin" OR "Trastornos de Origen Ambiental" OR "Doenças do Sistema Endócrino" OR "Endocrine System Diseases" OR "Enfermedades del Sistema Endocrino" OR "Oftalmopatias" OR "Eye Diseases" OR "Doenças Sanguíneas e Linfáticas" OR "Hemic and Lymphatic Diseases" OR "Enfermedades Hematológicas y Linfáticas" OR "Doenças do Sistema Imunitário" OR "Immune System Diseases" OR "Enfermedades del Sistema Inmune" OR "Infecções" OR "Infections" OR "Infecciones" OR "Doenças Musculoesqueléticas" OR "Musculoskeletal Diseases" OR "Enfermedades Musculoesqueléticas" OR "Neoplasias" OR "Neoplasms" OR "Doenças do Sistema Nervoso" OR "Nervous System Diseases" OR "Enfermedades del Sistema Nervioso" OR "Doenças Nutricionais e Metabólicas" OR "Nutritional and Metabolic Diseases" OR "Enfermedades Nutricionales y Metabólicas OR Doenças Profissionais" OR "Occupational Diseases" OR "Enfermedades Profesionales" OR "Otorrinolaringopatias" OR "Otorhinolaryngologic Diseases" OR "Enfermedades Otorrinolaringológicas" OR "Condições Patológicas, Sinais e Sintomas" OR "Pathological Conditions, Signs and Symptoms" OR "Condiciones Patológicas, Signos y Síntomas" OR "Doenças Respiratórias" OR "Respiratory Tract Diseases" OR "Enfermedades Respiratorias" OR "Doenças da Pele e do Tecido Conjuntivo" OR "Skin and Connective Tissue Diseases" OR "Enfermedades de la Piel y Tejido Conjuntivo" OR "Doenças Estomatognáticas" OR "Stomatognathic Diseases" OR "Enfermedades Estomatognáticas" OR "Doenças Urogenitais" OR "Urogenital Diseases" OR

"Enfermedades Urogenitales" OR "Ferimentos e Lesões" OR "Wounds and Injuries" OR "Heridas y Lesiones" OR "Estatísticas Vitais" OR "Vital Statistics" OR "Estadísticas Vitales" OR "Assistência ao Paciente" OR "Patient Care" OR "Atención al Paciente"

Detailed search:

("Incêndios Florestais" OR "Wildfires" OR "Incendios Forestales" OR "Incêndios" OR "Fires" OR "Incendios") AND ("Clima Tropical" OR " Tropical Climate" OR "Floresta Úmida" OR "Rainforest" OR "Bosque Lluvioso" OR "Amazon" OR "Brazil" OR "Argentina" OR "Peru" OR "Ecuador" OR "Bolivia" OR "Colombia" OR "Venezuela" OR "Guyana" OR "Suriname" OR "French Guiana" OR "Paraguay" OR "Panama" OR "El Salvador" OR "Belize" OR "Costa Rica" OR "El Salvador" OR "Guatemala" OR "Honduras" OR "Nicaragua" OR "Panama" OR "Anguilla" OR "Antigua and Barbuda" OR "Aruba" OR "Bahamas" OR "Barbados" OR "British Virgin Islands" OR "Cayman Islands" OR "Cuba" OR "Dominica" OR "Dominican Republic" OR "Grenada" OR "Guadeloupe" OR "Haiti" OR "Jamaica" OR "Martinique" OR "Montserrat" OR "Netherlands Antilles" OR "Puerto Rico" OR "Saint Barthelemy" OR "Saint Kits and Nevis" OR "Saint Lucia" OR "Saint Martin" OR "Saint Vincent and the Grenadines" OR "Trinidad and Tobago" OR "United States Virgin Islands" OR "Mexico" OR "Hawaii" OR "Cape Verde" OR "Sao Tome and Principe" OR "Gambia" OR "Senegal" OR "Guinea-Bissau" OR "Guinea" OR "Sierra Leone" OR "Liberia" OR "Ivory Coast" OR "Ghana" OR "Togo" OR "Benin" OR "Nigeria" OR "Cameroon" OR "Central African Republic" OR "South Sudan" OR "Ethiopia" OR "Equatorial Guinea" OR "Gabon" OR "Congo" OR "Democratic Republic of the Congo" OR "Uganda" OR "Rwanda" OR "Burundi" OR "Kenya" OR "Somalia" OR "Tanzania" OR "Zambia" OR "Mozambique" OR "Madagascar" OR "Seychelles" OR "Mauritius" OR "Comoros" OR "Botswana" OR "Malawi" OR "Brunei" OR "Burma" OR "Myanmar" OR "Cambodia" OR "East Timor" OR "Indonesia" OR "Laos" OR "Malaysia" OR "Philippines" OR "Singapore" OR "Thailand" OR "Vietnam" OR "India" OR "Papua New Guinea" OR "Australia" OR "Solomon Islands" OR "Vanuatu" OR "New Caledonia") AND ("Doenças Cardiovasculares" OR "Cardiovascular Diseases" OR "Enfermedades Cardiovasculares" OR "Distúrbios Induzidos Quimicamente" OR "Chemically-Induced Disorders" OR "Trastornos Químicamente Inducidos" OR "Doenças e Anormalidades Congênitas, Hereditárias e Neonatais" OR "Congenital, Hereditary, and Neonatal Diseases and Abnormalities" OR "Enfermedades y Anomalías Neonatales Congénitas y Hereditarias" OR "Doenças do Sistema Digestório" OR "Digestive System Diseases" OR "Transtornos de Origem Ambiental" OR "Disorders of Environmental Origin" OR "Trastornos de Origen Ambiental" OR "Doenças do Sistema Endócrino" OR "Endocrine System Diseases" OR "Enfermedades del Sistema Endocrino" OR "Oftalmopatias" OR "Eye Diseases" OR "Doenças Sanguíneas e Linfáticas" OR "Hemic and Lymphatic Diseases" OR "Enfermedades Hematológicas y Linfáticas" OR "Doenças do Sistema Imunitário" OR "Immune System Diseases" OR "Enfermedades del Sistema Inmune" OR "Infecções" OR "Infections" OR "Infecciones" OR "Doenças Musculoesqueléticas" OR "Musculoskeletal Diseases" OR "Enfermedades Musculoesqueléticas" OR "Neoplasias" OR "Neoplasms" OR "Doenças do Sistema Nervoso" OR "Nervous System Diseases" OR "Enfermedades del Sistema Nervioso" OR "Doenças Nutricionais e Metabólicas" OR "Nutritional and Metabolic Diseases" OR "Enfermedades Nutricionales y Metabólicas OR Doenças Profissionais" OR "Occupational Diseases" OR "Enfermedades Profesionales" OR "Otorrinolaringopatias" OR "Otorhinolaryngologic Diseases" OR "Enfermedades Otorrinolaringológicas" OR "Condições Patológicas, Sinais e Sintomas" OR

"Pathological Conditions, Signs and Symptoms" OR "Condiciones Patológicas, Signos y Síntomas" OR "Doenças Respiratórias" OR "Respiratory Tract Diseases" OR "Enfermedades Respiratorias" OR "Doenças da Pele e do Tecido Conjuntivo" OR "Skin and Connective Tissue Diseases" OR "Enfermedades de la Piel y Tejido Conjuntivo" OR "Doenças Estomatognáticas" OR "Stomatognathic Diseases" OR "Enfermedades Estomatognáticas" OR "Doenças Urogenitais" OR "Urogenital Diseases" OR "Enfermedades Urogenitales" OR "Ferimentos e Lesões" OR "Wounds and Injuries" OR "Heridas y Lesiones" OR "Estatísticas Vitais" OR "Vital Statistics" OR "Estadísticas Vitales" OR "Assistência ao Paciente" OR "Patient Care" OR "Atención al Paciente")

Date of search: August 18, 2023

Results: Lilacs (n = 44), Medline (n = 246), WPRIM (n = 3), BDENF – Enfermagem (n = 2), MINASPERÚ (n = 2), Recursos Multimídia (n = 2), BINACIS (n = 1), Desastres (n = 1), MedCarib (n = 1), PAHO-IRIS (n = 1), RDSM (n = 1), Coleciona SUS (n = 1).

Embase

#4

#1 AND #2 AND #3

4,019

#3

('wildfires'/syn OR 'fire'/syn OR 'deforestation'/syn) AND [embase]/lim

42,804

#2

('tropical climate'/syn OR 'climate tropical' OR 'climates tropical' OR 'tropical climates' OR 'rainforest'/syn OR 'rainforests' OR 'rain forest'/syn OR 'forest rain' OR 'rain forests' OR 'tropical rainforest'/syn OR 'rainforest tropical'/syn OR 'rainforests tropical' OR 'tropical rainforests' OR 'amazon'/syn OR 'brazil'/syn OR 'argentina'/syn OR 'peru'/syn OR 'ecuador'/syn OR 'bolivia'/syn OR 'colombia'/syn OR 'venezuela'/syn OR 'guyana'/syn OR 'suriname'/syn OR 'french guiana'/syn OR 'paraguay'/syn OR 'belize'/syn OR 'costa rica'/syn OR 'el salvador'/syn OR 'guatemala'/syn OR 'honduras'/syn OR 'nicaragua'/syn OR 'panama'/syn OR 'anguilla'/syn OR 'antigua and barbuda'/syn OR 'aruba'/syn OR 'bahamas'/syn OR 'barbados'/syn OR 'british virgin islands'/syn OR 'cayman islands'/syn OR 'cuba'/syn OR 'dominica'/syn OR 'dominican republic'/syn OR 'grenada'/syn OR 'guadeloupe'/syn OR 'haiti'/syn OR 'jamaica'/syn OR 'martinique'/syn OR 'montserrat'/syn OR 'netherlands antilles'/syn OR 'puerto rico'/syn OR 'saint barthelemy'/syn OR 'saint kits and nevis' OR 'saint lucia'/syn OR 'saint martin'/syn OR 'saint vincent and the grenadines'/syn OR 'trinidad and tobago'/syn OR 'united states virgin islands'/syn OR 'mexico'/syn OR 'hawaii'/syn OR 'cape verde'/syn OR 'sao tome and principe'/syn OR 'gambia'/syn OR 'senegal'/syn OR 'guinea-bissau'/syn OR 'guinea'/syn OR 'sierra leone'/syn OR 'liberia'/syn OR 'ivory coast'/syn OR 'ghana'/syn OR 'togo'/syn OR 'benin'/syn OR 'nigeria'/syn

OR 'cameroon'/syn OR 'central african republic'/syn OR 'south sudan'/syn OR 'ethiopia'/syn OR 'equatorial guinea'/syn OR 'gabon'/syn OR 'congo'/syn OR 'democratic republic of the congo'/syn OR 'uganda'/syn OR 'rwanda'/syn OR 'burundi'/syn OR 'kenya'/syn OR 'somalia'/syn OR 'tanzania'/syn OR 'zambia'/syn OR 'mozambique'/syn OR 'madagascar'/syn OR 'seychelles'/syn OR 'mauritius'/syn OR 'comoros'/syn OR 'botswana'/syn OR 'malawi'/syn OR 'brunei'/syn OR 'burma'/syn OR 'myanmar'/syn OR 'cambodia'/syn OR 'east timor'/syn OR 'indonesia'/syn OR 'laos'/syn OR 'malaysia'/syn OR 'philippines'/syn OR 'singapore'/syn OR 'thailand'/syn OR 'vietnam'/syn OR 'india'/syn OR 'papua new guinea'/syn OR 'australia'/syn OR 'solomon islands'/syn OR 'vanuatu'/syn OR 'new caledonia'/syn) AND [embase]/lim

4,621,441

#1

('patient care'/syn OR 'vital statistics'/syn OR 'wounds and injuries'/syn OR 'urogenital tract disease'/syn OR 'stomatognathic diseases'/syn OR 'skin and connective tissue diseases'/syn OR 'respiratory tract diseases'/syn OR 'pathological conditions, signs and symptoms'/syn OR 'otorhinolaryngologic diseases'/syn OR 'occupational diseases'/syn OR 'nutritional and metabolic diseases'/syn OR 'nervous system diseases'/syn OR 'neoplasms'/syn OR 'musculoskeletal disease'/syn OR 'infection'/syn OR 'immune system diseases'/syn OR 'hemic and lymphatic diseases'/syn OR 'eye diseases'/syn OR 'endocrine disease'/syn OR 'environmental disease'/syn OR 'digestive system diseases'/syn OR 'congenital, hereditary, and neonatal diseases and abnormalities'/syn OR 'chemically induced disorder'/syn OR 'cardiovascular diseases'/syn) AND [embase]/lim

Date of search: August 18, 2023

Records: 4,020

EconLit

(('wildfires' or 'fire' or 'deforestation') and ((((((((((('tropical climate' or 'climate tropical' or 'climates tropical' or 'tropical climates' or 'rainforest' or 'rainforests' or 'rain forest' or 'forest rain' or 'rain
forests' or 'tropical rainforest' or 'rainforest tropical' or 'rainforests tropical' or 'tropical rainforests' or 'Amazon' or 'Brazil' or 'Argentina' or 'Peru' or 'Ecuador' or 'Bolivia' or 'Colombia' or 'Venezuela' or 'Guyana' or
'Suriname' or 'French Guiana' or 'Paraguay' or 'Panama' or 'El Salvador' or 'Belize' or 'Costa Rica' or 'El Salvador' or
'Guatemala' or 'Honduras' or 'Nicaragua' or 'Panama' or 'Anguilla' or 'Antigua) and Barbuda') or 'Aruba' or 'Bahamas' or
'Barbados' or 'British Virgin Islands' or 'Cayman Islands' or 'Cuba' or 'Dominica' or 'Dominican Republic' or 'Grenada'
or 'Guadeloupe' or 'Haiti' or 'Jamaica' or 'Martinique' or 'Montserrat' or 'Netherlands Antilles' or 'Puerto Rico' or
'Saint Barthelemy' or 'Saint Kits) and Nevis') or 'Saint Lucia' or 'Saint Martin' or 'Saint Vincent) and the

For peer review only

Grenadines') or 'Trinidad) and Tobago') or 'United States Virgin Islands' or 'Mexico' or 'Hawaii' or 'Cape Verde' or 'Sao Tome) and Principe') or 'Gambia' or 'Senegal' or 'Guinea-Bissau' or 'Guinea' or 'Sierra Leone' or 'Liberia' or 'Ivory Coast' or 'Ghana' or 'Togo' or 'Benin' or 'Nigeria' or 'Cameroon' or 'Central African Republic' or 'South Sudan' or 'Ethiopia' or 'Equatorial Guinea' or 'Gabon' or 'Congo' or 'Democratic Republic of the Congo' or 'Uganda' or 'Rwanda' or 'Burundi' or 'Kenya' or 'Somalia' or 'Tanzania' or 'Zambia' or 'Mozambique' or 'Madagascar' or 'Seychelles' or 'Mauritius' or 'Comoros' or 'Botswana' or 'Malawi' or 'Brunei' or 'Burma' or 'Myanmar' or 'Cambodia' or 'East Timor' or 'Indonesia' or 'Laos' or 'Malaysia' or 'Philippines' or 'Singapore' or 'Thailand' or 'Vietnam' or 'India' or 'Papua New Guinea' or 'Australia' or 'Solomon Islands' or 'Vanuatu' or 'New Caledonia') and (((((((((((('patient care' or 'vital statistics' or 'wounds) and injuries') or 'urogenital tract disease' or 'stomatognathic diseases' or 'skin) and connective tissue diseases') or 'respiratory tract diseases' or 'pathological conditions, signs) and symptoms') or 'otorhinolaryngologic diseases' or 'occupational diseases' or 'nutritional) and metabolic diseases') or 'nervous system diseases' or 'neoplasms' or 'musculoskeletal disease' or 'infection' or 'immune system diseases' or 'hemic) and lymphatic diseases') or 'eye diseases' or 'endocrine disease' or 'environmental disease' or 'digestive system diseases' or 'congenital, hereditary,) and neonatal diseases and abnormalities') or 'chemically induced disorder' or 'cardiovascular diseases')).mp. [mp=tx, bt, ti, ab, ct, hw, id]

Date of search: August 18, 2023

Records: 464

For peer review only

Appendix II - Ongoing work

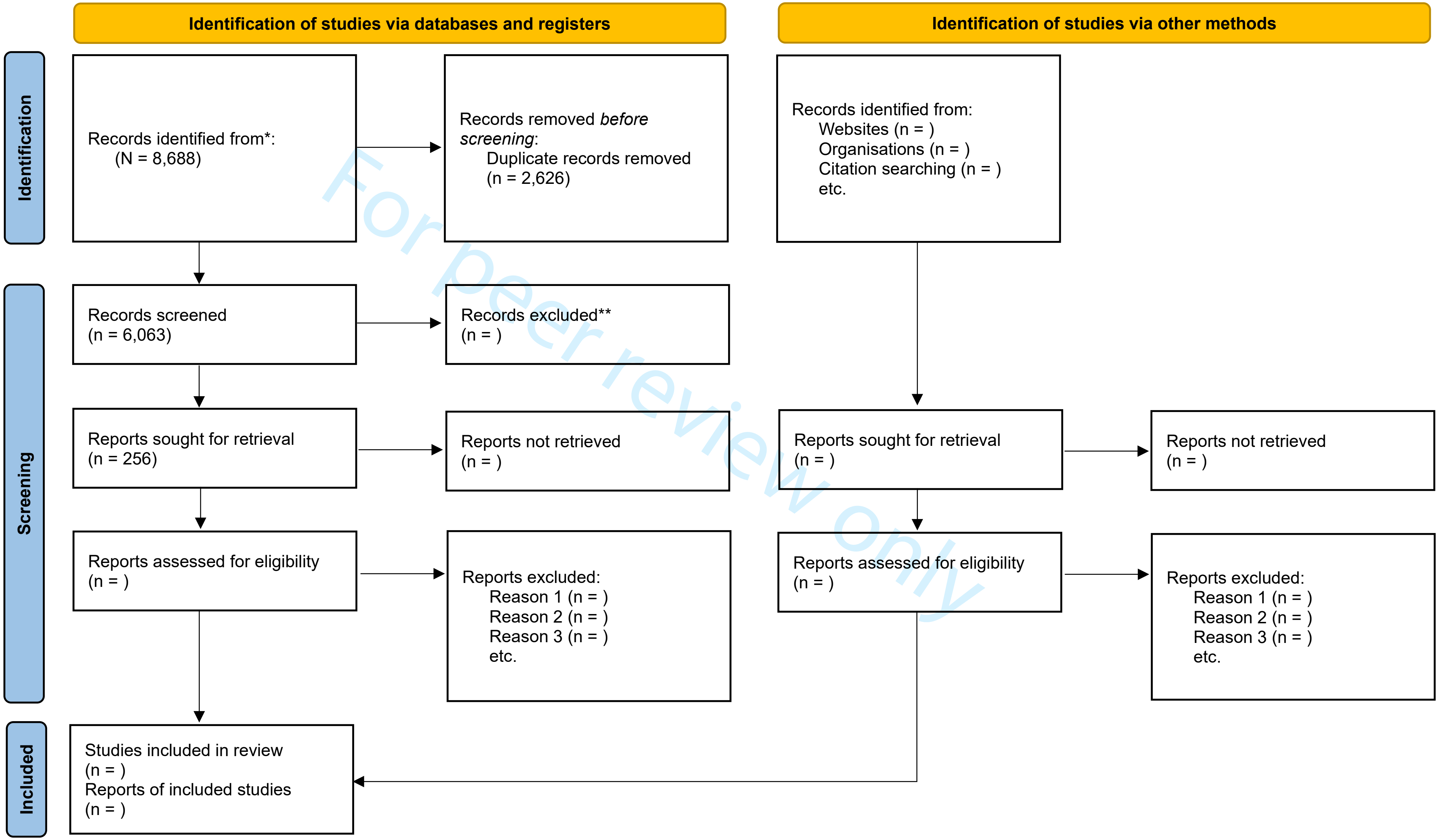

From: Page MJ, McKenzie JE, Bossuyt PM, Boutron I, Hoffmann TC, Mulrow CD, et al. The PRISMA 2020 statement: an updated guideline for reporting systematic reviews. BMJ 2021;372:n71. doi: 10.1136/bmj.n71.

For peer review only

Appendix III - Data extraction form

**General information**	
Reviewer who performed the data extraction:	
Date of the data extraction performed:	
Paper database:	
**Study identification**	
Publication full title:	
DOI number:	
First author name:	
Year of publication:	
Journal of publication:	
Aim of study:	
**Exposure and data source**	
Area (city, region, country):	
Exposure:	
Exposure temporal level:	
Exposure temporal duration:	
Origin of the exposure data source:	
Origin of the outcome data source:	
**Method**	
The statistical model applied:	
**Study design and findings**	
Study population:	
Health outcome:	
Type of disease:	
Lag between exposure and the health consequences	
Study findings as reported by the authors:	
Report the following list:<br>1. measure of association + standard errors for every different regression specification<br>2. Subgroups estimates<br>3. Confounders used<br>4. Identification strategy if appliable	
**Conclusions**	
Study limitations identified by the team:	
Policy recommendation from the study	

For peer review only

BMJ Open

Wildfire, deforestation, and health in tropical rainforest areas: a scoping review protocol

Journal:	*BMJ Open*
Manuscript ID	bmjopen-2023-082381.R2
Article Type:	Protocol
Date Submitted by the Author:	10-Apr-2024

Complete List of Authors:	Casais, Gustavo; Fiocruz Brasilia, Center of Data and and Knowledge Integration for Health<br>Guimarães, Nathalia ; Federal University of Minas Gerais, Departament of Nutrition<br>Cortes, Taísa ; Center of Data and Knowledge Integration for Health (CIDACS) - Fiocruz/BA, Center of Data and and Knowledge Integration for Health<br>Pescarini , Julia ; London School of Hygiene & Tropical Medicine; Center of Data and Knowledge Integration for Health (CIDACS)<br>Rebouças de Magalhães, Poliana; Center of Data and Knowledge Integration for Health (CIDACS) - Fiocruz/BA<br>Wells, Valerie; University of Glasgow<br>de Sousa Filho, José Firmino; Center of Data and Knowledge Integration for Health (CIDACS) - Fiocruz/BA<br>Delgado Neves, Danielson; Center of Data and Knowledge Integration for Health (CIDACS) - Fiocruz/BA<br>Shimonovich , Michal ; University of Glasgow<br>Olsen, Jonathan; University of Glasgow<br>de Carvalho Neto, Edgar Marcelino; Center of Data and Knowledge Integration for Health (CIDACS) - Fiocruz/BA<br>Cooper, Philip; Universidad Internacional del Ecuador; St George's, University of London<br>Katikireddi, Srinivasa; University of Glasgow<br>Emanuel, Lucas; Federal University of Bahia; Center of Data and Knowledge Integration for Health (CIDACS) - Fiocruz/BA<br>Andrade , Roberto F. S. ; Center of Data and Knowledge Integration for Health (CIDACS) - Fiocruz/BA; Federal University of Bahia<br>Ferreira dos Santos, Gervasio; Center of Data and Knowledge Integration for Health (CIDACS) - Fiocruz/BA; Federal University of Bahia<br>Barreto, Mauricio; Center of Data and Knowledge Integration for Health (CIDACS) - Fiocruz/BA; Federal University of Bahia

**Primary Subject Heading**:	Public health
Secondary Subject Heading:	Epidemiology, Global health, Health policy, Health economics
Keywords:	EPIDEMIOLOGY, PUBLIC HEALTH, STATISTICS & RESEARCH METHODS, TROPICAL MEDICINE

SCHOLARONE™
Manuscripts

Wildfire, deforestation, and health in tropical rainforest areas: a scoping review protocol

Gustavo Casais[1], Nathalia Sernizon Guimarães[2], Taísa Rodrigues Cortes[1], Julia Pescarini[1,3], Poliana Rebouças de Magalhães[1], Valerie Wells[4], José Firmino de Sousa Filho[1], Danielson Jorge Delgado Neves[1], Michal Shimonovich[4], Jonathan R Olsen[4], Edgar Marcelino de Carvalho Neto[1], Philip J. Cooper[5,6], Srinivasa Vittal Katikireddi[4], Lucas Emanuel[1,7], Roberto F. S. Andrade[1,7], Gervasio Ferreira dos Santos[1,7], Mauricio L. Barreto[1,7], on behalf of the Unit on the Social and Environmental Determinants of Health Inequalities (SEDHI)

[1]Center for Data and Knowledge Integration for Healthcare (CIDACS)

[2]Federal University of Minas Gerais

[3]London School of Hygiene and Tropical Medicine

[4]MRC/CSO Social and Public Health Sciences Unit, University of Glasgow

[5]St George's, University of London

[6]School of Medicine, Universidad Internacional del Ecuador, Quito, Ecuador

[7]Federal University of Bahia

Author address and contact details:

Gustavo Casais, PhD, Center of Data and Knowledge Integration for Health (CIDACS) - Fiocruz/BA, Parque Tecnológico da Bahia, Edf. Tecnocentro, R. Mundo, 121 - sala 315 - Trobogy, Salvador - BA, Brazil, 41745-715, gustavo.casais@hotmail.com, +55 (71) 99127 3345.

Nathalia Sernizon Guimarães, PhD, Professor at Departament of Nutrition, Federal University of Minas Gerais, Belo Horizonte, Minas Gerais, Brazil zip code 30130100.

Taísa Rodrigues Cortes, PhD, Center of Data and Knowledge Integration for Health (CIDACS) - Fiocruz/BA, Parque Tecnológico da Bahia, Edf. Tecnocentro, R. Mundo, 121 - sala 315 - Trobogy, Salvador - BA, Brazil, 41745-715, taisacortes@gmail.com, +55 (71) 99127 3345.

Julia Pescarini, PhD, London School of Hygiene and Tropical Medicine, Center of Data and and Knowledge Integration for Health (CIDACS) - Fiocruz/BA, Keppel Street, London, WC1E 7HT, United Kingdom, julia.pescarini1@lshtm.ac.uk, +44 (0)20 7636 8636.

Poliana Rebouças de Magalhães, PhD, Center of Data and Knowledge Integration for Health (CIDACS) - Fiocruz/BA, Parque Tecnológico da Bahia, Edf. Tecnocentro, R. Mundo, 121 - sala 315 - Trobogy, Salvador - BA, Brazil, 41745-715, poliana.reboucas@fiocruz.br, +55 (71) 99127 3345.

Valerie Wells, Ms, MRC/CSO Social and Public Health Sciences Unit, University of Glasgow. 90 Byres Road, Glasgow, G12 8TB, United Kingdom, Valerie.Wells@glasgow.ac.uk, +44 0141 330 4042.

For peer review only

José Firmino de Sousa Filho, PhD, Center of Data and Knowledge Integration for Health (CIDACS) - Fiocruz/BA, Parque Tecnológico da Bahia, Edf. Tecnocentro, R. Mundo, 121 - sala 315 - Trobogy, Salvador - BA, Brazil, 41745-715, jose.ffilho@fiocruz.br, +55 (71) 99127 3345.

Danielson Jorge Delgado Neves[1], PhD, Center of Data and Knowledge Integration for Health (CIDACS) - Fiocruz/BA, Parque Tecnológico da Bahia, Edf. Tecnocentro, R. Mundo, 121 - sala 315 - Trobogy, Salvador - BA, Brazil, 41745-715, danielson.neves@fiocruz.br, +55 (71) 99127 3345.

Michal Shimonovich, PhD, MRC/CSO Social & Public Health Sciences Unit, University of Glasgow, Berkeley Square, 99 Berkeley Street, Glasgow G3 7HR, United Kingdom, Michal.Shimonovich@glasgow.ac.uk, +44 0141 330 4042.

Jonathan R Olsen, PhD, MRC/CSO Social & Public Health Sciences Unit, University of Glasgow, Berkeley Square, 99 Berkeley Street, Glasgow G3 7HR, United Kingdom, Jonathan.Olsen@glasgow.ac.uk, +44 0141 330 4042.

Edgar Marcelino de Carvalho Neto, PhD, Center of Data and Knowledge Integration for Health (CIDACS) - Fiocruz/BA, Parque Tecnológico da Bahia, Edf. Tecnocentro, R. Mundo, 121 - sala 315 - Trobogy, Salvador - BA, Brazil, 41745-715, edgar.neto@fiocruz.br, +55 (71) 99127 3345.

Philip J. Cooper, PhD, St George's, University of London; School of Medicine, Universidad Internacional del Ecuador, Av. Simón Bolívar y Av. Jorge Fernández, Quito, Ecuador, pcooper@sgul.ac.uk, +593 2 2985 600.

Srinivasa Vittal Katikireddi, PhD, MRC/CSO Social & Public Health Sciences Unit, University of Glasgow, Berkeley Square, 99 Berkeley Street, Glasgow G3 7HR, United Kingdom, Vittal.Katikireddi@glasgow.ac.uk, +44 0141 330 4042.

Lucas Emanuel, Federal University of Bahia; Center of Data and Knowledge Integration for Health (CIDACS) - Fiocruz/BA, Parque Tecnológico da Bahia, Edf. Tecnocentro, R. Mundo, 121 - sala 315 - Trobogy, Salvador - BA, Brazil, 41745-715, lucasemanuel@ufba.br, +55 (71) 99127 3345.

Dr. Roberto Fernandes Silva Andrade, Federal University of Bahia; Center of Data Knowledge Integration for Health (CIDACS) - Fiocruz/BA, Parque Tecnológico da Bahia, Edf. Tecnocentro, R. Mundo, 121 - sala 315 - Trobogy, Salvador - BA, Brazil, 41745-715, randrade@ufba.br, +55 (71) 99127 3345.

Dr. Gervasio Ferreira dos Santos, Federal University of Bahia; Center of Data Knowledge Integration for Health (CIDACS) - Fiocruz/BA, Parque Tecnológico da Bahia, Edf. Tecnocentro, R. Mundo, 121 - sala 315 - Trobogy, Salvador - BA, Brazil, 41745-715, gervasios@ufba.br, +55 (71) 99127 3345.

Mauricio L. Barreto, Federal University of Bahia; Center of Data Knowledge Integration for Health (CIDACS) - Fiocruz/BA, Parque Tecnológico da Bahia, Edf. Tecnocentro, R. Mundo, 121 - sala 315 - Trobogy, Salvador - BA, Brazil, 41745-715, mauricio@ufba.br, +55 (71) 99127 3345.

Correspondence to:

For peer review only

Gustavo Casais, PhD, Center of Data and Knowledge Integration for Health (CIDACS) - Fiocruz/BA, Parque Tecnológico da Bahia, Edf. Tecnocentro, R. Mundo, 121 - sala 315 - Trobogy, Salvador - BA, Brazil, 41745-715, gustavo.casais@hotmail.com, +55 (71) 99127 3345.

Word count: 2,619

Keywords: Wildfire; Deforestation; Tropical Climate; Rainforest; Health

Abstract

Introduction: Wildfires and deforestation potentially have direct effects on multiple health outcomes as well as indirect consequences for climate change. Tropical rainforest areas are characterized by high rainfall, humidity, and temperature, and they are predominantly found in low- and middle-income countries. This study aims to synthesize the methods, data, and health outcomes reported in scientific papers on wildfires and deforestation in these locations.

Methods and analysis: We will carry out a scoping review according to the Joanna Briggs Institute's manual for scoping reviews and the framework proposed by Arksey and O'Malley, and Levac et al. The search for articles was performed on August 18, 2023, in 16 electronic databases using MeSH Terms and adaptations for each database from database inception. The search for local studies will be complemented by the manual search in the list of references of the studies selected to compose this review. We screened studies written in English, French, Portuguese and Spanish. We included quantitative studies assessing any human disease outcome, hospitalization, and vital statistics in regions of tropical rainforest. We exclude qualitative studies and quantitative studies whose outcomes do not cover those of interest. The text screening was done by two independent reviewers. Subsequently, we will tabulate the data by the origin of the data source used, the methods, and the main findings on health impacts the extracted data. The results will provide descriptive statistics, along with visual representations in diagrams and tables, complemented by narrative summaries as detailed in the JBI guidelines.

Ethics and dissemination: The study does not require an ethical review as it is meta-research and utilizes published, deidentified secondary data sources. The submission of results for publication in a peer-reviewed journal and presentation at scientific and policymakers' conferences is expected.

Study registration: OSF, https://osf.io/pnqc7/.

Strengths and limitations of this study

- This scoping review will assess the health impacts of wildfires and deforestation across a broad range of tropical rainforest regions.
- The search will include several different databases, including those from Latin America and Africa.
- It will include manuscripts in English, French, Portuguese and Spanish, from database inception.

For peer review only

- The ultimate selection of papers will be heavily influenced by the resolution of the shapefile used to delineate the tropical rainforest biome; this sensitivity to the map's resolution may result in the improper exclusion or inclusion of studies.
- A major limitation is the absence of critical appraisal, meaning that studies with potentially low relevance, reliability, validity and applicability might be included.

INTRODUCTION

One of the significant contributors to climate change is improper land use resulting from agriculture, logging, and mining, which potentially leads to wildfires and deforestation [1] [2]. Currently, wildfires and deforestation have been increasingly drawing attention for their potential consequences, not only for climate change but also for the health outcomes of both local and global populations. Governments across the world have been expressing this concern by adopting climate mitigation policies to contain environmental degradation. Understanding the health effects of wildfires and deforestation on populations in low- and middle-income countries is critical for designing evidence-based and successful mitigation plans and policies [3].

Tropical rainforests are home to not only a vast array of animal and plant species but also play a vital role in sustaining human well-being [4]. These ecosystems provide essential resources such as cocoa, coffee beans, bananas, vanilla, and cinnamon, which are used in everyday products. Furthermore, they are a rich source of chemical compounds instrumental in the development of medicines. These biomes are the ancestral homes of Indigenous and traditional peoples who not only live within these ecosystems but also actively preserve them along with their cultures. Rainforests are crucial in stabilizing the climate and maintaining the water cycle, contributing to the overall balance of global weather patterns [5] [6]. Without these rainforests, the dynamics and control of zoonotic diseases and vector-borne infections would be significantly disrupted [7]. The loss of rainforests would have severe consequences for the economy, global biodiversity and ecosystem services [8]. Figure 1 displays the location of all rainforest areas in the world according to [9].

While wildfires and deforestation often stem from common causes, they are also influenced by distinct drivers. Human activities, such as land clearing for agriculture, logging and development, play a significant role in both phenomena [10] [11]. In low- and middle-income countries, where agriculture constitutes a substantial portion of the economy, fluctuations in global demand can drive the expansion of agricultural areas, contributing to deforestation and increasing wildfire susceptibility [12] [13]. Additionally, inadequate policies, weak enforcement of regulations, and governance issues exacerbate both deforestation and wildfire risks by permitting unsustainable land use practices and inadequately allocating resources for fire prevention and suppression efforts [14]. Moreover, the

For peer review only

escalating frequency and intensity of extreme events, such as droughts, extreme temperatures, and storms, linked to climate change, have been amplifying wildfire occurrences globally over the past few decades [15]. Also, due to the usually high humidity in tropical rainforests, wildfires very rarely occur by natural causes, so that both deforestations and wildfires are much closely related to human activity [16] [17].

Wildfires have the potential to cause bodily injuries, impact housing infrastructure, and release toxic gases and particulate matter into the air [18]. Exposure to smoke from wildfires can cause acute respiratory illness and exacerbate existing disease, especially among children and elderly [19]. Additionally, the long-term effects of accumulated exposures to wildfires may be multiple including premature death, cardiovascular disease, cancer, respiratory illness, mental health, and other chronic conditions [20].

Deforestation has different causal mechanisms for public health outcomes. Deforestation can alter environmental niches, changing habitats for parasites and insects, including disease-carrying mosquitoes, which may increase the human risk of contracting vector-borne diseases such as malaria and dengue [21] [7] [22]. Additionally, in the long run, deforestation can reduce the level of water in the atmosphere, lead to soil erosion, desertification, flooding, and increase the local temperature [23] [24] [25].

We have noticed that previous reviews on the health impacts of wildfires and deforestation have some limitations. They often focus on specific population groups or a limited number of health outcomes, and they might not search comprehensively across databases or years [20] [26] [27] [28] [29] [30] [31]. Notably, no review has specifically addressed the health effects of wildfires in tropical rainforest areas, which are mainly found in low- and middle-income countries. These regions are home to many Indigenous and marginalized groups who are particularly vulnerable due to limited resources, higher risk of health problems, and limited access to healthcare [32] [33] [34]. Tropical climate, characterized by elevated temperatures, intense rainfall, and high humidity, can further impact health conditions [35] [36]. Moreover, the most recent reviews on the health effects of deforestation are at least four years old, indicating the need for an updated assessment [30] [31].

Conducting an updated review is crucial, especially considering the recent escalation of wildfires and deforestation. This issue is particularly significant in Brazil, where fire outbreaks have been steadily increasing, even though they have not reached the peak observed in 2004 [37] [38]. Such a review will help identify and understand the specific ways in which wildfires and deforestation affect health outcomes, hospitalizations, and vital statistics, the quantitative methods employed, and the data sources used for these analyses.

For peer review only

Other critical areas of investigation include geographical mapping and the analytical methods employed for analysis. There is a wide variety of information sources that provide mapping for the occurrence of wildfires and deforestation, differentiating based on geographical scale and the time period of change. Disparities in high-quality environmental data around the world highlight how some places possess better information than others, which can be considered part of the phenomenon known as the 'digital divide' [39]. Additionally, various analytical methods exist to study how wildfires and deforestation impact people's health. The outcomes of such analyses heavily rely on the modelling techniques employed, underscoring the crucial need to understand and utilize an array of available methods. This understanding is pivotal in creating thorough and accurate insights to inform the creation of future high-quality research and understand what key research gaps exist.

This scoping review aims to comprehensively synthesize the intricate relationships between wildfires, deforestation, and their impact on health outcomes in tropical rainforest regions. Our more specific objectives will be to characterize: (i) the health outcomes affected by wildfires and deforestation in the tropical areas; (ii) the methods used to identify and measure their impact; (iii) the data sources related to the wildfires and deforestation; (iv) and the policy recommendations from the studies. This will equip policymakers and researchers with essential information about this research area, highlighting knowledge gaps and paving the way for future research and development.

METHODS AND ANALYSIS

This scoping review protocol follows the guidelines of the Joanna Briggs Institute. The protocol is based on the framework suggested by Peters, et al [40], Arksey and O'Malley [41], and enhanced by Levac et al. [42]. It was written according to the checklist provided by the Preferred Reporting Items for Systematic Reviews and Meta-Analyses Extension for Scoping Reviews (PRISMA-ScR) [43].

The scoping review will follow the steps below: (1) identifying the research question; (2) identifying relevant studies; (3) study selection; (4) charting the data; (5) collating, summarising, and reporting the results. The scoping review protocol was previously registered with the Open Science Framework to identify ongoing reviews and avoid unnecessary duplication of research [44].

Step 1: Identifying the research question

To enhance the organization of our research question and the criteria for inclusion and exclusion we adhered to the mnemonic PCC (Population, Concept, Context), which is described with the research question in the Table 1.

According to the Oxford Dictionary, wildfire means: "a very big fire that spreads quickly and burns natural areas like woods, forests and grassland". We adopt this definition; therefore, we consider any

For peer review only - http://bmjopen.bmj.com/site/about/guidelines.xhtml

large fire that occur in different types of vegetation which can affect urban, peri urban or rural area. The deforestation is usually associated with human activity pursuing an economic purpose (e.g., farming, timber logging, expansion and infrastructure, and mining). The tropical rainforest is a warm and humid biome characterized by year-round rainfall. Renowned for its thick layers of vegetation, it consists of three distinct canopy levels located between the Tropic of Cancer and the Tropic of Capricorn [45]. We will consider the deforestation of the tropical forests located in urban, peri-urban, or rural areas.

Table 1. Scoping review questions and PCC mnemonic

Question	Population (P)	Concept (C)	Context (C)
1. What are the impacts of the wildfire and deforestation on the health outcomes, hospitalization, and vital statistics in the tropical rainforest areas?<br>2. What are the methods and data sources used for this assessment?	All individuals	Health impacts of wildfires and deforestation	All the areas located in the tropical rainforests

Step 2: Identifying relevant studies

Data sources

The search for scientific articles were conducted on August 18, 2023, across several databases: (1) Nursing Database (BDENF – Enfermagem), (2) National Bibliography in Argentine Health Sciences (BINACIS), (3) Coleciona SUS, (4) Desastres, (5) EconLit, (6) Embase, (7) Latin American and Caribbean Literature in Health Sciences (Lilacs), (8) Literature in Health Sciences from Caribbean countries (MedCaribe), (9) MEDLINE, (10) MEDLINE/PubMed, (11) Virtual Health Library of the Ministry of Health of Peru (MINSAPERU), (12) Literature from the Pan American Health Organization Headquarters Library (PAHO-IRIS), (13) Health Documentation Network in Mozambique (RDSM), (14) Recursos Multimídia, (15) Scopus, (16) Western Pacific Region Index Medicus (WPRIM). Access to Scopus database will be via the Capes Platform, while the databases corresponding to the previous identification number: (1), (2), (3), (4), (7), (8), (9), (11), (12), (13), (14), and (16) are available via the Virtual Health Library platform (Biblioteca Virtual da Saúde in Portuguese). To strengthen this review, we will also perform a manual search of the references in the included studies.

Table 2. Search terms by topics

For peer review only

1. "Wildfires"[MeSH Terms] OR "Deforestation"[All Fields]
2. 'All categories of human diseases' OR "Vital Statistics"[MeSH Terms] OR "Patient Care"[MeSH Terms]
3. "Rainforest"[MeSH Terms] OR 'All country names with tropical rainforest'
4. (1) AND (2) AND (3)

Note: The complete list of terms used can be found in the Appendix I.

Search strategy

The search strategy will be defined for each database, following the inclusion and exclusion criteria. The search terms were used according to the Medical Subject Headings (MeSH) and the respective Entry Terms. The expression terms were categorized into three broad aspects according to Table 2: (i) the exposure, including the wildfires and the deforestation terms, (ii) a comprehensive list of diseases, hospitalization, vital statistics terms, and (iii) tropical rainforest areas, including a list of countries with tropical rainforests. The countries that harbour tropical rainforests were selected according to two maps [45] [9]. The expression terms will be combined with the Boolean operators 'AND' and 'OR' in the refinement. The complete search terms in every database are in the Appendix I. In the non-PubMed indexing databases, we utilized Emtree (for searching in Embase) and Descritores em Ciências da Saúde (DeCS) (for searching in the Virtual Health Library platform). Additionally, we used jargon in the search terms to ensure the inclusion of relevant local studies.

Step 3: Study selection

To ensure consistent evaluation of the literature, a two-stage screening process was employed by two reviewers. This process involved initial screening of titles and abstracts, followed by a more in-depth review of full texts. Regarding any disagreements among the authors, they were resolved either through consensus or by the decision of one or two additional authors.

The process will be registered in a flowchart of the review process according to the PRISMA-ScR [43]. All studies were exported to the Rayyan Qatar Computing Research Institute (Rayyan®), and then deduplicated by one reviewer. The partial results of the text screening are available in Appendix II.

Inclusion criteria

To determine and choose pertinent publications concerning the topic, the subsequent inclusion criteria will be applied: (i) quantitative studies from database inception, such as correlational, ecological,

For peer review only

cohort, experimental, and cross-sectional studies, (ii) any individual or population groups exposed to wildfires, wildfire smoke or deforestation regardless of the exposure duration, (iii) any disease, hospitalization, or vital statistics, considered here as birth, death rate, and life expectancy, (iv) self-reported health condition. Only studies written in English, French, Portuguese and Spanish will be considered for inclusion.

Exclusion criteria

Studies will be excluded according to the following criteria: (i) theoretical studies; literature review (e.g., scoping review and systematic review), letter and editorials; (ii) qualitative studies (interviews, case studies etc.); (iii) environmental change only (e.g., extinction of wildlife, mosquitoes' habitats); (iv) air pollution only (e.g., air pollution from factories, mines, vehicles, without any relation to wildfires); (v) indoor fire; (vi) we will exclude studies solely focusing on health inequalities according to PROGRESS Plus; and (vii) studies in which the exposed population is entirely outside the tropical area, as delimited by the Tropic of Cancer and the Tropic of Capricorn.

The expansive nature of this review, encompassing diverse outcomes, countries, and databases, necessitates a streamlined approach. To ensure a comprehensive yet efficient exploration of the literature within the constraints of time and resources, we have opted to focus on quantitative studies. Quantitative studies often provide a more readily comparable data set, facilitating the data extraction and the synthesis of the existing research landscape in this complex field. Additionally, the exclusion of the grey literature also relies on the constraints of time and resources.

Step 4: Charting the data

The data of interest will be extracted with the data extraction tables and by filling out the data extraction form (in the Appendix III) in Microsoft Excel. During the pilot stage, two reviewers will independently conduct the task. Afterward, one reviewer will proceed, while the work will be reviewed by a second reviewer for quality assurance. The results will be categorised according to the review questions and charted in an iterative process, allowing the reviewers to continuously update these charts when additional unforeseen data are encountered. The data extraction table will be developed and tested, containing variables on the study reference (year of publication, author, journal, full title), intervention type, exposure, and data source (e.g., country, origin of the exposure data source), methods and findings (study design and modelling, health outcome, control, and treated group, point estimate, and causal identification strategy, lag between exposure and the health consequences and limitations of the study.

Step 5: Collating, summarising and reporting the results

For peer review only

All gathered data will be displayed in either tabular or diagrammatic formats to visually summarise the outcomes of the studies. Initially, a table containing comprehensive information about the selected papers will be provided, such as the number of studies, study design, exposure assessment (temporal and spatial scale, and data sources), statistical methods (statistical models and identification strategies), characteristics of study populations, and the countries where the studies were conducted. The data will be categorized separately for wildfires and deforestation. Different tables will be used to describe the detailed methods and data sources, followed by tables focused on findings and their subgroups. In case of studies investigating both wildfire and deforestation, we will summarize the data sources and methods used for the combined analysis and compare their effects on the outcomes. Therefore, we will synthesize the crude and adjusted effects for both analyses if available. These results will be meticulously presented, for instance, in tabular format to underscore the collaborative analysis, as wildfires and deforestation share common contributing factors. Finally, we will consider the overall implications of the results to ensure that the scoping review will provide relevant answers to the two main research questions previously posed.

Patient and public involvement

None.

ETHICS AND DISSEMINATION

The study is exempt from ethics review as it is meta-research and utilizes published, deidentified secondary data sources. The submission of results for publication in a peer-reviewed journal and presentation at scientific and policymakers' conferences is expected.

DISCUSSION

The resulting scoping review will offer novel insights by synthesizing a wide array of health outcomes associated with wildfires and deforestation. It will elucidate the methodologies and data sources utilized in existing literature to assess the impact of these phenomena on public health. Moreover, the review will provide policymakers with actionable recommendations derived from studies addressing the health effects of wildfires and deforestation, including considerations of magnitude and temporal lag between exposure and outcome.

While the scoping review will offer valuable insights, it is important to acknowledge that it may encounter certain limitations. The level of detail in the map defining the tropical rainforest biome will significantly influence which studies are included in the final selection. This can lead to the

For peer review only

unintended exclusion or inclusion of relevant research. The exclusion of the grey literature is contingent upon limitations in both time and resources available for the study. It often contains valuable insights and data that may not be found in traditional academic sources, potentially resulting in an incomplete understanding of the topic under investigation. Additionally, the scoping review will not conduct critical appraisal. This means they cannot assess the quality of included studies, potentially incorporating biased or flawed research. Consequently, drawing definitive conclusions about the effectiveness of interventions or pinpointing areas needing strong future studies becomes difficult. While valuable for initial exploration, these limitations necessitate cautious interpretation of the findings.

For peer review only

Contributors

G.C. is the primary and corresponding author and was responsible for the first and all subsequent drafts of this scoping review protocol. Study conception: J.F.S.F, P.J.C., J.O., S.V.K., L.E., R.F.S.A., G.F.S., M.L.B. Designed the search strategy: J.P., P.B.R., V.W., N.S.G., M.S. Data search: G.C., J.P., N.S.G., M.S., E.M.C.N. Text screening: G.C., T.C. Geographical map: G.C., D.J.D.N. All authors participated in discussions on the study design and critically revised drafts for improvements.

Competing interests

None.

Funding

This research was funded by the NIHR (NIHR134801) using UK aid from the UK Government to support global health research and by the Wellcome Trust grant (226306/Z/22/Z) awarded to the CIDACS Climate and Environmental Platform (CIDACS-Clima). The views expressed in this publication are those of the author(s) and not necessarily those of the NIHR or the UK government. JO, MS, VW and SVK are employed by the MRC/CSO Social and Public Health Sciences Unit, University of Glasgow, and supported by the Medical Research Council [grant numbers MC_UU_00022/2; MC_UU_00022/4; and Chief Scientist Office [grant numbers SPHSU17; SPHSU19]. The researchers were independent of the funders; the funders had no role in the study design, data collection, analysis and interpretation of data, the decision to publish, or the preparation of the manuscript.

References

[1] P. W. Ellis, T. Gopalakrishna, R. C. Goodman, F. E. Putz, A. Roopsind, P. M. Umunay, J. Zalman, E. A. Ellis, M. Karen, T. G. Gregoire and B. W. Griscom, “Reduced-impact logging for climate change

mitigation (RIL-C) can halve selective logging emissions from tropical forests," *Forest Ecology and Management,* vol. 438, 2019.

[2] L. Ying, H. Cheng, Z. Shen, P. Guan, C. Luo and X. Peng, "Relative humidity and agricultural activities dominate wildfire ignitions in Yunnan, Southwest China: Patterns, thresholds, and implications," *Agricultural and Forest Meteorology,* vol. 307, 2021.

[3] S. M. Hartinger, M. Yglesias-González, L. Blanco-Villafuerte, Y. K. Palmeiro-Silva, A. G. Lescano, A. Stewart-Ibarra, ... and M. Romanello, "The 2022 South America report of The Lancet Countdown on health and climate change: trust the science. Now that we know, we must act.," *The Lancet Regional Health–Americas,* vol. 20, 2023.

[4] C. Y. Shimamoto, A. A. Padial, C. M. Rosa and M. C. M. Marques, "Restoration of ecosystem services in tropical forests: A global meta-analysis," *Plos One,* vol. 13(12), 2018.

[5] D. Ellison, M. N. Futter and K. Bishop, "On the forest cover–water yield debate: from demand- to supply-sind thinking," *Global Change Biology,* vol. 18, 2012.

[6] F. B. F. Silvio, K. M. P. M. B. Ferraz, C. C. Cassiano, P. H. S. Brancalion, D. T. A. d. Luz, T. N. Azevedo, L. R. Tambosi and J. P. Metzger, "How good are tropical forest patches for ecosystem services?," *Landscape Ecology,* vol. 29, 2014.

[7] J. H. Ellwanger, B. Kulmann-Leal, V. L. Kaminski, J. Valverde-Villegas, A. B. G. VEIGA, F. R. Spilki, ... and J. A. B. Chies, "Beyond diversity loss and climate change: Impacts of Amazon deforestation on infectious diseases and public health," *Anais da Academia Brasileira de Ciências,* vol. 92.

[8] C. Vera, J. Baez, M. Douglas, C. B. Emmanuel, J. Marengo, J. Meitin, M. Nicolini, J. Nogues-Paegle, J. Paegle, O. Penalba, P. Salio, C. Saulo, M. A. Silva Dias, P. Silva Dias and E. Zipser, "The South American Low-Level Jet Experiment," *Bulletin of the American Meteorological Society,* vol. 87, 2006.

[9] D. M. Olson, E. Dinerstein, N. D. Wikramanayake, ... and K. Kassem, "Terrestrial ecoregions of the world: A new map of life on Earth," *BioScience,* vol. 51, no. 11, pp. 933-938, 2001.

[10] P. M. Lemieux, C. C. Lutes and D. A. Santoianni, "Emissions of organic air toxics from open burning: a comprehensive review," *Progress in energy and combustion science,* vol. 30, no. 1, 2004.

[11] R. Xu, P. Yu, M. J. Abramson, F. H. Johnston, J. M. Samet, M. L. Bell, ... and Y. Guo, "Wildfires, global climate change, and human health," *New England Journal of Medicine,* vol. 383, no. 22, pp. 2173-2181, 2020.

[12] D. Da Mata and M. Dotta, "Commodity booms and the environment," SSRN 3900793, 2021.

[13] P. Dasgupta, The economics of biodiversity: the Dasgupta review, Hm Treasury, 2021.

[14] J. Assunção, C. Gandour and R. Rocha, "Deforestation slowdown in the Brazilian Amazon: prices or policies?," *Environment and Development Economics,* vol. 20, pp. 697-722, 2015.

[15] Centre for Research on the Epidemiology of Disasters, United Nations Office for Disaster Risk Reduction, "The human cost of disasters: an overview of the last 20 years (2000-2019)," 2020.

[16] M. Adámek, Z. Jankovská, V. Hadincová, E. Kula and J. Wild , "Drivers of forest fire occurrence in the cultural landscape of Central Europe," *Landscape Ecology,* vol. 33, p. 2031–2045, 2018.

For peer review only

[17] K. Thonicke, S. Venevsky, S. Sitch and W. Cramer, "The Role of Fire Disturbance for Global Vegetation Dynamics: Coupling Fire into a," *Global Ecology and Biogeography,* vol. 10, pp. 661-677, 2001.

[18] C. Black and et al, "Wildfire smoke exposure and human health: Significant gaps in research for a growing public health issue," *Environmental toxicology and pharmacology,* vol. 55, pp. 186-195, 2017.

[19] R. Rocha and A. A. Sant'Anna, "Winds of fire and smoke: Air pollution and health in the Brazilian Amazon," *World Development,* vol. 151, p. 105722, 2022.

[20] E. Grant and J. D. Runkle, "Long-term health effects of wildfire exposure: a scoping review," *The Journal of Climate Change and Health,* vol. 6, p. 100110, 2022.

[21] S. C. D. C. Xavier, A. L. R. Roque, V. D. S. Lima, K. J. L. Monteiro, J. C. R. Otaviano, L. F. C. Ferreira da Silva and A. M. Jansen, "Lower richness of small wild mammal species and Chagas disease risk," *PLoS neglected tropical diseases,* vol. 6, no. 5, p. e1647.

[22] D. Lawrence and K. Vandecar, "Effects of tropical deforestation on climate and agriculture," *Nature climate change,* vol. 5, no. 1, pp. 27-36, 2015.

[23] P. Alliance, "Effects of Deforestation," [Online]. Available: https://pachamama.org/effects-of-deforestation. [Accessed 05 Mar 2024].

[24] B. F. A. d. Oliveira, M. J. Bottino, P. Nobre and C. A. Nobre, "Deforestation and climate change are projected to increase heat stress risk in the Brazilian Amazon," *COMMUNICATIONS EARTH & ENVIRONMENT,* 2021.

[25] R. Becerril-Piña and C. A. Mastachi-Loza, "Desertification: Causes and Countermeasures," in *Life on land*, Springer, 2020.

[26] E. Nanjappan, E. Sullo, S. Shrestha, S. Thomas and E. Nouvet, "Californian Wildfires and Associated Human Health Outcomes: An Epidemiological Scoping Review," *International Journal of Trend in Scientific Research and Development,* vol. 5, no. 5, pp. 944-953, 2021.

[27] P. To, E. Eboreime and V. I. Agyapong, "The impact of wildfires on mental health: a scoping review," *Behavioral Sciences,* vol. 11, no. 9, p. 126, 2021.

[28] C. C. Melton, C. M. Fries, R. M. Smith and L. R. Mason, "Wildfires and Older Adults: A Scoping Review of Impacts, Risks, and Interventions," *International journal of environmental research and public health,* vol. 20, no. 13, p. 6252, 2023.

[29] C. E. Reid, M. Brauer, F. H. Johnston, M. Jerrett, J. R. Balmes and C. T. Elliott, "Critical review of health impacts of wildfire smoke exposure," *Environmental health perspectives,* vol. 124, no. 9, pp. 1334-1343, 2016.

[30] T. M. Davey and L. A. Selvey, "Relationship between Land Use/Land-Use Change and Human Health in Australia: A Scoping Study," *International Journal of Environmental Research and Public Health,* vol. 17, 2020.

[31] M. Mastel, A. Bussalleu, V. A. Paz-Soldán, G. Salmón-Mulanovich, A. Valdés-Velásquez and S. M. Hartinger, "Critical linkages between land use change and human health in the Amazon region: A scoping review," *PloS one,* vol. 13, no. 6, p. e0196414, 2018.

[32] R. Leichenko and J. A. Silva, "Climate change and poverty: vulnerability, impacts, and alleviation strategies," *WIREs Climate Change,* vol. 5, 2014.

For peer review only

[33] S. Hallegatte, M. Fay and E. B. Barbier, "Poverty and climate change: introduction," *Environment and Development Economics,* vol. 23, 2018.

[34] R. Mendelsohn, A. Dinar and L. Williams, "The distributional impact of climate change on rich and poor countries," *Environment and Development Economics,* vol. 11, pp. 159-178, 2006.

[35] R. Davis, G. R. McGregor and K. B. Enfield, "Humidity: A review and primer on atmospheric moisture and human health," *Environmental research,* vol. 144, pp. 106-116, 2016.

[36] J. Gao, Y. Sun, Y. Lu and L. Li, "Impact of ambient humidity on child health: a systematic review," *PloS one,* vol. 9, no. 12, p. e112508, 2014.

[37] M. Osborne, "Wildfires Reached a Five-Year High in the Brazilian Amazon," Smithsonian Magazine, 9 September 2022. [Online]. Available: https://www.smithsonianmag.com/smart-news/wildfires-reached-a-five-year-high-in-the-brazilian-amazon-180980719/. [Accessed 31 October 2023].

[38] G. Alecrim, "Número de queimadas na Amazônia em 2022 supera 2021, mas é inferior ao recorde de 2004," CNN Brasil, 20 September 2022. [Online]. Available: https://www.cnnbrasil.com.br/nacional/numero-de-queimadas-na-amazonia-em-2022-supera-2021-mas-e-inferior-ao-recorde-de-2004. [Accessed 2023 October 31].

[39] N. Kshetri, D. C. Rojas Torres, H. Besada and M. A. Moros Ochoa, "Big data as a tool to monitor and deter environmental offenders in the global south: A multiple case study," *Sustainability,* vol. 12, no. 24, p. 10436, 2020.

[40] M. D. Peters, C. M. Godfrey, H. Khalil, P. McInerney, D. Parker and C. B. Soares, "Guidance for conducting systematic scoping reviews," *JBI Evidence Implementation,* vol. 13, no. 3, pp. 141-146, 2015.

[41] H. Arksey and L. O'Malley, "Scoping studies: towards a methodological framework," *International journal of social research methodology,* vol. 8, no. 1, pp. 19-32, 2005.

[42] D. Levac, H. Colquhoun and K. K. O'Brien, "Scoping studies: advancing the methodology," *mplementation science,* vol. 5, pp. 1-9, 2010.

[43] A. C. Tricco, E. Lillie, W. Zarin, K. K. O'Brien, H. Colquhoun, D. Levac, ... and S. E. Straus, "PRISMA extension for scoping reviews (PRISMA-ScR): checklist and explanation," *Annals of internal medicine,* vol. 169, no. 7, pp. 467-473, 2018.

[44] G. Casais, N. S. Guimarães, T. R. Cortes, J. Pescarini, P. R. d. Magalhães, V. Wells, J. F. d. Sousa Filho, D. J. D. Neves, M. Shimonovich, J. R. Olsen, E. M. d. Carvalho Neto, P. J. Cooper, S. V. Katikireddi, R. Andrade, G. F. d. Santos and M. L. Barreto, "Wildfire, deforestation, and health in tropical rainforest: a scoping review protocol," 2023. [Online]. Available: https://osf.io/pnqc7/.

[45] Nasa Earth Observatory, "Rainforest," [Online]. Available: https://earthobservatory.nasa.gov/biome/biorainforest.php.

FIGURE TITLE

Figure 1. Geolocation of all rainforest areas in the world

For peer review only

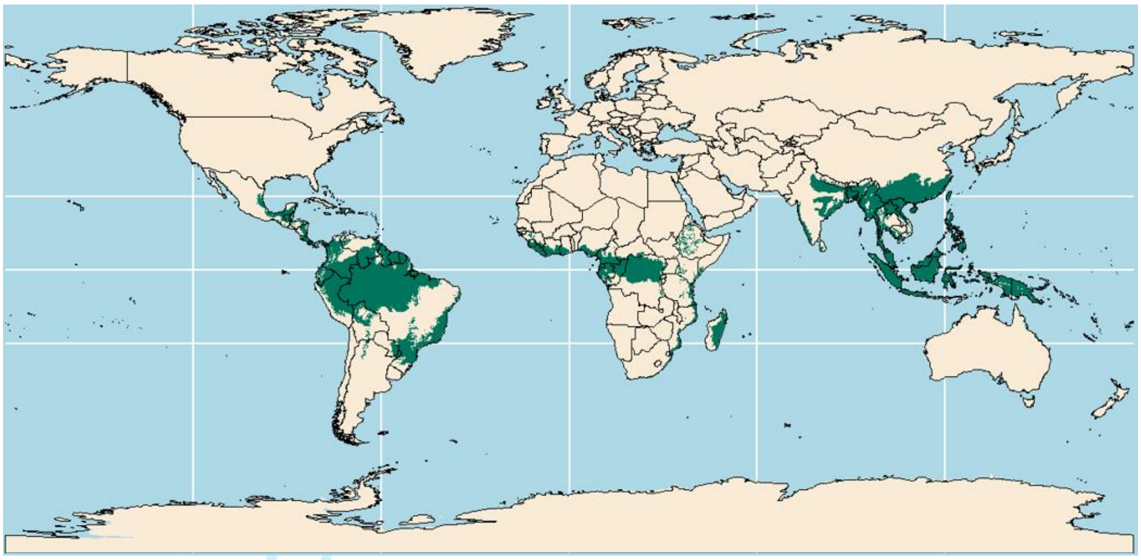

For peer review only

Appendices

Appendix I – Literature search

MEDLINE/PubMed

("wildfires"[MeSH Terms] OR "wildfires"[Text Word] OR "Wildfire"[All Fields] OR "Wildland Fires"[All Fields] OR "Brush Fires"[All Fields] OR "Brush Fire"[All Fields] OR "Forest Fires"[All Fields] OR "fire forest"[All Fields] OR "fires forest"[All Fields] OR "Forest Fire"[All Fields] OR "Wild Fires"[All Fields] OR "Wild Fire"[All Fields] OR "Fires"[MeSH Terms] OR "Fires"[Text Word] OR "Fire"[All Fields] OR "fire outbreaks"[All Fields] OR "Deforestation"[All Fields] OR "Grassfire"[All Fields] OR "prescribed burn"[All Fields] OR "prescribed fire"[All Fields]) AND ("Tropical Climate"[MeSH Terms] OR "Tropical Climate"[Text Word] OR "climate tropical"[All Fields] OR "climates tropical"[All Fields] OR "Tropical Climates"[All Fields] OR "Rainforest"[MeSH Terms] OR "Rainforest"[Text Word] OR "Rainforests"[All Fields] OR "Rain Forest"[All Fields] OR "forest rain"[All Fields] OR "Rain Forests"[All Fields] OR "Tropical Rainforest"[All Fields] OR "rainforest tropical"[All Fields] OR "rainforests tropical"[All Fields] OR "Tropical Rainforests"[All Fields] OR "Amazon"[All Fields] OR "Brazil"[All Fields] OR "Argentina"[All Fields] OR "Peru"[All Fields] OR "Ecuador"[All Fields] OR "Bolivia"[All Fields] OR "Colombia"[All Fields] OR "Venezuela"[All Fields] OR "Guyana"[All Fields] OR "Suriname"[All Fields] OR "French Guiana"[All Fields] OR "Paraguay"[All Fields] OR "Panama"[All Fields] OR "El Salvador"[All Fields] OR "Belize"[All Fields] OR "Costa Rica"[All Fields] OR "El Salvador"[All Fields] OR "Guatemala"[All Fields] OR "Honduras"[All Fields] OR "Nicaragua"[All Fields] OR "Panama"[All Fields] OR "Anguilla"[All Fields] OR "Antigua and Barbuda"[All Fields] OR "Aruba"[All Fields] OR "Bahamas"[All Fields] OR "Barbados"[All Fields] OR "British Virgin Islands"[All Fields] OR "Cayman Islands"[All Fields] OR "Cuba"[All Fields] OR "Dominica"[All Fields] OR "Dominican Republic"[All Fields] OR "Grenada"[All Fields] OR "Guadeloupe"[All Fields] OR "Haiti"[All Fields] OR "Jamaica"[All Fields] OR "Martinique"[All Fields] OR "Montserrat"[All Fields] OR "Netherlands Antilles"[All Fields] OR "Puerto Rico"[All Fields] OR "Saint Barthelemy"[All Fields] OR "Saint Kits and Nevis"[All Fields] OR "Saint Lucia"[All Fields] OR "Saint Martin"[All Fields] OR "Saint Vincent and the Grenadines"[All Fields] OR "Trinidad and Tobago"[All Fields] OR "United States Virgin Islands"[All Fields] OR "Mexico"[All Fields] OR "Hawaii"[All Fields] OR "Cape Verde"[All Fields] OR "Sao Tome and Principe"[All Fields] OR "Gambia"[All Fields] OR "Senegal"[All Fields] OR "Guinea-Bissau"[All Fields] OR "Guinea"[All Fields] OR "Sierra Leone"[All Fields] OR "Liberia"[All Fields] OR "Ivory Coast"[All Fields] OR "Ghana"[All Fields] OR "Togo"[All Fields] OR "Benin"[All Fields] OR "Nigeria"[All Fields] OR "Cameroon"[All Fields] OR "Central African Republic"[All Fields] OR "South Sudan"[All Fields] OR "Ethiopia"[All Fields] OR "Equatorial Guinea"[All Fields] OR "Gabon"[All Fields] OR "Congo"[All Fields] OR "Democratic Republic of the Congo"[All Fields] OR "Uganda"[All Fields] OR "Rwanda"[All Fields] OR "Burundi"[All Fields] OR "Kenya"[All Fields] OR "Somalia"[All Fields] OR "Tanzania"[All Fields] OR "Zambia"[All Fields] OR "Mozambique"[All Fields] OR "Madagascar"[All Fields] OR "Seychelles"[All Fields] OR "Mauritius"[All Fields] OR "Comoros"[All Fields] OR "Botswana"[All Fields] OR "Malawi"[All Fields] OR "Brunei"[All Fields] OR "Burma"[All Fields] OR "Myanmar"[All Fields] OR "Cambodia"[All Fields] OR "East Timor"[All Fields] OR

For peer review only

"Indonesia"[All Fields] OR "Laos"[All Fields] OR "Malaysia"[All Fields] OR "Philippines"[All Fields] OR "Singapore"[All Fields] OR "Thailand"[All Fields] OR "Vietnam"[All Fields] OR "India"[All Fields] OR "Papua New Guinea"[All Fields] OR "Australia"[All Fields] OR "Solomon Islands"[All Fields] OR "Vanuatu"[All Fields] OR "New Caledonia"[All Fields]) AND ("cardiovascular diseases"[MeSH Terms] OR "cardiovascular diseases"[Text Word] OR "Cardiovascular Disease"[All Fields] OR "disease cardiovascular"[All Fields] OR "Major Adverse Cardiac Events"[All Fields] OR "Cardiac Events"[All Fields] OR "Cardiac Event"[All Fields] OR "event cardiac"[All Fields] OR "Adverse Cardiac Event"[All Fields] OR "Adverse Cardiac Events"[All Fields] OR "cardiac events adverse"[All Fields] OR "chemically induced disorders"[MeSH Terms] OR "chemically induced disorders"[Text Word] OR "chemically induced disorders"[All Fields] OR "Chemically-Induced Disorder"[All Fields] OR "congenital, hereditary, and neonatal diseases and abnormalities"[MeSH Terms] OR "congenital hereditary and neonatal diseases and abnormalities"[Text Word] OR "Congenital Disorders"[All Fields] OR "disorder congenital"[All Fields] OR "disorders congenital"[All Fields] OR "Neonatal Diseases and Abnormalities"[All Fields] OR "Digestive System Diseases"[MeSH Terms] OR "Digestive System Diseases"[Text Word] OR "Digestive System Disease"[All Fields] OR "Digestive System Disorders"[All Fields] OR "Digestive System Disorder"[All Fields] OR "system disorders digestive"[All Fields] OR "Hepatobiliary Disorders"[All Fields] OR "Hepatobiliary Disorder"[All Fields] OR "Hepatobiliary Diseases"[All Fields] OR "Hepatobiliary Disease"[All Fields] OR "Disorders of Environmental Origin"[MeSH Terms] OR "Disorders of Environmental Origin"[Text Word] OR "Endocrine System Diseases"[MeSH Terms] OR "Endocrine System Diseases"[Text Word] OR "disease endocrine system"[All Fields] OR "diseases endocrine system"[All Fields] OR "Endocrine System Disease"[All Fields] OR "system disease endocrine"[All Fields] OR "system diseases endocrine"[All Fields] OR "Endocrine Diseases"[All Fields] OR "disease endocrine"[All Fields] OR "diseases endocrine"[All Fields] OR "Endocrine Disease"[All Fields] OR "Diseases of Endocrine System"[All Fields] OR "Eye Diseases"[MeSH Terms] OR "Eye Diseases"[Text Word] OR "Eye Disease"[All Fields] OR "Eye Disorders"[All Fields] OR "Eye Disorder"[All Fields] OR "Hemic and Lymphatic Diseases"[MeSH Terms] OR "Hemic and Lymphatic Diseases"[Text Word] OR "Blood and Lymphatic System Disorders"[All Fields] OR "Immune System Diseases"[MeSH Terms] OR "Immune System Diseases"[Text Word] OR "disease immune system"[All Fields] OR "Immune System Disease"[All Fields] OR "Immunologic Diseases"[All Fields] OR "disease immunologic"[All Fields] OR "Immunologic Disease"[All Fields] OR "Immunological Diseases"[All Fields] OR "disease immunological"[All Fields] OR "Immunological Disease"[All Fields] OR "Immune Diseases"[All Fields] OR "disease immune"[All Fields] OR "Immune Disease"[All Fields] OR "Diseases of Immune System"[All Fields] OR "Immune Disorders"[All Fields] OR "Immune Disorder"[All Fields] OR "Immune System Disorders"[All Fields] OR "disorder immune system"[All Fields] OR "Immune System Disorder"[All Fields] OR "Infections"[MeSH Terms] OR "Infections"[Text Word] OR "Infection and Infestation"[All Fields] OR "Infestation and Infection"[All Fields] OR "Infections and Infestations"[All Fields] OR "Infestations and Infections"[All Fields] OR "Infection"[All Fields] OR "Musculoskeletal Diseases"[MeSH Terms] OR "Musculoskeletal Diseases"[Text Word] OR "Musculoskeletal Disease"[All Fields] OR "Orthopedic Disorders"[All Fields] OR "Orthopedic Disorder"[All Fields] OR "Neoplasms"[MeSH Terms] OR "Neoplasms"[Text Word] OR "Tumor"[All Fields] OR "Neoplasm"[All Fields] OR "Tumors"[All Fields] OR "Neoplasia"[All Fields] OR "Neoplasias"[All Fields] OR "Cancer"[All Fields] OR "Cancers"[All Fields] OR "Malignant Neoplasm"[All Fields] OR "Malignancy"[All Fields] OR "Malignancies"[All Fields] OR "Malignant Neoplasms"[All Fields] OR "neoplasm malignant"[All Fields] OR "neoplasms malignant"[All Fields] OR "Benign

For peer review only

Neoplasms"[All Fields] OR "Benign Neoplasm"[All Fields] OR "neoplasms benign"[All Fields] OR "neoplasm benign"[All Fields] OR "Nervous System Diseases"[MeSH Terms] OR "Nervous System Diseases"[Text Word] OR "disease nervous system"[All Fields] OR "diseases nervous system"[All Fields] OR "Nervous System Disease"[All Fields] OR "Neurologic Disorders"[All Fields] OR "disorder neurologic"[All Fields] OR "disorders neurologic"[All Fields] OR "Neurologic Disorder"[All Fields] OR "Neurological Disorders"[All Fields] OR "disorder neurological"[All Fields] OR "disorders neurological"[All Fields] OR "Neurological Disorder"[All Fields] OR "Nervous System Disorders"[All Fields] OR "disorder nervous system"[All Fields] OR "disorders nervous system"[All Fields] OR "Nervous System Disorder"[All Fields] OR "Nutritional and Metabolic Diseases"[MeSH Terms] OR "Nutritional and Metabolic Diseases"[Text Word] OR "Occupational Diseases"[MeSH Terms] OR "Occupational Diseases"[Text Word] OR "disease occupational"[All Fields] OR "Occupational Disease"[All Fields] OR "Occupational Illnesses"[All Fields] OR "illnesses occupational"[All Fields] OR "diseases occupational"[All Fields] OR "Otorhinolaryngologic Diseases"[MeSH Terms] OR "Otorhinolaryngologic Diseases"[Text Word] OR "Otorhinolaryngological Disease"[All Fields] OR "Otorhinolaryngological Diseases"[All Fields] OR "Otolaryngological Diseases"[All Fields] OR "diseases otolaryngological"[All Fields] OR "Otolaryngological Disease"[All Fields] OR "Otorhinolaryngologic Disease"[All Fields] OR "ENT Diseases"[All Fields] OR "disease ent"[All Fields] OR "diseases ent"[All Fields] OR "ENT Disease"[All Fields] OR "Otolaryngologic Diseases"[All Fields] OR "disease otolaryngologic"[All Fields] OR "diseases otolaryngologic"[All Fields] OR "Otolaryngologic Disease"[All Fields] OR "pathological conditions, signs and symptoms"[MeSH Terms] OR "pathological conditions signs and symptoms"[Text Word] OR "Respiratory Tract Diseases"[MeSH Terms] OR "Respiratory Tract Diseases"[Text Word] OR "disease respiratory tract"[All Fields] OR "Respiratory Tract Disease"[All Fields] OR "Respiratory Diseases"[All Fields] OR "Respiratory System Diseases"[All Fields] OR "disease respiratory system"[All Fields] OR "Respiratory System Disease"[All Fields] OR "Skin and Connective Tissue Diseases"[MeSH Terms] OR "Skin and Connective Tissue Diseases"[Text Word] OR "Stomatognathic Diseases"[MeSH Terms] OR "Stomatognathic Diseases"[Text Word] OR "Stomatognathic Disease"[All Fields] OR "Mouth and Tooth Diseases"[All Fields] OR "Dental Diseases"[All Fields] OR "Dental Disease"[All Fields] OR "disease dental"[All Fields] OR "diseases dental"[All Fields] OR "Urogenital Diseases"[MeSH Terms] OR "Urogenital Diseases"[Text Word] OR "disease urogenital"[All Fields] OR "Urogenital Disease"[All Fields] OR "Genitourinary Diseases"[All Fields] OR "disease genitourinary"[All Fields] OR "Genitourinary Disease"[All Fields] OR "Wounds and Injuries"[MeSH Terms] OR "Wounds and Injuries"[Text Word] OR "Injuries and Wounds"[All Fields] OR "Wounds and Injury"[All Fields] OR "Injury and Wounds"[All Fields] OR "wounds injury"[All Fields] OR "Trauma"[All Fields] OR "Traumas"[All Fields] OR "injuries wounds"[All Fields] OR "research related injuries"[All Fields] OR "research related injuries"[All Fields] OR "Research-Related Injury"[All Fields] OR "Injuries"[All Fields] OR "Injury"[All Fields] OR "Wounds"[All Fields] OR "Wound"[All Fields] OR "Vital Statistics"[MeSH Terms] OR "Vital Statistics"[Text Word] OR "statistics vital"[All Fields] OR "Vital Statistics Registration"[All Fields] OR "Registration of Vital Statistics"[All Fields] OR "Vital Statistics Registrations"[All Fields] OR "registration vital statistics"[All Fields] OR "registrations vital statistics"[All Fields] OR "Patient Care"[MeSH Terms] OR "Patient Care"[Text Word] OR "care patient"[All Fields] OR "Informal care"[All Fields] OR "Informal cares"[All Fields] OR "care informal"[All Fields])

Date of search: August 18, 2023

For peer review only

Results: 2,296

Scopus

Search: ("wildfires" OR "wildfire" OR "wildland fires" OR "brush fires" OR "brush fire" OR "forest fires" OR "fire forest" OR "fires forest" OR "forest fire" OR "wild fires" OR "wild fire" OR "fires" OR "fire" OR "fire outbreaks" OR "deforestation" OR "grassfire" OR "prescribed burn" OR "prescribed fire")AND ("tropical climate" OR "climate tropical" OR "climates tropical" OR "tropical climates" OR "rainforest" OR "rainforests" OR "rain forest" OR "forest rain" OR "rain forests" OR "tropical rainforest" OR "rainforest tropical" OR "rainforests tropical" OR "tropical rainforests" OR "Amazon" OR "Brazil" OR "Argentina" OR "Peru" OR "Ecuador" OR "Bolivia" OR "Colombia" OR "Venezuela" OR "Guyana" OR "Suriname" OR "French Guiana" OR "Paraguay" OR "Panama" OR "El Salvador" OR "Belize" OR "Costa Rica" OR "El Salvador" OR "Guatemala" OR "Honduras" OR "Nicaragua" OR "Panama" OR "Anguilla" OR "Antigua and Barbuda" OR "Aruba" OR "Bahamas" OR "Barbados" OR "British Virgin Islands" OR "Cayman Islands" OR "Cuba" OR "Dominica" OR "Dominican Republic" OR "Grenada" OR "Guadeloupe" OR "Haiti" OR "Jamaica" OR "Martinique" OR "Montserrat" OR "Netherlands Antilles" OR "Puerto Rico" OR "Saint Barthelemy" OR "Saint Kits and Nevis" OR "Saint Lucia" OR "Saint Martin" OR "Saint Vincent and the Grenadines" OR "Trinidad and Tobago" OR "United States Virgin Islands" OR "Mexico" OR "Hawaii" OR "Cape Verde" OR "Sao Tome and Principe" OR "Gambia" OR "Senegal" OR "Guinea-Bissau" OR "Guinea" OR "Sierra Leone" OR "Liberia" OR "Ivory Coast" OR "Ghana" OR "Togo" OR "Benin" OR "Nigeria" OR "Cameroon" OR "Central African Republic" OR "South Sudan" OR "Ethiopia" OR "Equatorial Guinea" OR "Gabon" OR "Congo" OR "Democratic Republic of the Congo" OR "Uganda" OR "Rwanda" OR "Burundi" OR "Kenya" OR "Somalia" OR "Tanzania" OR "Zambia" OR "Mozambique" OR "Madagascar" OR "Seychelles" OR "Mauritius" OR "Comoros" OR "Botswana" OR "Malawi" OR "Brunei" OR "Burma" OR "Myanmar" OR "Cambodia" OR "East Timor" OR "Indonesia" OR "Laos" OR "Malaysia" OR "Philippines" OR "Singapore" OR "Thailand" OR "Vietnam" OR "India" OR "Papua New Guinea" OR "Australia" OR "Solomon Islands" OR "Vanuatu" OR "New Caledonia") AND ("cardiovascular diseases" OR "Cardiovascular Disease" OR "disease cardiovascular" OR "Major Adverse Cardiac Events" OR "Cardiac Events" OR "Cardiac Event" OR "event cardiac" OR "Adverse Cardiac Event" OR "Adverse Cardiac Events" OR "cardiac events adverse" OR "chemically induced disorders" OR "chemically induced disorders" OR "Chemically-Induced Disorder" OR "congenital, hereditary, and neonatal diseases and abnormalities" OR "Congenital Disorders" OR "disorder congenital" OR "disorders congenital" OR "Neonatal Diseases and Abnormalities" OR "Digestive System Diseases" OR "Digestive System Disease" OR "Digestive System Disorders" OR "Digestive System Disorder" OR "system disorders digestive" OR "Hepatobiliary Disorders" OR "Hepatobiliary Disorder" OR "Hepatobiliary Diseases" OR "Hepatobiliary Disease" OR "Disorders of Environmental Origin"OR "Endocrine System Diseases" OR "disease endocrine system" OR "diseases endocrine system" OR "Endocrine System Disease" OR "system disease endocrine" OR "system diseases endocrine" OR "Endocrine Diseases" OR "disease endocrine" OR "diseases endocrine" OR "Endocrine Disease" OR "Diseases of Endocrine System" OR "Eye Diseases" OR "Eye Disease" OR "Eye Disorders" OR "Eye Disorder" OR "Hemic and Lymphatic Diseases" OR "Blood and Lymphatic System Disorders" OR "Immune System Diseases" OR "disease immune system" OR "Immune System Disease" OR "Immunologic Diseases" OR "disease immunologic" OR "Immunologic Disease" OR "Immunological Diseases"

For peer review only

OR "disease immunological" OR "Immunological Disease" OR "Immune Diseases" OR "disease immune" OR "Immune Disease" OR "Diseases of Immune System" OR "Immune Disorders" OR "Immune Disorder" OR "Immune System Disorders" OR "disorder immune system" OR "Immune System Disorder" OR "Infections" OR "Infection and Infestation" OR "Infestation and Infection" OR "Infections and Infestations" OR "Infestations and Infections" OR "Infection" OR "Musculoskeletal Diseases" OR "Musculoskeletal Disease" OR "Orthopedic Disorders" OR "Orthopedic Disorder" OR "Neoplasms" OR "Tumor" OR "Neoplasm" OR "Tumors" OR "Neoplasia" OR "Neoplasias" OR "Cancer" OR "Cancers" OR "Malignant Neoplasm" OR "Malignancy" OR "Malignancies" OR "Malignant Neoplasms" OR "neoplasm malignant" OR "neoplasms malignant" OR "Benign Neoplasms" OR "Benign Neoplasm" OR "neoplasms benign" OR "neoplasm benign" OR "Nervous System Diseases" OR "disease nervous system" OR "diseases nervous system" OR "Nervous System Disease" OR "Neurologic Disorders" OR "disorder neurologic" OR "disorders neurologic" OR "Neurologic Disorder" OR "Neurological Disorders" OR "disorder neurological" OR "disorders neurological" OR "Neurological Disorder" OR "Nervous System Disorders" OR "disorder nervous system" OR "disorders nervous system" OR "Nervous System Disorder" OR "Nutritional and Metabolic Diseases" OR "Occupational Diseases" OR "disease occupational" OR "Occupational Disease" OR "Occupational Illnesses" OR "illnesses occupational" OR "diseases occupational" OR "Otorhinolaryngologic Diseases" OR "Otorhinolaryngological Disease" OR "Otorhinolaryngological Diseases" OR "Otolaryngological Diseases" OR "diseases otolaryngological" OR "Otolaryngological Disease" OR "Otorhinolaryngologic Disease" OR "ENT Diseases" OR "disease ent" OR "diseases ent" OR "ENT Disease" OR "Otolaryngologic Diseases" OR "disease otolaryngologic" OR "diseases otolaryngologic" OR "Otolaryngologic Disease" OR "pathological conditions, signs and symptoms" OR "Respiratory Tract Diseases" OR "disease respiratory tract" OR "Respiratory Tract Disease" OR "Respiratory Diseases" OR "Respiratory System Diseases" OR "disease respiratory system" OR "Respiratory System Disease" OR "Skin and Connective Tissue Diseases" OR "Stomatognathic Diseases" OR "Stomatognathic Disease" OR "Mouth and Tooth Diseases" OR "Dental Diseases" OR "Dental Disease" OR "disease dental" OR "diseases dental" OR "Urogenital Diseases" OR "disease urogenital" OR "Urogenital Disease" OR "Genitourinary Diseases" OR "disease genitourinary" OR "Genitourinary Disease" OR "Wounds and Injuries" OR "Injuries and Wounds" OR "Wounds and Injury" OR "Injury and Wounds" OR "wounds injury" OR "Trauma" OR "Traumas" OR "injuries wounds" OR "research related injuries" OR "research related injuries" OR "Research-Related Injury" OR "Injuries" OR "Injury" OR "Wounds" OR "Wound" OR "Vital Statistics" OR "statistics vital" OR "Vital Statistics Registration" OR "Registration of Vital Statistics" OR "Vital Statistics Registrations" OR "registration vital statistics" OR "registrations vital statistics" OR "Patient Care" OR "care patient" OR "Informal care" OR "Informal cares" OR "care informal")

Date of search: August 18, 2023

Results: 1,604

Biblioteca Virtual em Saúde

#1 "Incêndios Florestais" **OR** "Wildfires" OR "Incendios Forestales" OR "Incêndios" OR "Fires" OR "Incendios"

#2 "Clima Tropical" **OR** " Tropical Climate" **OR** "Floresta Úmida" OR "Rainforest" OR "Bosque Lluvioso" **OR** "Amazon" OR "Brazil" OR "Argentina" OR "Peru" OR "Ecuador" OR "Bolivia" OR "Colombia" OR "Venezuela" OR "Guyana" OR "Suriname" OR "French Guiana" OR "Paraguay" OR "Panama" OR "El Salvador" OR "Belize" OR "Costa Rica" OR "El Salvador" OR "Guatemala" OR "Honduras" OR "Nicaragua" OR "Panama" OR "Anguilla" OR "Antigua and Barbuda" OR "Aruba" OR "Bahamas" OR "Barbados" OR "British Virgin Islands" OR "Cayman Islands" OR "Cuba" OR "Dominica" OR "Dominican Republic" OR "Grenada" OR "Guadeloupe" OR "Haiti" OR "Jamaica" OR "Martinique" OR "Montserrat" OR "Netherlands Antilles" OR "Puerto Rico" OR "Saint Barthelemy" OR "Saint Kits and Nevis" OR "Saint Lucia" OR "Saint Martin" OR "Saint Vincent and the Grenadines" OR "Trinidad and Tobago" OR "United States Virgin Islands" OR "Mexico" OR "Hawaii" OR "Cape Verde" OR "Sao Tome and Principe" OR "Gambia" OR "Senegal" OR "Guinea-Bissau" OR "Guinea" OR "Sierra Leone" OR "Liberia" OR "Ivory Coast" OR "Ghana" OR "Togo" OR "Benin" OR "Nigeria" OR "Cameroon" OR "Central African Republic" OR "South Sudan" OR "Ethiopia" OR "Equatorial Guinea" OR "Gabon" OR "Congo" OR "Democratic Republic of the Congo" OR "Uganda" OR "Rwanda" OR "Burundi" OR "Kenya" OR "Somalia" OR "Tanzania" OR "Zambia" OR "Mozambique" OR "Madagascar" OR "Seychelles" OR "Mauritius" OR "Comoros" OR "Botswana" OR "Malawi" OR "Brunei" OR "Burma" OR "Myanmar" OR "Cambodia" OR "East Timor" OR "Indonesia" OR "Laos" OR "Malaysia" OR "Philippines" OR "Singapore" OR "Thailand" OR "Vietnam" OR "India" OR "Papua New Guinea" OR "Australia" OR "Solomon Islands" OR "Vanuatu" OR "New Caledonia"

#3 "Doenças Cardiovasculares" OR "Cardiovascular Diseases" OR "Enfermedades Cardiovasculares" OR "Distúrbios Induzidos Quimicamente" OR "Chemically-Induced Disorders" OR "Trastornos Químicamente Inducidos" OR "Doenças e Anormalidades Congênitas, Hereditárias e Neonatais" OR "Congenital, Hereditary, and Neonatal Diseases and Abnormalities" OR "Enfermedades y Anomalías Neonatales Congénitas y Hereditarias" OR "Doenças do Sistema Digestório" OR "Digestive System Diseases" OR "Transtornos de Origem Ambiental" OR "Disorders of Environmental Origin" OR "Trastornos de Origen Ambiental" OR "Doenças do Sistema Endócrino" OR "Endocrine System Diseases" OR "Enfermedades del Sistema Endocrino" OR "Oftalmopatias" OR "Eye Diseases" OR "Doenças Sanguíneas e Linfáticas" OR "Hemic and Lymphatic Diseases" OR "Enfermedades Hematológicas y Linfáticas" OR "Doenças do Sistema Imunitário" OR "Immune System Diseases" OR "Enfermedades del Sistema Inmune" OR "Infecções" OR "Infections" OR "Infecciones" OR "Doenças Musculoesqueléticas" OR "Musculoskeletal Diseases" OR "Enfermedades Musculoesqueléticas" OR "Neoplasias" OR "Neoplasms" OR "Doenças do Sistema Nervoso" OR "Nervous System Diseases" OR "Enfermedades del Sistema Nervioso" OR "Doenças Nutricionais e Metabólicas" OR "Nutritional and Metabolic Diseases" OR "Enfermedades Nutricionales y Metabólicas OR Doenças Profissionais" OR "Occupational Diseases" OR "Enfermedades Profesionales" OR "Otorrinolaringopatias" OR "Otorhinolaryngologic Diseases" OR "Enfermedades Otorrinolaringológicas" OR "Condições Patológicas, Sinais e Sintomas" OR "Pathological Conditions, Signs and Symptoms" OR "Condiciones Patológicas, Signos y Síntomas" OR "Doenças Respiratórias" OR "Respiratory Tract Diseases" OR "Enfermedades Respiratorias" OR "Doenças da Pele e do Tecido Conjuntivo" OR "Skin and Connective Tissue Diseases" OR "Enfermedades de la Piel y Tejido Conjuntivo" OR "Doenças Estomatognáticas" OR "Stomatognathic Diseases" OR "Enfermedades Estomatognáticas" OR "Doenças Urogenitais" OR "Urogenital Diseases" OR

"Enfermedades Urogenitales" OR "Ferimentos e Lesões" OR "Wounds and Injuries" OR "Heridas y Lesiones" OR "Estatísticas Vitais" OR "Vital Statistics" OR "Estadísticas Vitales" OR "Assistência ao Paciente" OR "Patient Care" OR "Atención al Paciente"

Detailed search:

("Incêndios Florestais" OR "Wildfires" OR "Incendios Forestales" OR "Incêndios" OR "Fires" OR "Incendios") AND ("Clima Tropical" OR " Tropical Climate" OR "Floresta Úmida" OR "Rainforest" OR "Bosque Lluvioso" OR "Amazon" OR "Brazil" OR "Argentina" OR "Peru" OR "Ecuador" OR "Bolivia" OR "Colombia" OR "Venezuela" OR "Guyana" OR "Suriname" OR "French Guiana" OR "Paraguay" OR "Panama" OR "El Salvador" OR "Belize" OR "Costa Rica" OR "El Salvador" OR "Guatemala" OR "Honduras" OR "Nicaragua" OR "Panama" OR "Anguilla" OR "Antigua and Barbuda" OR "Aruba" OR "Bahamas" OR "Barbados" OR "British Virgin Islands" OR "Cayman Islands" OR "Cuba" OR "Dominica" OR "Dominican Republic" OR "Grenada" OR "Guadeloupe" OR "Haiti" OR "Jamaica" OR "Martinique" OR "Montserrat" OR "Netherlands Antilles" OR "Puerto Rico" OR "Saint Barthelemy" OR "Saint Kits and Nevis" OR "Saint Lucia" OR "Saint Martin" OR "Saint Vincent and the Grenadines" OR "Trinidad and Tobago" OR "United States Virgin Islands" OR "Mexico" OR "Hawaii" OR "Cape Verde" OR "Sao Tome and Principe" OR "Gambia" OR "Senegal" OR "Guinea-Bissau" OR "Guinea" OR "Sierra Leone" OR "Liberia" OR "Ivory Coast" OR "Ghana" OR "Togo" OR "Benin" OR "Nigeria" OR "Cameroon" OR "Central African Republic" OR "South Sudan" OR "Ethiopia" OR "Equatorial Guinea" OR "Gabon" OR "Congo" OR "Democratic Republic of the Congo" OR "Uganda" OR "Rwanda" OR "Burundi" OR "Kenya" OR "Somalia" OR "Tanzania" OR "Zambia" OR "Mozambique" OR "Madagascar" OR "Seychelles" OR "Mauritius" OR "Comoros" OR "Botswana" OR "Malawi" OR "Brunei" OR "Burma" OR "Myanmar" OR "Cambodia" OR "East Timor" OR "Indonesia" OR "Laos" OR "Malaysia" OR "Philippines" OR "Singapore" OR "Thailand" OR "Vietnam" OR "India" OR "Papua New Guinea" OR "Australia" OR "Solomon Islands" OR "Vanuatu" OR "New Caledonia") AND ("Doenças Cardiovasculares" OR "Cardiovascular Diseases" OR "Enfermedades Cardiovasculares" OR "Distúrbios Induzidos Quimicamente" OR "Chemically-Induced Disorders" OR "Trastornos Químicamente Inducidos" OR "Doenças e Anormalidades Congênitas, Hereditárias e Neonatais" OR "Congenital, Hereditary, and Neonatal Diseases and Abnormalities" OR "Enfermedades y Anomalías Neonatales Congénitas y Hereditarias" OR "Doenças do Sistema Digestório" OR "Digestive System Diseases" OR "Transtornos de Origem Ambiental" OR "Disorders of Environmental Origin" OR "Trastornos de Origen Ambiental" OR "Doenças do Sistema Endócrino" OR "Endocrine System Diseases" OR "Enfermedades del Sistema Endocrino" OR "Oftalmopatias" OR "Eye Diseases" OR "Doenças Sanguíneas e Linfáticas" OR "Hemic and Lymphatic Diseases" OR "Enfermedades Hematológicas y Linfáticas" OR "Doenças do Sistema Imunitário" OR "Immune System Diseases" OR "Enfermedades del Sistema Inmune" OR "Infecções" OR "Infections" OR "Infecciones" OR "Doenças Musculoesqueléticas" OR "Musculoskeletal Diseases" OR "Enfermedades Musculoesqueléticas" OR "Neoplasias" OR "Neoplasms" OR "Doenças do Sistema Nervoso" OR "Nervous System Diseases" OR "Enfermedades del Sistema Nervioso" OR "Doenças Nutricionais e Metabólicas" OR "Nutritional and Metabolic Diseases" OR "Enfermedades Nutricionales y Metabólicas OR Doenças Profissionais" OR "Occupational Diseases" OR "Enfermedades Profesionales" OR "Otorrinolaringopatias" OR "Otorhinolaryngologic Diseases" OR "Enfermedades Otorrinolaringológicas" OR "Condições Patológicas, Sinais e Sintomas" OR

"Pathological Conditions, Signs and Symptoms" OR "Condiciones Patológicas, Signos y Síntomas" OR "Doenças Respiratórias" OR "Respiratory Tract Diseases" OR "Enfermedades Respiratorias" OR "Doenças da Pele e do Tecido Conjuntivo" OR "Skin and Connective Tissue Diseases" OR "Enfermedades de la Piel y Tejido Conjuntivo" OR "Doenças Estomatognáticas" OR "Stomatognathic Diseases" OR "Enfermedades Estomatognáticas" OR "Doenças Urogenitais" OR "Urogenital Diseases" OR "Enfermedades Urogenitales" OR "Ferimentos e Lesões" OR "Wounds and Injuries" OR "Heridas y Lesiones" OR "Estatísticas Vitais" OR "Vital Statistics" OR "Estadísticas Vitales" OR "Assistência ao Paciente" OR "Patient Care" OR "Atención al Paciente")

Date of search: August 18, 2023

Results: Lilacs (n = 44), Medline (n = 246), WPRIM (n = 3), BDENF – Enfermagem (n = 2), MINASPERÚ (n = 2), Recursos Multimídia (n = 2), BINACIS (n = 1), Desastres (n = 1), MedCarib (n = 1), PAHO-IRIS (n = 1), RDSM (n = 1), Coleciona SUS (n = 1).

Embase

#4

#1 AND #2 AND #3

4,019

#3

('wildfires'/syn OR 'fire'/syn OR 'deforestation'/syn) AND [embase]/lim

42,804

#2

('tropical climate'/syn OR 'climate tropical' OR 'climates tropical' OR 'tropical climates' OR 'rainforest'/syn OR 'rainforests' OR 'rain forest'/syn OR 'forest rain' OR 'rain forests' OR 'tropical rainforest'/syn OR 'rainforest tropical'/syn OR 'rainforests tropical' OR 'tropical rainforests' OR 'amazon'/syn OR 'brazil'/syn OR 'argentina'/syn OR 'peru'/syn OR 'ecuador'/syn OR 'bolivia'/syn OR 'colombia'/syn OR 'venezuela'/syn OR 'guyana'/syn OR 'suriname'/syn OR 'french guiana'/syn OR 'paraguay'/syn OR 'belize'/syn OR 'costa rica'/syn OR 'el salvador'/syn OR 'guatemala'/syn OR 'honduras'/syn OR 'nicaragua'/syn OR 'panama'/syn OR 'anguilla'/syn OR 'antigua and barbuda'/syn OR 'aruba'/syn OR 'bahamas'/syn OR 'barbados'/syn OR 'british virgin islands'/syn OR 'cayman islands'/syn OR 'cuba'/syn OR 'dominica'/syn OR 'dominican republic'/syn OR 'grenada'/syn OR 'guadeloupe'/syn OR 'haiti'/syn OR 'jamaica'/syn OR 'martinique'/syn OR 'montserrat'/syn OR 'netherlands antilles'/syn OR 'puerto rico'/syn OR 'saint barthelemy'/syn OR 'saint kits and nevis' OR 'saint lucia'/syn OR 'saint martin'/syn OR 'saint vincent and the grenadines'/syn OR 'trinidad and tobago'/syn OR 'united states virgin islands'/syn OR 'mexico'/syn OR 'hawaii'/syn OR 'cape verde'/syn OR 'sao tome and principe'/syn OR 'gambia'/syn OR 'senegal'/syn OR 'guinea-bissau'/syn OR 'guinea'/syn OR 'sierra leone'/syn OR 'liberia'/syn OR 'ivory coast'/syn OR 'ghana'/syn OR 'togo'/syn OR 'benin'/syn OR 'nigeria'/syn

For peer review only - http://bmjopen.bmj.com/site/about/guidelines.xhtml

OR 'cameroon'/syn OR 'central african republic'/syn OR 'south sudan'/syn OR 'ethiopia'/syn OR 'equatorial guinea'/syn OR 'gabon'/syn OR 'congo'/syn OR 'democratic republic of the congo'/syn OR 'uganda'/syn OR 'rwanda'/syn OR 'burundi'/syn OR 'kenya'/syn OR 'somalia'/syn OR 'tanzania'/syn OR 'zambia'/syn OR 'mozambique'/syn OR 'madagascar'/syn OR 'seychelles'/syn OR 'mauritius'/syn OR 'comoros'/syn OR 'botswana'/syn OR 'malawi'/syn OR 'brunei'/syn OR 'burma'/syn OR 'myanmar'/syn OR 'cambodia'/syn OR 'east timor'/syn OR 'indonesia'/syn OR 'laos'/syn OR 'malaysia'/syn OR 'philippines'/syn OR 'singapore'/syn OR 'thailand'/syn OR 'vietnam'/syn OR 'india'/syn OR 'papua new guinea'/syn OR 'australia'/syn OR 'solomon islands'/syn OR 'vanuatu'/syn OR 'new caledonia'/syn) AND [embase]/lim

4,621,441

#1

('patient care'/syn OR 'vital statistics'/syn OR 'wounds and injuries'/syn OR 'urogenital tract disease'/syn OR 'stomatognathic diseases'/syn OR 'skin and connective tissue diseases'/syn OR 'respiratory tract diseases'/syn OR 'pathological conditions, signs and symptoms'/syn OR 'otorhinolaryngologic diseases'/syn OR 'occupational diseases'/syn OR 'nutritional and metabolic diseases'/syn OR 'nervous system diseases'/syn OR 'neoplasms'/syn OR 'musculoskeletal disease'/syn OR 'infection'/syn OR 'immune system diseases'/syn OR 'hemic and lymphatic diseases'/syn OR 'eye diseases'/syn OR 'endocrine disease'/syn OR 'environmental disease'/syn OR 'digestive system diseases'/syn OR 'congenital, hereditary, and neonatal diseases and abnormalities'/syn OR 'chemically induced disorder'/syn OR 'cardiovascular diseases'/syn) AND [embase]/lim

Date of search: August 18, 2023

Records: 4,020

EconLit

(('wildfires' or 'fire' or 'deforestation') and (((((((((('tropical climate' or 'climate tropical' or 'climates tropical' or 'tropical climates' or 'rainforest' or 'rainforests' or 'rain forest' or 'forest rain' or 'rain
forests' or 'tropical rainforest' or 'rainforest tropical' or 'rainforests tropical' or 'tropical rainforests' or 'Amazon' or 'Brazil' or 'Argentina' or 'Peru' or 'Ecuador' or 'Bolivia' or 'Colombia' or 'Venezuela' or 'Guyana' or
'Suriname' or 'French Guiana' or 'Paraguay' or 'Panama' or 'El Salvador' or 'Belize' or 'Costa Rica' or 'El Salvador' or
'Guatemala' or 'Honduras' or 'Nicaragua' or 'Panama' or 'Anguilla' or 'Antigua) and Barbuda') or 'Aruba' or 'Bahamas' or
'Barbados' or 'British Virgin Islands' or 'Cayman Islands' or 'Cuba' or 'Dominica' or 'Dominican Republic' or 'Grenada'
or 'Guadeloupe' or 'Haiti' or 'Jamaica' or 'Martinique' or 'Montserrat' or 'Netherlands Antilles' or 'Puerto Rico' or
'Saint Barthelemy' or 'Saint Kits) and Nevis') or 'Saint Lucia' or 'Saint Martin' or 'Saint Vincent) and the

For peer review only

Grenadines') or 'Trinidad) and Tobago') or 'United States Virgin Islands' or 'Mexico' or 'Hawaii' or 'Cape Verde' or 'Sao Tome) and Principe') or 'Gambia' or 'Senegal' or 'Guinea-Bissau' or 'Guinea' or 'Sierra Leone' or 'Liberia' or 'Ivory Coast' or 'Ghana' or 'Togo' or 'Benin' or 'Nigeria' or 'Cameroon' or 'Central African Republic' or 'South Sudan' or 'Ethiopia' or 'Equatorial Guinea' or 'Gabon' or 'Congo' or 'Democratic Republic of the Congo' or 'Uganda' or 'Rwanda' or 'Burundi' or 'Kenya' or 'Somalia' or 'Tanzania' or 'Zambia' or 'Mozambique' or 'Madagascar' or 'Seychelles' or 'Mauritius' or 'Comoros' or 'Botswana' or 'Malawi' or 'Brunei' or 'Burma' or 'Myanmar' or 'Cambodia' or 'East Timor' or 'Indonesia' or 'Laos' or 'Malaysia' or 'Philippines' or 'Singapore' or 'Thailand' or 'Vietnam' or 'India' or 'Papua New Guinea' or 'Australia' or 'Solomon Islands' or 'Vanuatu' or 'New Caledonia') and (((((((((((('patient care' or 'vital statistics' or 'wounds) and injuries') or 'urogenital tract disease' or 'stomatognathic diseases' or 'skin) and connective tissue diseases') or 'respiratory tract diseases' or 'pathological conditions, signs) and symptoms') or 'otorhinolaryngologic diseases' or 'occupational diseases' or 'nutritional) and metabolic diseases') or 'nervous system diseases' or 'neoplasms' or 'musculoskeletal disease' or 'infection' or 'immune system diseases' or 'hemic) and lymphatic diseases') or 'eye diseases' or 'endocrine disease' or 'environmental disease' or 'digestive system diseases' or 'congenital, hereditary,) and neonatal diseases and abnormalities') or 'chemically induced disorder' or 'cardiovascular diseases')).mp. [mp=tx, bt, ti, ab, ct, hw, id]

Date of search: August 18, 2023

Records: 464

For peer review only

Appendix II - Ongoing work

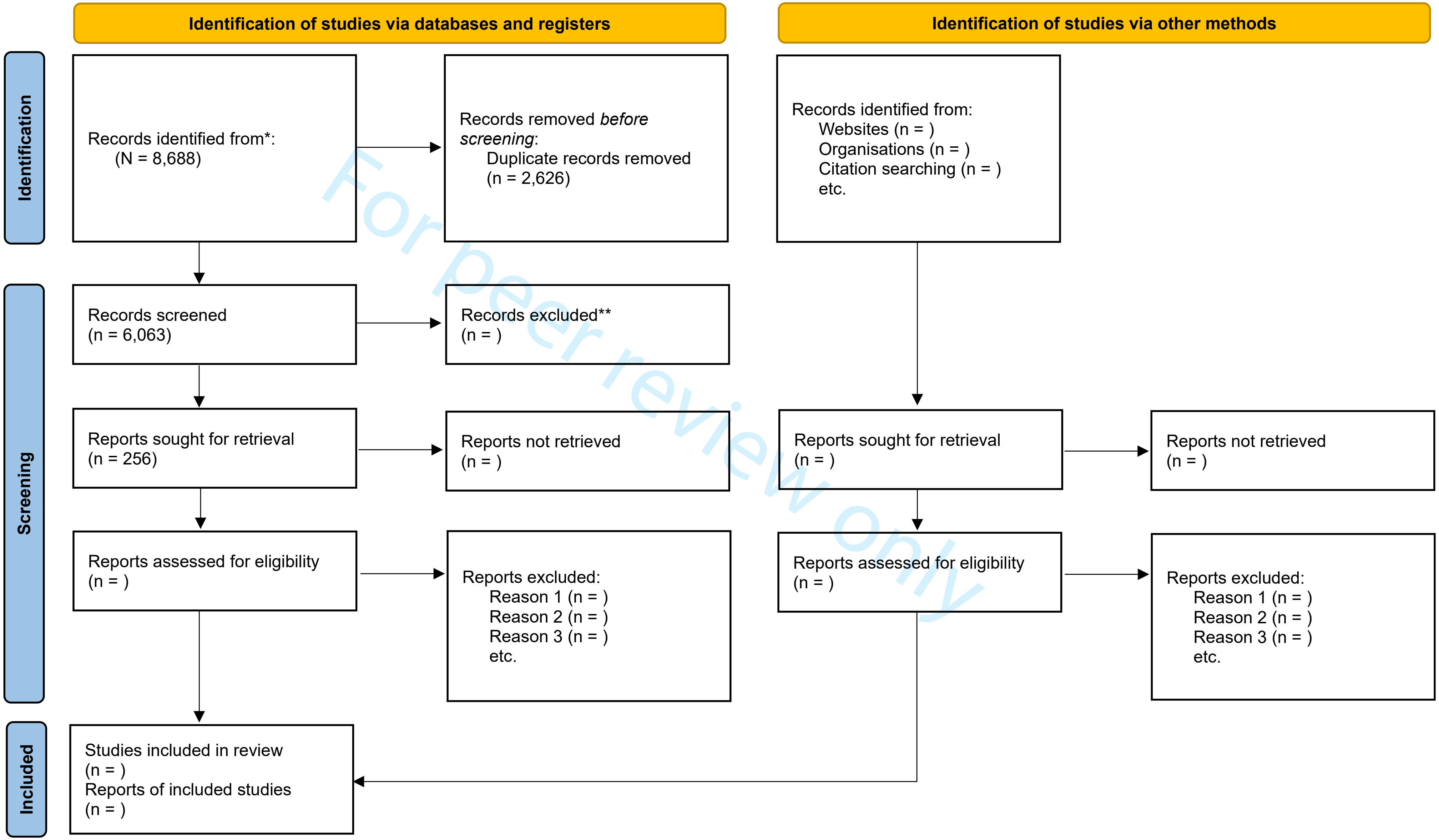

From: Page MJ, McKenzie JE, Bossuyt PM, Boutron I, Hoffmann TC, Mulrow CD, et al. The PRISMA 2020 statement: an updated guideline for reporting systematic reviews. BMJ 2021;372:n71. doi: 10.1136/bmj.n71.

For peer review only

Appendix III - Data extraction form

General information	
Reviewer who performed the data extraction:	
Date of the data extraction performed:	
Paper database:	
**Study identification**	
Publication full title:	
DOI number:	
First author name:	
Year of publication:	
Journal of publication:	
Aim of study:	
**Exposure and data source**	
Area (city, region, country):	
Exposure:	
Exposure temporal level:	
Exposure temporal duration:	
Origin of the exposure data source:	
Origin of the outcome data source:	
**Method**	
The statistical model applied:	
**Study design and findings**	
Study population:	
Health outcome:	
Type of disease:	
Lag between exposure and the health consequences	
Study findings as reported by the authors:	
Report the following list:<br>1. measure of association + standard errors for every different regression specification<br>2. Subgroups estimates<br>3. Confounders used<br>4. Identification strategy if appliable	
**Conclusions**	
Study limitations identified by the team:	
Policy recommendation from the study	

For peer review only
